# Isonitrile-responsive and bioorthogonally removable tetrazine protecting groups†
†Electronic supplementary information (ESI) available. See DOI: 10.1039/c9sc04649f


**DOI:** 10.1039/c9sc04649f

**Published:** 2019-11-05

**Authors:** Julian Tu, Dennis Svatunek, Saba Parvez, Hannah J. Eckvahl, Minghao Xu, Randall T. Peterson, K. N. Houk, Raphael M. Franzini

**Affiliations:** a Department of Medicinal Chemistry , College of Pharmacy , University of Utah , Salt Lake City , 84112 , USA . Email: Raphael.franzini@utah.edu; b Department of Chemistry and Biochemistry , University of California , Los Angeles , California 90095 , USA; c Department of Pharmacology and Toxicology , College of Pharmacy , University of Utah , Salt Lake City , 84112 , USA

## Abstract

*In vivo* compatible reactions have a broad range of possible applications in chemical biology and the pharmaceutical sciences. Here we report tetrazines that can be removed by exposure to isonitriles under very mild conditions. Tetrazylmethyl derivatives are easily accessible protecting groups for amines and phenols. The isonitrile-induced removal is rapid and near-quantitative. Intriguingly, the deprotection is especially effective with (trimethylsilyl)methyl isocyanide, and serum albumin can catalyze the elimination under physiological conditions. NMR and computational studies revealed that an imine-tautomerization step is often rate limiting, and the unexpected cleavage of the Si–C bond accelerates this step in the case with (trimethylsilyl)methyl isocyanide. Tetrazylmethyl-removal is compatible with use on biomacromolecules, in cellular environments, and in living organisms as demonstrated by cytotoxicity experiments and fluorophore-release studies on proteins and in zebrafish embryos. By combining tetrazylmethyl derivatives with previously reported tetrazine-responsive 3-isocyanopropyl groups, it was possible to liberate two fluorophores in vertebrates from a single bioorthogonal reaction. This chemistry will open new opportunities towards applications involving multiplexed release schemes and is a valuable asset to the growing toolbox of bioorthogonal dissociative reactions.

## Abstract 2

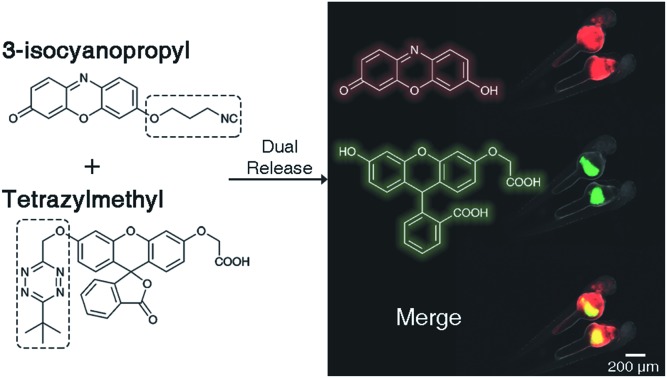
Tetrazylmethyl groups are reported here as bioorthogonal protecting groups that are readily removed by isonitriles, establishing a valuable addition to the dissociative bioorthogonal chemistry and synthetic methodology toolboxes.

## Introduction

Performing chemistry in living organisms with bioorthogonal reactions makes it possible to study biological processes in their natural environments.^1^,^2^ Recently, reactions have emerged that release diverse molecules under physiological conditions.^3^,^4^ These reactions have opened unprecedented possibilities in chemical biology and drug delivery.^5^,^6^ Dissociative bioorthogonal chemistry has been applied to the on-demand dissolution of polymers and micelles,^7^–^9^ site-specific actuation of prodrugs,^10^–^12^ and control of enzyme activity *in vivo*.^13^–^15^ Although a growing number of “click-to-release” reactions^16^–^23^ has provided a solid foundation for applications in the life sciences, extending the reaction scope will be necessary to access the full range of capabilities. Moreover, there is a need for chemistry to allow for the controlled and simultaneous release of more than one molecule.^24^ Dual-release reactions could be used for the concomitant delivery of synergistic drugs, in theranostic applications, and in multiplexed detection schemes.

**Fig. 1:**
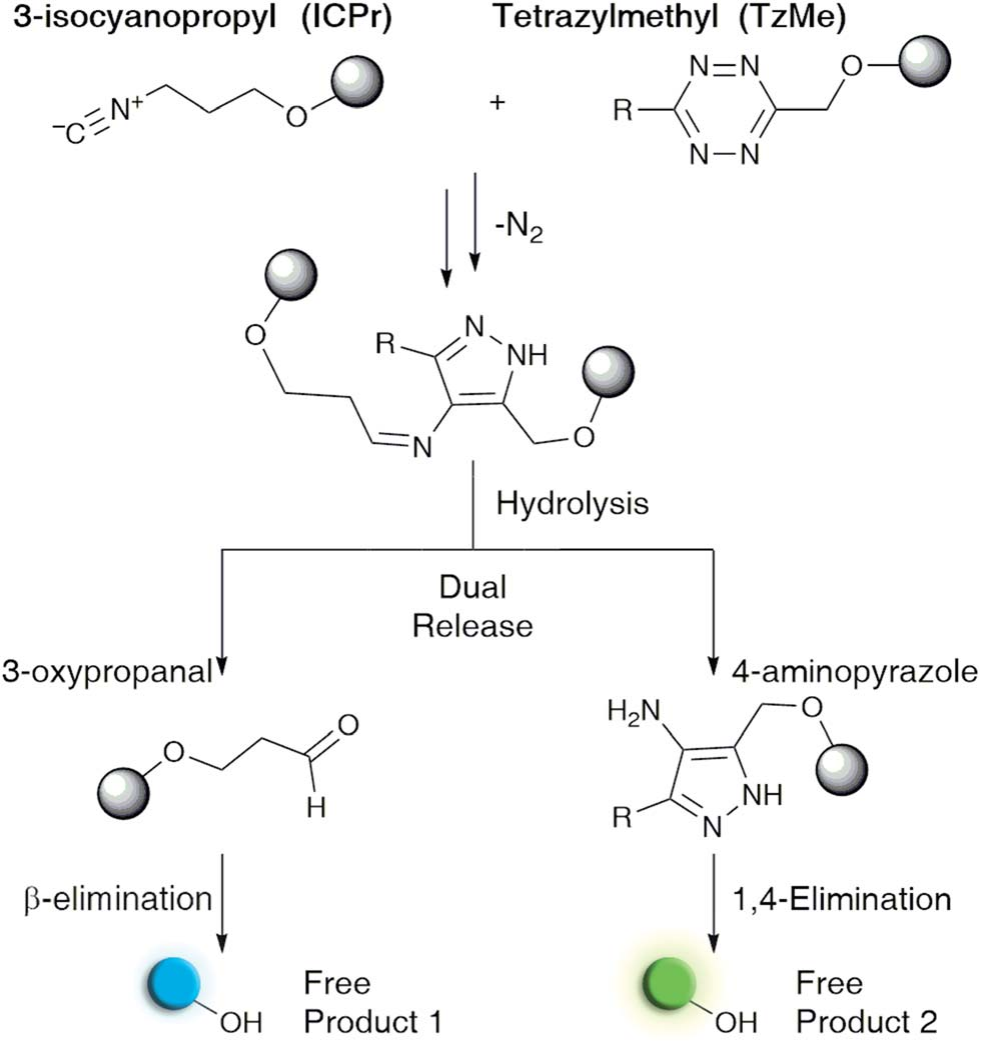
Proposed reaction to achieve the dual release of biological effectors from previously reported 3-isocyanopropyl (ICPr) and tetrazylmethyl (TzMe) derivatives developed herein.

Bioorthogonal chemistry, both ligating and dissociative, mainly revolves around pericyclic reactions.^25^,^26^ In particular, inverse-electron demand cycloadditions offer rapid reaction kinetics and high biocompatibility.^27^,^28^ 1,2,4,5-Tetrazines are the most prevalent dienes in such reactions.^29^,^30^ These heterocycles react with and subsequently trigger the release of payloads from allyl-modified *trans*-cyclooctenes,^17^,^31^–^33^ benzonorbornadiene derivatives,^21^,^34^ and vinyl ethers.^7^,^19^,^20^ Tetrazines also undergo bioorthogonal cycloaddition reactions with isonitriles,^35^,^36^ and we have recently shown that they can induce the release of payloads from 3-isocyanopropyl (ICPr) groups (Fig. 1).^37^,^38^

Given the prominent role and favorable properties of tetrazine-based cycloadditions in dissociative bioorthogonal chemistry, it would be valuable to have tetrazine-based protecting groups that release a payload upon reaction with some of these dienophiles. An example of such a molecule was disclosed by Wang *et al.* as demonstrated by a tetrazine-based prodrug that was activated through a reaction with a cyclooctyne modified with a hydroxyl group at the propargylic position.^23^ Such tetrazine derivatives, when combined with complementary release reagents, could be used for dual-release applications. Running two bioorthogonal release reactions in parallel is one possibility to achieve such dual-release as has been demonstrated by combining the reaction of tetrazines and benzonorbornadienes with that between sulfonyl sydnonimines and dibenzoazacyclooctyne.^39^ A second example involved the reaction between vinyl ethers and tetrazines, which released alcohols but was limited to generating pyridazine and had slow reaction kinetics.^24^ A single pair of reactants that releases two molecules in a single fast reaction would bring such approaches to the next level.

**Fig. 2:**
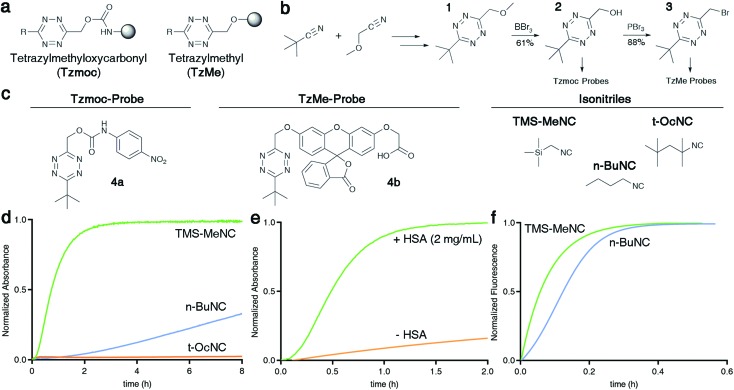
Isonitrile-mediated uncaging of amines and phenols from Tzmoc and TzMe derivatives. (a) Structures of tetrazylmethyloxycarbonyl (Tzmoc) and tetrazylmethyl (TzMe) groups used to cage amines and phenols, respectively. (b) Synthesis of Tzmoc or TzMe-caged probes (conditions and yields described in the ESI†). (c) Structures of reporter probes and isonitrile triggers used in this study. (d) Kinetics of pNA release from **4a** triggered by different isonitriles (c(**4a**) = 0.2 mM, c(R–NC) = 2 mM, DMSO : PBS pH 7.4 (4 : 1, v/v), *T* = 37 °C, *λ* = 435 nm, *n* = 3). (e) Kinetics of pNA release from **4a** triggered by *n*-BuNC catalysed by serum albumin (c(**4a**) = 8 μM, c(*n*-BuNC) = 6 mM, c(HSA) = 2 mg mL^–1^, DMSO : PBS pH 7.4 (1 : 4, v/v), *T* = 37 °C, *λ* = 385 nm, *n* = 3). (f) Kinetics of *O*-carboxymethyl fluorescein release from **4b** triggered by TMS-MeNC or *n*-BuNC (c(**4b**) = 8 μM, c(R–NC) = 6 mM, DMSO : PBS pH 7.4 (1 : 4, v/v), *T* = 37 °C, *λ*_ex_ = 488 nm, *λ*_em_ = 520 nm, *n* = 3).

Here we describe tetrazylmethyl (TzMe) protecting groups that can be rapidly removed by a reaction with isonitriles. The rationale behind our design is based on the precedent that isonitriles convert tetrazines into 4-aminopyrazoles^35^,^36^ and that 5-membered heterocycles with amine substituents spontaneously eliminate diverse functional groups.^40^,^41^ In a series of experiments, we demonstrated that TzMe-modified molecules reacted readily with isonitriles to release amines (from tetrazylmethyloxycarbonyl (Tzmoc) derivatives) and phenols (Fig. 2a). We analyzed the reaction mechanism, and in the case of (trimethylsilyl)methyl isocyanide (TMS-MeNC), observed an intriguing C–Si bond cleavage that accelerated release. The reaction was compatible with living systems, and we demonstrated that when TzMe-derivatives were combined with ICPr-derivatives (Fig. 1),^37^ two fluorophores could be simultaneously released in zebrafish embryos. This innovative chemistry will open new possibilities for biomedical research and drug delivery.

## Results and discussion

### Isonitrile-induced removal of Tzmoc-groups from amines

To prove the concept of isonitrile-induced deprotection of TzMe groups, we synthesized a colorimetric reporter probe (**4a**, Fig. 2b and c) consisting of *p*-nitroaniline (pNA) caged by a Tzmoc group (Fig. 2a). pNA has a characteristic maximum absorbance signal at *λ*_abs_ = 385 nm, which is hypsochromically shifted in derivatives with acyl-modified amine. **4a** was accessed by a dibutyltin dilaureate-catalyzed reaction of (6-(*tert*-butyl)-1,2,4,5-tetrazin-3-yl)methanol (**2**) with 4-nitrophenyl isocyanate (Fig. 2b). **2** was prepared in three steps from the nitrile precursors to obtain **1**, followed by deprotection of the methoxy group by BBr_3_ (Fig. 2b).

**Fig. 3:**
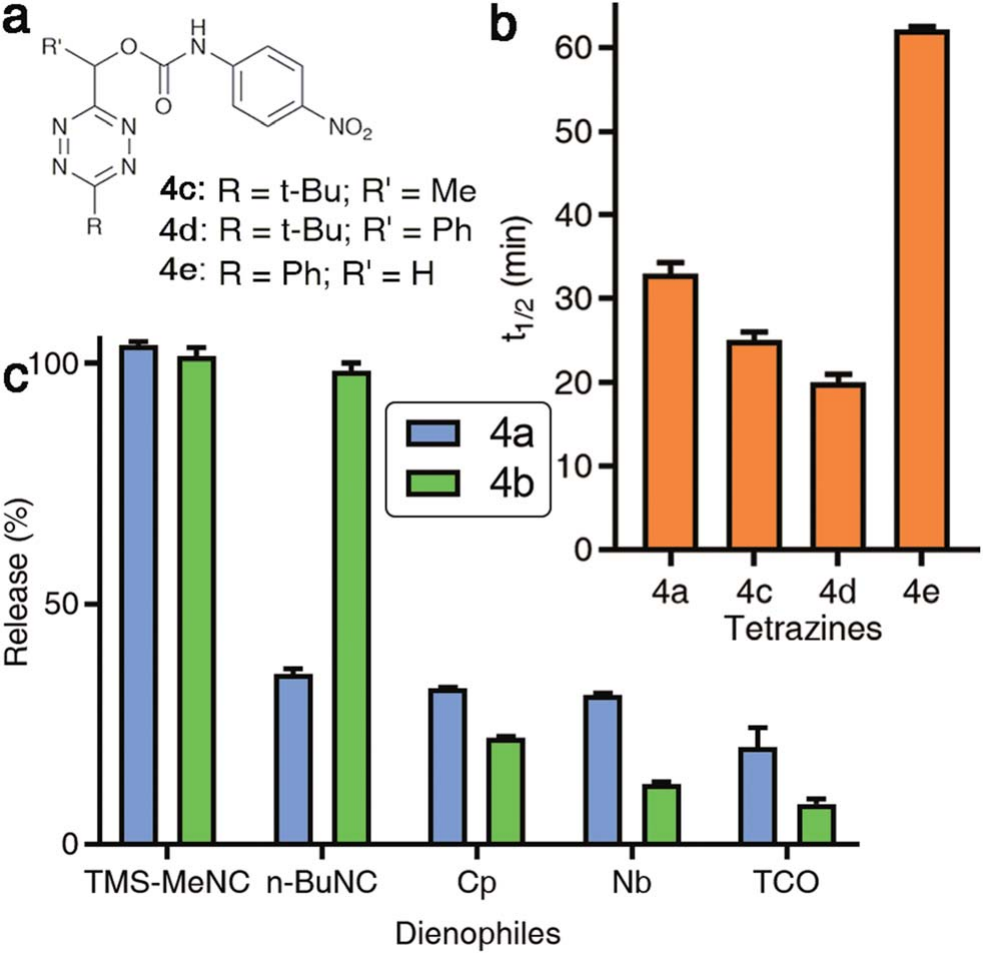
Effect of structural modifications to tetrazines on isonitrile-induced removal. (a) Structures of modified Tzmoc probes with a pNA reporter molecule. (b) Half-lives of the TMS-MeNC mediated Tzmoc deprotection (*t*_1/2_ of release of pNA) from probes **4a**, **4c**, **4d**, and **4e** (c(**4a–e**) = 0.2 mM, c(TMS-MeNC) = 2 mM, DMSO : PBS pH 7.4 (4 : 1, v/v), *T* = 37 °C). (c) Release yields of pNA or *O*-carboxymethyl fluorescein from **4a** or **4b**, respectively, triggered by several dienophiles (structures shown in Fig. S10 in the ESI†); pNA release: see Fig. 2d; *t* = 8 h; *O*-carboxymethyl fluorescein release: c(**4b**) = 8 μM, c(dienophile) = 2 mM, DMSO : PBS pH 7.4 (1 : 4, v/v), *T* = 37 °C, *λ*_ex_ = 488 nm, *λ*_em_ = 520 nm, *t* = 8 h.

We evaluated the liberation of pNA from **4a** upon reaction with several isonitriles (Fig. 2d). As designed, a primary isonitrile, *n*-butyl isocyanide (*n*-BuNC, Fig. 2c), reacted with the tetrazine and elicited the release of pNA as monitored by the emergence of the pNA absorbance signal (Fig. 2d). As a control, we performed the experiment with *tert*-octyl isocyanide (*t*-OcNC, Fig. 2c), which we expected not to release pNA because tertiary isonitriles form stable 4*H*-pyrazol-4-imine conjugates.^36^,^38^,^42^ Indeed, pNA-release was undetectable in experiments with *t*-OcNC confirming that TzMe-removal follows the designed release principles. We were interested whether electron-donating groups adjacent to the isocyano functionality would accelerate the inverse-electron demand cycloaddition step. We therefore tested the reaction of **4a** with (trimethylsilyl)methyl isocyanide (TMS-MeNC, Fig. 2c). As predicted, TMS-MeNC reacted ∼3-fold faster with **4a** (*k*_2_ = 0.344 ± 0.013 M^–1^ s^–1^) than did *n*-BuNC (*k*_2_ = 0.117 ± 0.001 M^–1^ s^–1^), and an isonitrile with an electron-withdrawing substituent (methyl isocyanoacetate) lead to a 2-fold decrease (*k*_2_ = 0.05 ± 0.01 M^–1^ s^–1^) in the cycloaddition rate (Table S1†) but still released pNA (data not shown). Unexpectedly however, the TMS-substituent also greatly accelerated the release step (Fig. 2d). The rate of pNA release was ∼30-fold faster for TMS-MeNC (*k*_1_ = 3.4 × 10^–4^ ± 1.1 × 10^–6^ s^–1^) than for *n*-BuNC (*k*_1_ = 1.1 × 10^–5^ ± 1.2 × 10^–7^ s^–1^). Reactions with *n*-BuNC led to gradual, continuous, elimination of pNA with a release yield of 35.4 ± 1.0% measured at the 8 hour time-point in contrast to reactions with TMS-MeNC leading to near-quantitative release yields in this period as quantified by the absorbance signal (Fig. 3c). The bimolecular reaction rates of tetrazines and isonitriles were in the range of those observed in previous studies (Table S1†).^37^,^38^ Under these conditions, (DMSO : PBS pH 7.4, 4 : 1, v/v at *T* = 37 °C) the rate constants of the reactions with **4a** ranged from *k*_2_ = 0.05–0.38 M^–1^ s^–1^. The water content strongly influences the kinetics of the cycloaddition step, and based on previous studies,^34^,^38^ we extrapolate the reaction to be about 10-fold faster in purely aqueous solutions. These initial results indicate that the TMS-group promotes pNA release as the faster bimolecular rate of TMS-MeNC compared to *n*-BuNC is insufficient to explain the rapid elimination of pNA for the former.

Although TMS-MeNC effectively elicits the release of amines from Tzmoc groups, there are applications for which the rapid release by simple alkyl isocyanides will be preferred. For example, TzMe-molecules could be combined with ICPr derivatives^37^ in dual-release strategies (Fig. 1). Serum albumins catalyze diverse chemical transformations,^43^–^45^ and we hypothesized that albumin might also accelerate the release step. Indeed, both human serum albumin (2 mg mL^–1^ HSA in 4 : 1, PBS pH 7.4 : DMSO at *T* = 37 °C) and bovine serum albumin at 2 mg mL^–1^ (data not shown) greatly accelerated the liberation of pNA in reactions with *n*-BuNC (Fig. 2e). *n*-BuNC was able to effectively elicit the near-quantitative release of pNA in a little over an hour while the same reaction without HSA led to less than 20% release during the same timeframe. In contrast, **4a** incubated alone in a solution of HSA (2 mg mL^–1^ in PBS, *T* = 37 °C) did not result in a detectable pNA release signal (data not shown), indicating that HSA catalyzes the elimination step whereas the Tzmoc-probe is stable in the absence of isonitrile.

To differentiate between a catalytic activity of the protein and simple base-catalysis by its surface amines, we tested the effect of tris-base (concentration equal to that of surface amines in HSA experiments; 2 mM) on the release rate of pNA. The base had no detectable effect on the isonitrile-induced release of pNA from **4a** (Fig. S1†). It is therefore possible to achieve rapid and high-yielding uncaging of amines from stable Tzmoc precursors with simple alkyl isocyanides in serum.

### Isonitrile-induced removal of TzMe-groups from phenols

Having demonstrated the release of carbamates from Tzmoc-groups, we aimed to determine whether the chemistry would be applicable to other functional groups. We were especially interested in phenols because aromatic hydroxy groups are present in tyrosine, diverse drugs, and fluorophores. For these experiments, we synthesized a TzMe-caged *O*-carboxymethyl fluorescein (**4b**, Fig. 2c) and 7-hydroxycoumarin (**4b′**, Fig. S2†) that report on TzMe-removal by a fluorescence turn-on signal.^46^,^47^ The fluorogenic probes were synthesized by etherification of the phenolic dyes with 3-(bromomethyl)-6-(*tert*-butyl)-1,2,4,5-tetrazine (**3**), which can be accessed by bromination (PBr_3_; 88%) of **2** (Fig. 2b). The reaction of TMS-MeNC with **4b** (Fig. 2f) and the 7-hydroxycoumarin derivative **4b′** (Fig. S3†) led to near-quantitative release yields by 2 h as quantified by fluorescence emission and HPLC analysis (Fig. S4†). TzMe-removal was associated with a characteristic fluorescence increase (152-fold for **4b′** and 30-fold for **4b**; Fig. S5†). The kinetics of the release reaction of **4b** with TMS-MeNC was determined by measuring the fluorescence turn-on signal (*k*_1_ = 3.5 × 10^–3^ ± 1.7 × 10^–4^ s^–1^). The faster release rate compared to the release of **4a** may in part be explained by the higher water content. Surprisingly, the rate of 7-hydroxycoumarin (Fig. S3†) and *O*-carboxymethyl fluorescein (Fig. 2f) elimination by *n*-BuNC was significantly faster than for **4a** without the need for the addition of albumin, an effect that was assessed by NMR studies. We further evaluated the stability of TzMe-caged probes. First, we assessed a TzMe-caged 7-hydroxycoumarin dye (**4b′**, Fig. S2†) in a human liver microsome stability assay (Creative Bioarray, USA) and the probe exhibited good stability (*t*_1/2_ = 52.11 min; Cl_int_ = 33.36 mL min^–1^ kg^–1^; Fig. S6†) even under these harsh conditions. Second, the TzMe-derivative of fluorescein (**4b**) used for zebrafish studies was stable in serum for hours (*t*_1/2_ = 19 ± 4 h). The decomposition product was not the released fluorophore and therefore the contribution to fluorescence background is low (Fig. S7†). These experiments establish that TzMe-groups are removed rapidly and in high yields from key functional groups.

### Effect of structural modifications on isonitrile-induced TzMe deprotection

We were interested to determine if the reaction kinetics and release yields from isonitrile-induced release from TzMe-groups could be enhanced by modifying the structure of the tetrazine. We designed a series of tetrazyl-derivatives of pNA (Fig. 3a) and analyzed such parameters upon reaction with isonitriles *n*-BuNC and TMS-MeNC. Methyl and phenyl groups at the methylene position (**R′** in Fig. 3a) modestly accelerated the release of pNA upon reaction with TMS-MeNC (Fig. 3b) with minor impact on the bimolecular kinetics (Table S1†). A methyl substituted tetrazine (**4c**, Fig. 3a) released pNA with a half-life of 24 min and a phenyl-substituted derivative (**4d**, Fig. 3a) with a half-life of 19 min, both near-quantitatively (Fig. 3b). Intriguingly, modifications drastically decreased the ability of *n*-BuNC to trigger the release of pNA; after 8 h only 20.2 ± 0.8% and 6.9 ± 0.4% of the pNA was deprotected from **4c** and **4d**, respectively (Fig. S8†). The effect on the bimolecular reaction rates did not cause the modest release yields of pNA (Table S1†). Next, replacing the C-6 *tert*-butyl group of **4a** by a phenyl substituent (**4e**, Fig. 3a) led to a marginally faster release of pNA triggered by *n*-BuNC (Fig. S9†). However, the phenyl group decreased the rate of TMS-MeNC triggered release (Fig. 3b). This effect may in part be because of a slightly slowed bimolecular reaction rate (Table S1†), which agrees with the lack of dispersion forces between the *tert*-butyl group and the incoming isocyano group.^38^ These results show that the substituents on both the tetrazyl ring and the methylene position are important to achieve prompt and high-yielding release. These insights provide guidance for further improvement of probe performance in future studies.

### TzMe removal by alternative dienophiles

Inspired by the effective release of payloads from TzMe-derivatives by isonitriles, it was of interest to find out whether other dienophiles provide a similar outcome. Tetrazines react with diverse strained alkenes,^29^,^30^ and we tested whether such dienophiles (methylcyclopropene (Cp), norbornene (Nb), and *trans*-cyclooctene (TCO); see Fig. S10† for structures) induce the release of pNA from **4a**. Elimination of pNA occurred; however, the yields of amine release were modest (*t* = 8 h, 37 °C; Cp = 32.4 ± 0.2%; Nb = 31.0 ± 0.3%; TCO = 20.2 ± 4.0%; Fig. 3c). Addition of HSA, which catalyzed pNA release from **4a** (Fig. 2e) in the reaction with *n*-BuNC, had an insignificant effect on the TCO-mediated reaction (data not shown). We further tested the ability of Cp, Nb, and TCO to elicit the release of *O*-carboxymethyl fluorescein from **4b**. Analogous to the results for pNA release, only a fraction of the product was eliminated (*t* = 8 h, 37 °C; Cp = 22.1 ± 0.3%; Nb = 12.4 ± 0.5%; TCO = 8.3 ± 1.0%; Fig. 3c). These experiments demonstrate that isonitriles have a unique ability to remove TzMe-based protecting groups. In the case of TCO, the rapid bimolecular reaction with tetrazines may open opportunities for interesting applications in drug delivery where the rate of the release step may not be limiting.

### Studies on the mechanism of TzMe removal

**Fig. 4:**
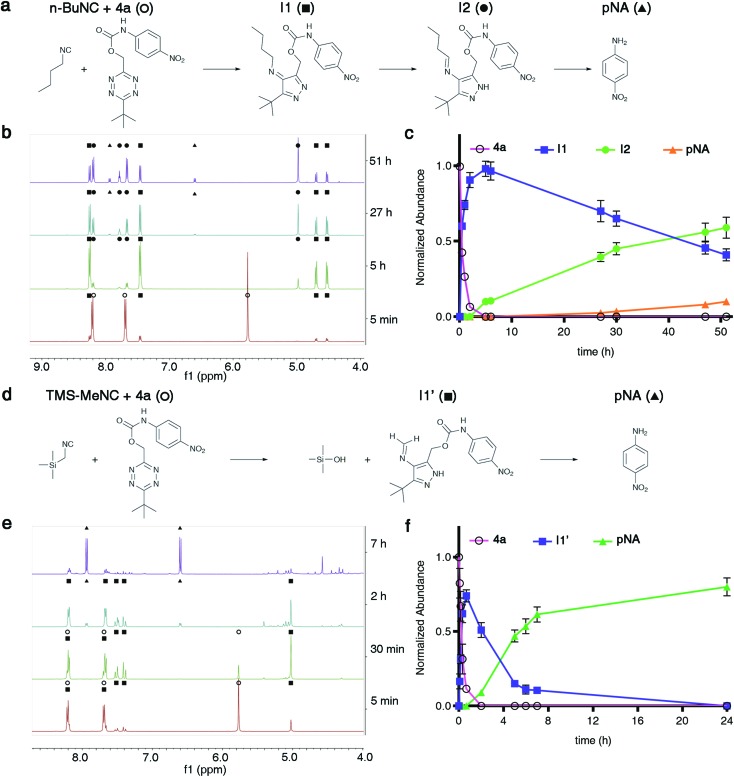
^1^H-NMR analysis of reactions between isonitriles *n*-BuNC or TMS-MeNC and **4a** to liberate pNA. (a and d) Proposed intermediates in the reaction between *n*-BuNC (a) or TMS-MeNC (d) and **4a** leading to the release of pNA. (b and e) Time-dependent ^1^H-NMR of the reaction progress between *n*-BuNC (b) or TMS-MeNC (e) and **4a** (c(**4a**) = 6 mM, c(R–NC) = 15 mM, DMSO-d_6_ : D_2_O (9 : 1, v/v), *T* = 25 °C, expanded spectra in ESI). (c and f) Normalized amount of starting material (**4a**), subsequent intermediates, and pNA formed as a function of time.

Having established that isonitriles remove TzMe-moieties from phenols and amines (Fig. 2), we aimed to gain a mechanistic understanding of the reaction. Several reaction pathways are conceivable. Carbamate release could in principle occur by heterolytic cleavage of the benzylic C–O bond or by a cyclization step involving the attack of a nucleophilic intermediate on the carbonyl (Fig. S11†). To elucidate the reaction pathway, we performed a time-dependent NMR experiment between **4a** and *n*-BuNC (DMSO-d_6_ : D_2_O (9 : 1, v/v) at *T* = 25 °C; Fig. 4a–c and S12, S13†). At a lower aqueous content and *T* = 25 °C, as opposed to the higher water content and *T* = 37 °C we preformed kinetics studies with previously (Fig. 2d), we expected slower reaction kinetics to allow for rigorous examination of the intermediates formed along the reaction pathway. As determined by ^1^H NMR, the formation of one equivalent of the 4*H*-pyrazole intermediate (**I1**) paralleled the disappearance of **4a** in the reaction with *n*-BuNC (Fig. 4b and c). **I1** subsequently tautomerized to the 1*H*-pyrazole intermediate (**I2**) that gradually released pNA (Fig. 4b and c). The triplet peak of **I2** centered at 7.78 ppm with a normalized integration value corresponding to one proton is characteristic for the N=CH–CH_2_ proton present in the postulated structure of **I2**. In reactions between *n*-BuNC and di-methyl-tetrazine (Fig. S14†) or di-*tert*-butyl-tetrazine (Fig. S16†), the same characteristic triplet peak at ∼7.8 ppm was present (Fig. S15 and S17†), which indicated that the signal originated from the *n*-BuNC portion providing additional support for the structural assignment of **I2**. The observed reaction cascade mirrored the predicted mechanism (Fig. 1).^35^,^36^ Interestingly, the ^1^H NMR signals of pNA (d, 2H, 6.60 ppm; d, 2H, 7.94 ppm) emerged before those of the aldehyde (s, 1H, 9.64 ppm). It therefore appears that the elimination step can occur from the imine intermediate **I2**.

We proceeded to study the reaction between TMS-MeNC and **4a** by NMR (DMSO-d_6_ : D_2_O (9 : 1, v/v), *T* = 25 °C; Fig. 4d–f and S18, S19†). TMS-MeNC was completely stable for >7 days under the experimental conditions (data not shown) ruling out the possibility that a decomposition product caused the fast release. Time-dependent ^1^H NMR spectra revealed a single intermediate (**I1′**; Fig. 4e). **I1′** exhibited a strong coupling peak pattern centered at 7.45 ppm with a normalized integration value corresponding to two protons that was absent from **I1** and **I2** (Fig. 4b). Repeating the experiment in DMSO-d_6_, without the addition of the 10% D_2_O, led to a peak at 5.28 ppm corresponding to one proton, which could not be assigned to the pyrazole species (Fig. S20 and S21†). To further examine the transformation, we performed the reaction between TMS-MeNC and di-*tert*-butyl-tetrazine in DMSO-d_6_ at *T* = 25 °C (Fig. S22–S27†). This reaction provided an adduct with the same strong coupling pattern with a normalized integration value corresponding to two protons and this species persisted for days in DMSO-d_6_, making it possible to thoroughly analyze its structure by various NMR experiments.

The strongly coupled protons that centered at 7.60 ppm in the ^1^H spectrum (Fig. S23†) correlated in the gCOSY spectrum (Fig. S24†) and according to gHSQC analysis, were bonded to the same carbon with a chemical shift of 159.7 ppm (Fig. S25 and S26†). Furthermore, these protons showed a multi-bond correlation with one of the aromatic ring carbons in gHMBC (130.9 ppm; Fig. S27†). The spectroscopic data is consistent with the formation of a methanimine intermediate, which would indicate cleavage of the C–Si bond (Fig. 4d). In agreement, trimethylsilanol was detected in the ^1^H NMR spectrum (s, 1H, 5.28 ppm; s, 9H, 0.01 ppm; Fig. S23†). The peak corresponding to the trimethylsilyl protons in the reaction of **4a** with TMS-MeNC remained unaffected as the reaction proceeded to generate several unidentified side products, further corroborating the formation of trimethylsilanol (Fig. S19†).

**Fig. 5:**
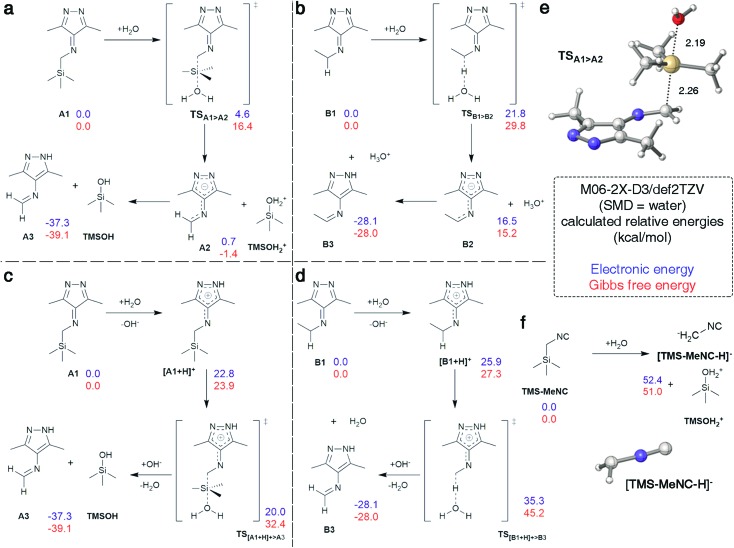
Investigated mechanistic pathways. (a) Cleavage of TMS from **A1** by water with subsequent protonation. (b) Tautomerization of **B1** induced by abstraction of a proton by water. (c) Cleavage of TMS from **A1** by protonation followed by abstraction by water. (d) Tautomerization of **B1** induced by protonation. (e) Predicted S_N_2 transition state TS_A1>A2_. (f) Cleavage of TMS from TMS-MeNC by water.

Cleavage of the C–Si bond under these conditions is surprising as documented cases required harsher conditions.^48^ We analyzed this reaction step using density functional theory (DFT) calculations. The analysis was conducted in Gaussian 09 using M06-2X-D3/def2TZV^49^–^51^ in water (SMD).^52^ 3,6-Di-methyl-1,2,4,5-tetrazine derived intermediates **A1** and **B1** were used as model substances (Fig. 5) with water as the initial nucleophile or proton source in all pathways to reflect the neutral experimental conditions. The S_N_2 reaction between **A1** and water was identified as the minimum energy pathway for the formation of imine **A3** going through a highly stabilized anion **A2** making this structure an excellent leaving group, allowing for low barriers even with weak nucleophiles such as water (Fig. 5a and e). The barrier was calculated to be 16.4 kcal mol^–1^ and this pathway is therefore in accordance with the fast reaction observed experimentally (Fig. 2d). In contrast, the deprotonation of **B1** by water to initiate the tautomerization had a calculated barrier of 29.8 kcal mol^–1^ with the resulting intermediate **B2** being 15 kcal mol^–1^ higher in energy than the reactants (Fig. 5b). While the omission of tunneling effects may overestimate barriers for proton transfers calculated with a classical treatment of the nucleus, it is plausible to assume that the barrier is above the 16.4 kcal mol^–1^ calculated for the **A1** > **A2** transformation, given that the intermediate **B2** is already at +15.2 kcal mol^–1^. This computational prediction agrees with the experimental observation that the tautomerization in case of reactions with *n*-BuNC proceed significantly slower than the cleavage of TMS (Fig. 4). Analogous pathways involving OH^–^ instead of water showed the same trend with overall lower barriers (Fig. S30†).

Alternative pathways that involve protonation of **A1** and **B1** followed by transfer of TMS^+^ to water, or deprotonation, respectively, were also explored (Fig. 5c and d). Protonation of **A1** or **B1** was disfavored by 23.9 and 27.3 kcal mol^–1^, respectively. The barriers for the following abstraction of TMS^+^ or H^+^ are lowered considerably compared to the pathways described above. However, this pathway also favors removal of TMS^+^ from protonated **A1** over proton abstraction from protonated **B1** in accordance to experimental results.

In addition, stability of TMS-MeNC against nucleophilic attack of water was investigated computationally. While the transition state structure could not be located, the transformation is disfavored by over 51.0 kcal mol^–1^ because of the inability of the adjacent isonitrile group to stabilize a carbanion at the α carbon, leading to poor leaving group qualities. The geometry of the anion shows a tetrahedral center at the α methyl group, consistent with isolation of the negative charge on this center without any stabilization by the adjacent π-systems (Fig. 5f). The high barrier and thermodynamically disfavored nature of this transition corroborate the observed high stability of TMS-MeNC in aqueous solution.

We further examined the mechanistic steps of phenol release triggered by *n*-BuNC. The photospectrometric studies had revealed a puzzling discrepancy in the rate of carbamate *versus* phenol elimination induced by *n*-BuNC (Fig. 2). To obtain mechanistic insight into this discrepancy, we analyzed the reaction of **4b′** and *n*-BuNC (DMSO-d_6_ : D_2_O (9 : 1), *T* = 25 °C) by ^1^H NMR (Fig. S28 and S29†). In this experiment, the formation of the corresponding 4*H*-pyrazole species, which was noticeable in the reaction between *n*-BuNC and **4a** (**I1**; Fig. 4a), was unobservable. The tautomerization step to the aromatic 1*H*-pyrazole following the bimolecular cycloaddition step therefore seems to proceed substantially more rapidly for the phenol than for the carbamate.

The remaining gap in the mechanism is the actual elimination step. We observed a striking dependence of pNA release on the presence of water (Fig. S31†). In anhydrous DMSO, pNA release was quasi-absent; however, traces of water induced the rapid release of pNA. Water therefore participates in the release step. Several possible release pathways are conceivable. One possible mechanism could be elimination of the benzylic leaving group induced by deprotonation of the pyrazole. Alternatively, water could attack the imine with concerted electron migrations and elimination of the leaving group (Fig. S32†).

In summary, through a combination of DFT analysis and empirical studies, it was possible to establish and validate a likely reaction mechanism. The reaction cascade largely followed the predicted steps of cycloaddition, N_2_ expulsion, tautomerization, and elimination, with the unexpected cleavage of the C–Si bond in case of TMS-MeNC.

### Demonstration of TzMe-deprotection on biomacromolecules, in cells, and in living vertebrates

**Fig. 6:**
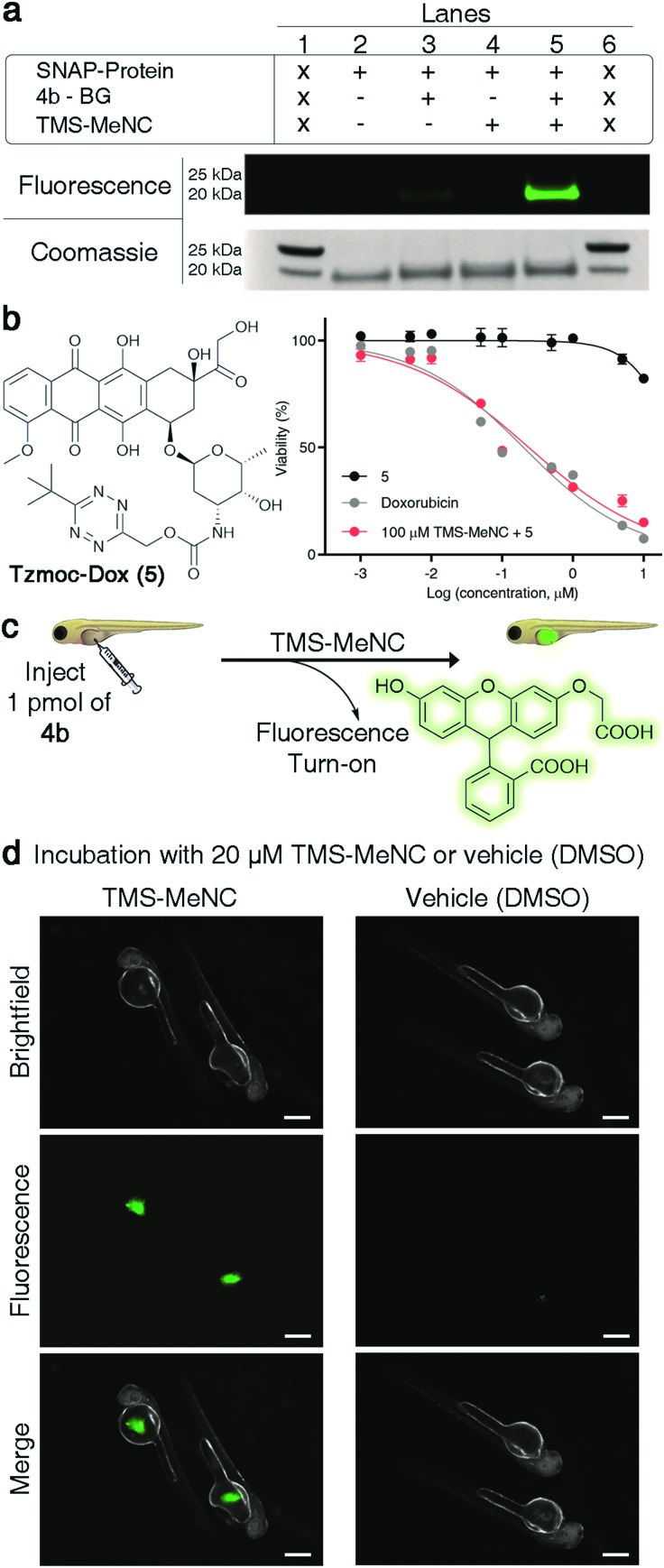
TMS-MeNC mediated removal of TzMe-modified molecules on proteins, in the presence of cells, and in zebrafish embryos. (a) In-gel analysis of the fluorescent turn-on signal on SNAP protein labelled with **4b-BG** (10 μM) and subsequent deprotection of the TzMe group with TMS-MeNC (100 μM); lanes 1 and 6 contain the protein ladder (for an expanded view of the fluorescence and Coomassie-stained gel and mass spectroscopy verification, see Fig. S34–S37 in the ESI†). (b) Structure of Tzmoc-caged doxorubicin prodrug (5) and dose–response curves for cytotoxicity studies with A549 cells after 72 h. (c) Cartoon representation of experiment to demonstrate the release of *O*-carboxymethyl fluorescein and fluorescence turn-on upon incubation with TMS-MeNC. (d) Visualization of the fluorescence signal in live zebrafish (scale bar = 200 μm) after a 2 h incubation with either 20 μM TMS-MeNC or DMSO.

Many potential applications of the presented chemistry would require it to be compatible with living systems. We aimed to show that the developed chemistry can be performed under physiologically relevant conditions. We first tested whether it is possible to conjugate a TzMe-modified probe to a protein and unmask it with TMS-MeNC. For proof of principle, we used the SNAP-tag system^53^ for protein labeling. We synthesized an O^6^-benzylguanine derivative of **4b** (**4b-BG**, Fig. S33†) and labelled a purified SNAP protein (New England Biolabs) with it. The SNAP protein-**4b-BG** conjugate was exposed to TMS-MeNC (100 μM) and after 2 h, the uncaging of the fluorophore was examined by protein gel analysis (Fig. 6a and S34†). The protein incubated with TMS-MeNC was visible by a strong in-gel fluorescence signal, whereas the fluorescence signal for controls was low. A 11-fold increase in the fluorescence signal was measured upon treatment with TMS-MeNC relative to untreated controls (Fig. S34†). Mass-spectrometry experiments confirmed the labeling of the SNAP tag to afford the SNAP protein-**4b-BG** conjugate and the efficient removal of the TzMe group by TMS-MeNC (>80%) in the given time window (Fig. S35–S37†). These results demonstrated that it is possible to conjugate TzMe-modified groups to biomacromolecules and to actuate them by treatment with isonitriles thereafter.

Next, we tested our chemistry with cultured cells. Restoration of the cytotoxicity of a Tzmoc-caged doxorubicin prodrug (Tzmoc-Dox (**5**), Fig. 6b; synthesis described in the ESI†) was tested with cultured A549 lung adenocarcinoma cells. In the presence of TMS-MeNC (100 μM) the prodrug was as toxic (EC_50_ = 0.239 ± 0.014 μM; Fig. 6b) as genuine doxorubicin (EC_50_ = 0.202 ± 0.025 μM; Fig. 6b). Tzmoc-Dox alone showed almost no toxicity below 10 μM, confirming the traceless activation of the doxorubicin prodrug. Exposure to 100 μM TMS-MeNC for 72 h caused no cell toxicity (Table S3†).

To demonstrate that TMS-MeNC can activate TzMe-modified molecules *in vivo*, we performed experiments in zebrafish embryos (Fig. 6c). The non-fluorescent TzMe-modified fluorescein derivative **4b** was injected into the yolk sac of zebrafish embryos. The fish were then incubated in either medium containing 20 μM TMS-MeNC or only its vehicle (DMSO) for 2 hours. Subsequently, the fish were washed, and the fluorescence turn-on signal analyzed by fluorescence microscopy (Fig. 6d). Strong green fluorescence staining localized to the yolk sac was observed for **4b**-injected fish incubated with TMS-MeNC, whereas **4b**-injected control fish treated with vehicle (DMSO) exhibited low fluorescence (Fig. 6d). A 3.8-fold higher fluorescence signal was measured in TMS-MeNC treated fish relative to untreated controls (Fig. S38†; *p*-value ≤ 0.001). Exposure to 20 μM TMS-MeNC for the duration of the study caused no developmental issues to the zebrafish embryos. These experiments establish that the reaction of TMS-MeNC and TzMe-groups is suitable for experiments with biomolecules and living organisms.

### Dual-release by combining TzMe- with ICPr-modified molecules

**Fig. 7:**
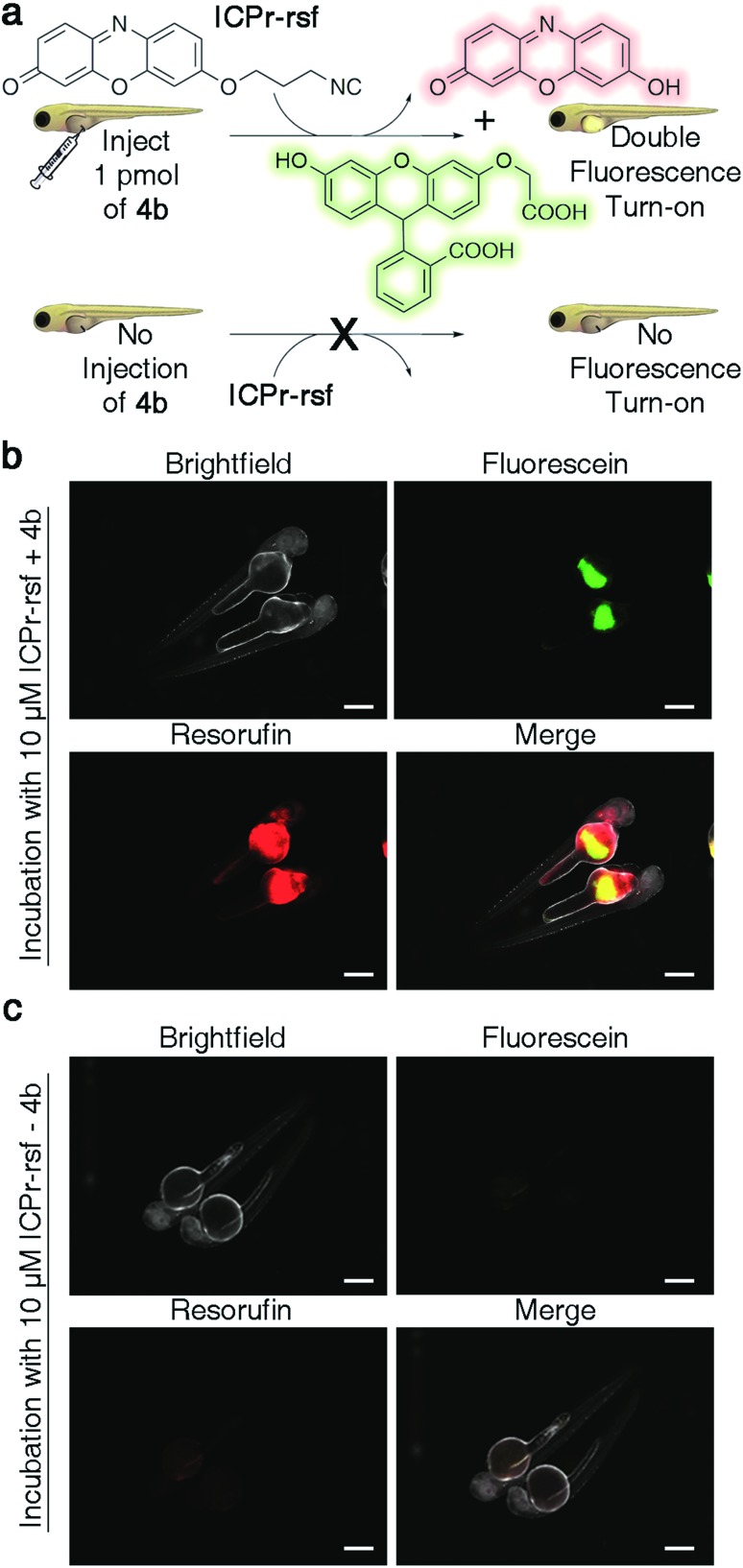
Dual release of two orthogonal fluorophores from ICPr- and TzMe-caged dyes in live zebrafish. (a) Cartoon representation of experiment demonstrating the dual release and fluorescence turn-on of *O*-carboxymethyl fluorescein and resorufin upon reaction of **4b** and **ICPr-rsf**, and the corresponding control experiment with non-injected fish. (b) Visualization of fluorescein and resorufin fluorescence signal in live zebrafish (scale bar = 200 μm) injected with **4b** after a 2 h incubation with 10 μM **ICPr-rsf**. (c) Visualization of fluorescein and resorufin fluorescence signal in non-**4b** injected control live zebrafish (scale bar = 200 μm) after a 2 h incubation with 10 μM **ICPr-rsf**.

There is considerable interest in developing reaction schemes that allow for the release of two molecules simultaneously *in vivo*.^24^,^39^ We rationalized that combining the TzMe-release chemistry with our previously disclosed 3-isocyanopropyl (ICPr) chemistry,^37^ would liberate two independent payloads. Dual release was first tested *in vitro*. The TzMe-caged fluorescein dye **4b** was incubated with **ICPr-rsf**, an ICPr-caged resorufin probe^37^ (c(**4b**) = 0.5 mM, c(**ICPr-rsf**) = 1 mM, DMSO : PBS pH 7.4 (4 : 1), *T* = 37 °C, *λ* = 480 nm) and concurrent fluorophore release was analyzed by HPLC. The traceless release of both *O*-carboxymethyl fluorescein and resorufin was observed (Fig. S39†). Dual release from combinations of TzMe/ICPr-reagents was then tested in vertebrates. Zebrafish embryos were either injected with **4b** or left untreated (Fig. 7a). The fish were then incubated in media containing 10 μM **ICPr-rsf** for 2 hours, washed, and fluorescence turn-on signals analyzed by fluorescence microscopy (Fig. 7b and c). Strong emission signals were detected in the yolk sac in both green and red fluorescence channels for fish injected with **4b**. (Resorufin: *p*-value ≤ 0.0001; fluorescein: *p*-value ≤ 0.0001; Fig. S38†). Neither **4b** (Fig. 6d) nor **ICPr-rsf** (Fig. 7c) alone produced obvious fluorescence signal confirming that it was the reaction between the isonitrile and the tetrazine that led to the concurrent release of the two fluorophores. While it is acknowledged that precisely controlling the injected probe volume into the yolk sac is challenging, the 60-fold higher resorufin (*p*-value ≤ 0.0001) and 3.6-fold higher fluorescein signal (*p*-value ≤ 0.0001) in zebrafish treated with both reactive species relative to controls indicate unmasking of a considerable fraction of the fluorophores (Fig. S38†). Conclusively, combining TzMe- and ICPr-reactants can simultaneously liberate pairs of molecules of interest.

## Conclusions

In summary, the study introduces TzMe-substituents as protecting groups that are removed under physiological conditions by isonitriles. In a series of steps, we demonstrated that TzMe-groups could reversibly cage amines and phenols with near-quantitative release yields. Fast elimination occurred for phenols and for amines could be achieved by the addition of HSA or the use of TMS-MeNC. NMR and DFT studies revealed that the reaction followed the expected mechanism of cycloaddition and tautomerization to the imine. Unexpectedly, the elimination step could occur from the imine intermediate and this step involved the need for water. Furthermore, it was observed that in reactions with TMS-MeNC, the C–Si bond dissociated to generate a methanimine intermediate. TzMe-caged fluorophore release on a protein, cytotoxicity experiments with a doxorubicin-prodrug and cultured cells and fluorophore release in zebrafish embryos demonstrated the potential utility of the reaction in chemical biology and in the context of living systems. It is worth noting that the reaction with TMS-MeNC generates formaldehyde as side product. However, endogenous levels of formaldehyde (50–100 μM in serum and 200–500 μM in cells)^54^ exceed the levels that would be released in most foreseeable applications. Furthermore, metabolic pathways counteract formaldehyde toxicity primarily mediated by glutathione^55^ and formaldehyde is diverted to the one-carbon metabolism.^56^ The toxicity of aldehyde side products of other isonitriles (*e.g.* butanal for *n*BuNC) is typically even lower.

Combining TzMe-with ICPr-molecules allowed for the first time the unmasking of two pro-fluorophores by a single bioorthogonal reaction. Multiple synergistic drug combinations would benefit from simultaneous and controlled delivery. Achieving release in a single reaction is important because controlling the delivery and stability of four individual reactants required for two reactions occurring in parallel would be challenging. This versatile protecting group chemistry constitutes a valuable addition to the dissociative bioorthogonal chemistry and synthetic methodology toolbox with potential utility for a broad range of applications. In addition to uses in drug delivery and controlling biomolecules, it may also be valuable as a protecting group for the synthesis of sensitive molecules allowing for late-stage deprotection under extremely mild conditions.

## Conflicts of interest

There are no conflicts to declare.

## Supplementary Material

Supplementary information
