# Supplementary material for: Isonitrile-responsive and bioorthogonally removable tetrazine protecting groups
Source: Chem Sci. 2019 Nov 5;11(1):169–79. doi: 10.1039/c9sc04649f (PMC7012038; doi:10.1039/c9sc04649f)
Supplement: Supplementary file 1 [file SC-011-C9SC04649F-s001.pdf]

Electronic Supplementary Material (ESI) for Chemical Science.
This journal is © The Royal Society of Chemistry 2019

# Isonitrile-responsive and Bioorthogonally Removable Tetrazine Protecting Groups

## Supporting Information

Julian Tu,[a] Dennis Svatunek,[b] Saba Parvez,[c] Hannah J. Eckvahl,[b] Minghao Xu,[a, d] Randall T. Peterson,[c] K. N. Houk,[b] and Raphael M. Franzini*,[a]

[a]Department of Medicinal Chemistry, College of Pharmacy, University of Utah, Salt Lake City, 84112, USA

[b]Department of Chemistry and Biochemistry, University of California, Los Angeles, California 90095, USA

[c]Department of Pharmacology and Toxicology, College of Pharmacy, University of Utah, Salt Lake City, 84112, USA

[d](Current address) School of Chemistry and Biochemistry, Georgia Institute of Technology, Atlanta, Georgia 30332, United States

*E-mail: Raphael.franzini@utah.edu

## Table of Contents


| | |
|---|---|
| **Materials and Instrumentation** | S3 |
| **Synthetic Procedures** | S4 - S15 |
| **Photospectrometric Analysis of Bimolecular Reaction** | S16 |
| **Human Serum Albumin vs. General Base Catalysis** | S17 |
| **Photospectrometric Analysis of Uncaging Step** | S18 - S22 |
| **Human Liver Microsome Stability Assay** | S23 - S24 |
| **Serum Stability of TzMe Probes** | S25 |
| **Effects of Structural Modifications on TzMe probes** | S26 |
| **Removal of TzMe Groups by Alternative Dienophiles** | S27 |
| **NMR Analyses** | S28 - 38 |
| **Computational Analyses** | S39 - 41 |
| **Dependence of the Release Step on Water** | S42 |
| **SNAP-protein In-Gel Fluorescence and Mass-spectroscopy** | S43 - 46 |
| **Cytotoxicity Assay** | S47 |
| **Zebrafish Imaging** | S48 - S49 |
| **Dual Release of TzMe- and ICPr-caged Molecules** | S50 |
| **References** | S51 |
| **NMR Spectra of Synthesized Compounds** | S52 - 68 |
| **Cartesian Coordinates** | S69 - 73 |

## Materials and Instrumentation

All chemical and biological reagents/solvents were obtained from commercial sources (Sigma-Aldrich, Alfa-Aesar, Combi-Blocks, Oakwood Chemical, Enamine, Acros-Organic, ThermoFisher, Invitrogen, New England BioLabs) and used without further purification. Thin-layer chromatography (TLC) analysis was carried out to monitor the progress of reactions. Purification of compounds was performed by column chromatography with silica gel 300-400 mesh. $^1$H NMR, $^{13}$C NMR, and two-dimensional spectra were recorded on a Varian Mercury-400, Varian Inova-500, or Varian Inova-600 MHz with HCN cryogenic probe spectrometer with chemical shifts expressed as ppm (in $CDCl_3$, MeOD-$d_4$, $D_2O$ or DMSO-$d_6$) using tetramethylsilane as an internal standard. High-resolution mass spectra were measured by the University of California, Riverside Chemistry Mass Spectrometry Facility or by the Mass Spectrometry and Proteomics Core Facility at the University of Utah, with mass spectrometry equipment obtained through a Shared Instrumentation Grant 1 S10 OD018210 01A1 for the University of Utah. Analytical or preparative HPLCs were performed on a Dionex Ultimate3000 equipped with an autosampler, diode array detector and robotic fraction collector (Dionex Thermo Scientific, USA) using a Gemini C18 column (5 μm, 250×10 mm, Phenomenex, USA) or LUNA C18 column (5 μm, 150×2.0 mm, Phenomenex, USA). UHPLC and low-resolution mass was performed on an ACQUITY Arc equipped with a sample manager, quaternary solvent manager, column heater, PDA detector, and QDA detector (Waters, USA) using a UPLC BEH C18 column (1.7 μm, 50×2.1 mm, Waters, USA). UV and fluorescence photospectrometic measurements were performed with a SpectraMax M2 (Molecular Devices, USA) in 96-well plates. Protein gel images were obtained with a GelDoc EZ Gel documentation system (Bio-Rad, USA). Zebrafish imaging was performed with a Zeiss SteREO Discovery.V8 microscope (Zeiss, Jena, Germany) fitted with a PentaFluor S 120 vertical illuminator and coupled with an Xcite Series 120PC light source.

# Synthetic Procedures

## General procedure A:

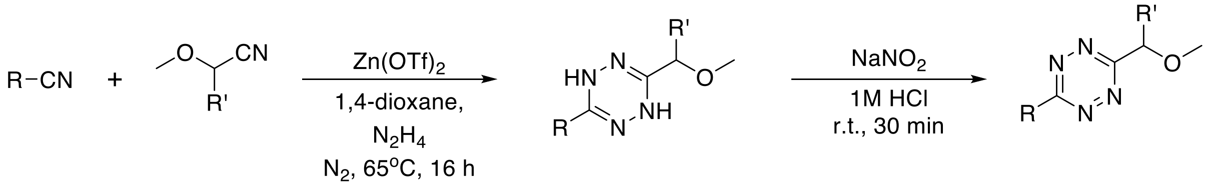


To a dry two-neck flask equipped with a stir bar was added the methoxyacetonitrile derivative (1 eq.) and the second nitrile (3 eq.) followed by zinc triflate (0.05 eq.). The flask was sealed and put under an atmosphere of nitrogen. To the stirring solution was added dropwise anhydrous 1,4-dioxane [5 M] followed by anhydrous hydrazine (5 eq.) at room temperature. Upon completion of addition, the reaction mixture was stirred at 65°C for 16 h before being cooled to room temperature. Sodium nitrite (10 eq.) in water (20% (w/v)) was added slowly to the solution and cooled to 0°C before a slow addition of 1M aq. HCl during which the solution turned bright red in color and gas evolved. Addition of 1M aq. HCl was continued until gas evolution ceased and ~pH 3 was reached. (Caution: toxic nitrogen oxide gas is formed). The resulting solution was returned to room temperature and extracted with DCM until the organic phase was nearly devoid of pink color. The combined organic layers were washed with brine, dried with $MgSO_4$, filtered, and concentrated under reduced pressure for purification by silica column chromatography.

## General Procedure B:

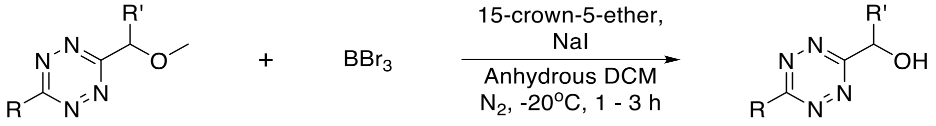


To an oven-dried two-neck flask equipped with a stir bar was added the methoxy protected tetrazine in anhydrous DCM [0.5 M] followed by a solution of 15-crown-5-ether [0.3 M] in sodium iodide saturated anhydrous DCM. The flask was sealed, cooled to -20°C in a salt/ice bath, and put under an atmosphere of nitrogen. To this stirring solution was added $BBr_3$ (1.0 M solution in DCM, 3 eq.) dropwise over 10 minutes resulting in an orange solution. The temperature was maintained at -20°C and stirred for the specified time before being quenched

by the dropwise addition of the reaction mixture to a stirring solution of ice-cold water upon which the organic layer returned to a pink color. The aqueous layer was extracted with DCM (2 x 50 mL) and the combined organic layers were washed with brine, dried with $MgSO_4$, filtered, and concentrated under reduced pressure for purification by silica column chromatography.

**General Procedure C:**

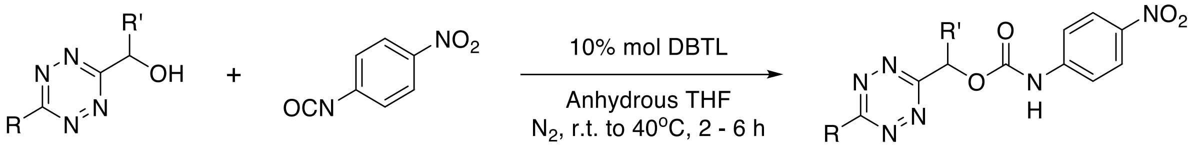


To an oven-dried two-neck flask equipped with a stir bar was added the hydroxy tetrazine derivative in anhydrous THF [0.05 M], dibutyltin dilaurate (DBTL, 10% mol), and 1-isocyanato-4-nitrobenzene (2 eq.). The reaction flask was sealed under an atmosphere of nitrogen, warmed to 40°C, and stirred for the specified time before allowing the reaction mixture to cool to room temperature. The cooled reaction mixture was concentrated under reduced pressure and the resulting residue was re-dissolved in DCM, washed with water (2 x 25 mL) and brine (1 x 20 mL), dried with $MgSO_4$, filtered, and concentrated under reduced pressure for purification by silica column chromatography.

*3-(tert-butyl)-6-(methoxymethyl)-1,2,4,5-tetrazine*

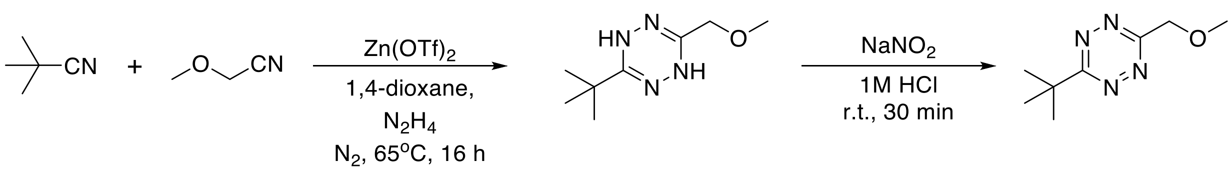


From the corresponding nitriles (1 eq. = 2.13 g), the compound was synthesized following general procedure A and purified by silica column chromatography (DCM, $R_f = 0.35$) to afford the desired compound as a dark purple oil (1.60 g, 30%). $^1H$ NMR (400 MHz, $CDCl_3$) δ 5.04 (s, 2H), 3.61 (s, 3H), 1.58 (s, 9H). $^{13}C$ NMR (100 MHz, $CDCl_3$) δ 176.73, 165.67, 77.31, 77.00, 76.68, 72.20, 59.69, 38.08, 29.13. HRMS (ESI): calcd. for $C_8H_{14}N_4O$ $[M+H]^+$ 183.1240, found 183.1240.

*2-methoxypropanenitrile*

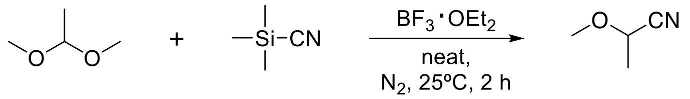


The desired compound was prepared following a modified literature procedure.[1] In brief, to an oven-dried two-neck flask equipped with a stir bar sealed under an atmosphere of nitrogen was added consecutively, 1,1-dimethoxyethane (3.17 mL, 30 mmol), trimethylsilanecarbonitrile (3.80 mL, 30 mmol), and boron trifluoride diethyl etherate (188 µL, 1.5 mmol) at room temperature. The solution was stirred for 2 hours before being quenched by the addition of saturated $NaHCO_3$ (aq.) solution (10 mL). The mixture was diluted in DCM (100 mL), washed with sat. $NaHCO_3$ (1 x 150 mL) and brine (1 x 20 mL), dried with $MgSO_4$, filtered, and purified by distillation to afford the desired compound as a colorless oil (1.54 g, 60%). $^1$H NMR (400 MHz, $CDCl_3$) δ 4.13 (q, J = 6.8 Hz, 1H), 3.46 (s, 3H), 1.53 (d, J = 6.8 Hz, 3H).

*3-(tert-butyl)-6-(1-methoxyethyl)-1,2,4,5-tetrazine*

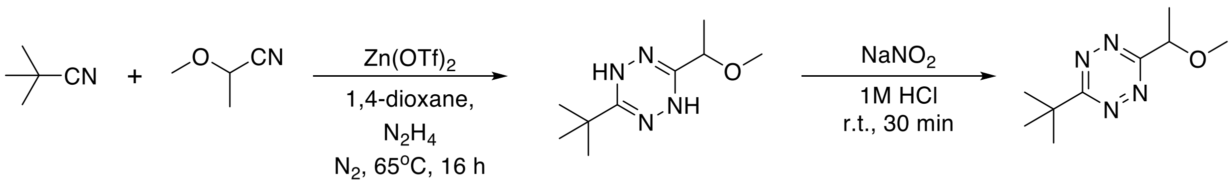


From the corresponding nitriles (1 eq. = 750 mg), the compound was synthesized following general procedure A and purified by silica column chromatography (DCM, $R_f$ = 0.3) to afford the desired compound as a dark pink oil (300 mg, 17%). $^1$H NMR (400 MHz, $CDCl_3$) δ 4.97 (q, J = 6.6 Hz, 1H), 3.45 (s, 3H), 1.73 (d, J = 6.6 Hz, 3H), 1.59 (s, 9H). $^{13}$C NMR (100 MHz, $CDCl_3$) δ 176.81, 168.60, 77.67, 77.31, 76.99, 76.67, 57.63, 38.06, 29.13, 19.98. HRMS (ESI): calcd. for $C_9H_{16}N_4O$ $[M+H]^+$ 197.1397, found 197.1397.

*2-methoxy-2-phenylacetonitrile*

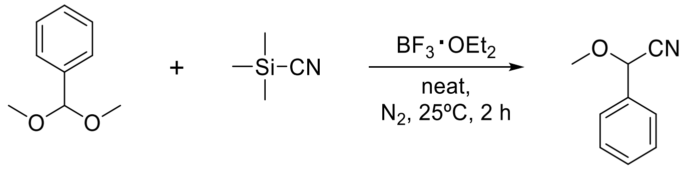

The desired compound was prepared following a modified literature procedure.[1] In brief, to an oven-dried two-neck flask equipped with a stir bar sealed under an atmosphere of nitrogen was added consecutively, (dimethoxymethyl)benzene (1.02 mL, 10 mmol), trimethylsilanecarbonitrile (1.27 mL, 10 mmol), and boron trifluoride diethyl etherate (63 μL, 0.5 mmol) at room temperature. The solution was stirred for 2 hours before being quenched by the addition of saturated $NaHCO_3$ (aq.) solution (10 mL). The mixture was diluted in DCM (50 mL), washed with sat. $NaHCO_3$ (1 x 50 mL) and brine (1 x 20 mL), dried with $MgSO_4$, filtered, and concentrated under reduced pressure for purification by silica column chromatography (Hex:EA, 10:1, v/v, $R_f$ = 0.3) to afford the desired product as a colorless oil (864 mg, 59%). $^1H$ NMR (400 MHz, $CDCl_3$) δ 7.55 – 7.37 (m, 5H), 5.20 (s, 1H), 3.53 (s, 3H).

*3-(tert-butyl)-6-(methoxy(phenyl)methyl)-1,2,4,5-tetrazine*

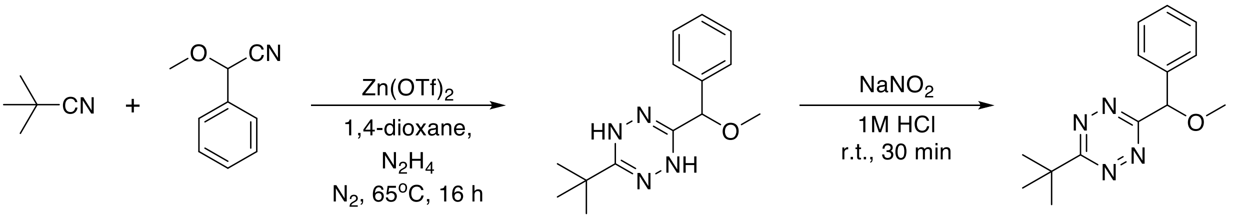


From the corresponding nitriles (1 eq. = 850 mg), the compound was synthesized following general procedure A and purified by silica column chromatography (DCM, $R_f$ = 0.4) to afford the desired compound as a pink solid (30 mg, 2%). $^1H$ NMR (400 MHz, $CDCl_3$) δ 7.65 (d, J = 7.2 Hz, 2H), 7.36 (m, 3H), 5.88 (s, 1H), 3.53 (s, 3H), 1.55 (s, 9H). $^{13}C$ NMR (100 MHz, $CDCl_3$) δ 176.54, 167.95, 137.50, 128.84, 128.82, 127.47, 83.92, 77.32, 77.00, 76.68, 57.69, 38.06, 29.11. HRMS (ESI): calcd. for $C_{14}H_{18}N_4O$ $[M+H]^+$ 259.1553, found 259.1549.

*3-(methoxymethyl)-6-phenyl-1,2,4,5-tetrazine*

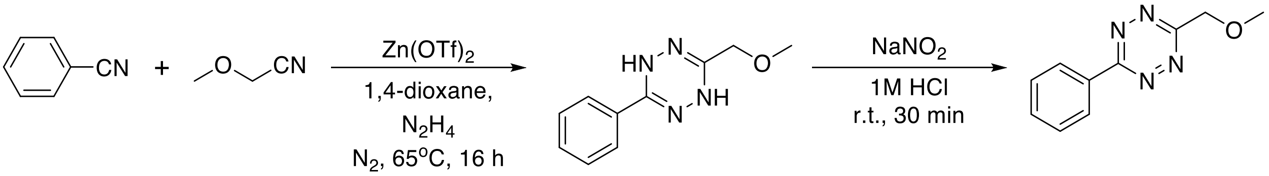


From the corresponding nitriles (1 eq. = 711 mg), the compound was synthesized following general procedure A and purified by silica column chromatography (Hex:DCM, 3:1, v/v, $R_f$ =

0.2) to afford the desired compound as a pink solid (403 mg, 20%). $^{1}$H NMR (400 MHz, DMSO-$d_6$) δ 8.49 (d, J = 7.0 Hz, 2H), 7.73 – 7.64 (m, 3H), 5.01 (s, 2H), 3.47 (s, 3H). $^{13}$C NMR (100 MHz, DMSO-$d_6$) δ 166.59, 164.57, 133.21, 132.16, 129.93, 128.18, 71.94, 59.08, 40.56, 40.36, 40.15, 39.94, 39.73, 39.52, 39.31.

*(6-(tert-butyl)-1,2,4,5-tetrazin-3-yl)methanol*

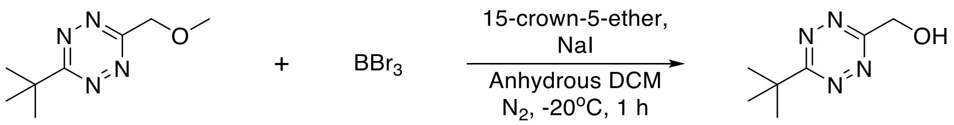


From the corresponding methoxy protected tetrazine (80 mg), the compound was synthesized following general procedure B and purified by silica column chromatography (DCM:MeOH, 200:1, v/v, $R_f$ = 0.2) to afford the desired compound as a dark purple oil (45 mg, 61%).
$^{1}$H NMR (400 MHz, $CDCl_3$) δ 5.28 (s, 2H), 3.15 (s, 1H), 1.59 (s, 9H). $^{13}$C NMR (100 MHz, $CDCl_3$) δ 177.08, 166.76, 77.31, 76.99, 76.68, 62.69, 38.12, 29.15. HRMS (ESI): calcd. for $C_7H_{12}N_4O$ $[M+H]^+$ 169.1084, found 169.1086.

*1-(6-(tert-butyl)-1,2,4,5-tetrazin-3-yl)ethan-1-ol*

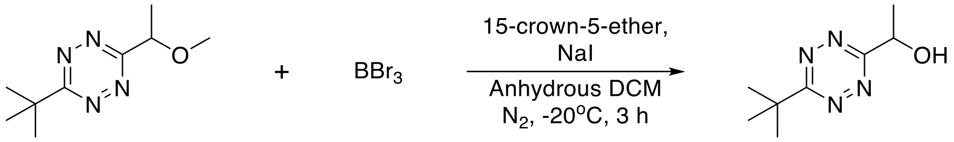


From the corresponding methoxy protected tetrazine (50 mg), the compound was synthesized following general procedure B and purified by silica column chromatography (DCM:MeOH, 50:1, v/v, $R_f$ = 0.3) to afford the desired compound as a dark purple oil (19 mg, 41%). $^{1}$H NMR (400 MHz, $CDCl_3$) δ 5.42 (s, 1H), 3.36 (s, 1H), 1.78 (d, J = 6.2 Hz, 3H), 1.60 (s, 9H). $^{13}$C NMR (100 MHz, $CDCl_3$) δ 176.86, 169.65, 77.30, 76.99, 76.67, 68.39, 38.09, 29.15, 22.70. HRMS (ESI): calcd. for $C_8H_{14}N_4O$ $[M+H]^+$ 183.1240, found 183.1240.

*(6-(tert-butyl)-1,2,4,5-tetrazin-3-yl)(phenyl)methanol*

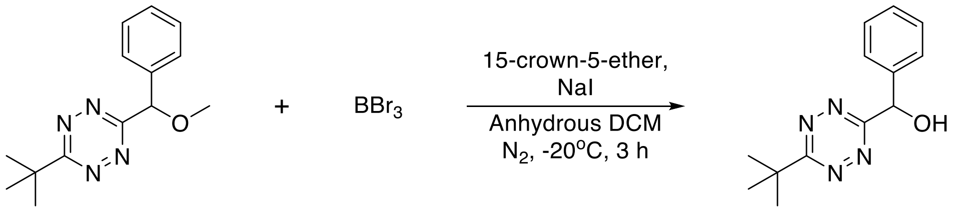


From the corresponding methoxy protected tetrazine (25 mg), the compound was synthesized following general procedure B and purified by silica column chromatography (DCM, $R_f = 0.2$) to afford the desired compound as a dark purple oil (10 mg, 42%). $^1H$ NMR (400 MHz, $CDCl_3$) δ 7.59 (d, J = 7.4 Hz, 2H), 7.45 – 7.29 (m, 3H), 6.37 (d, J = 6.8 Hz, 1H), 3.99 (d, J = 6.8 Hz, 1H), 1.57 (s, 9H). $^{13}C$ NMR (100 MHz, $CDCl_3$) δ 176.97, 168.42, 139.82, 128.87, 128.67, 126.60, 77.31, 76.99, 76.68, 74.25, 38.12, 29.11. HRMS (ESI): calcd. for $C_{13}H_{16}N_4O$ $[M+H]^+$ 245.1397, found 245.1391.

*(6-phenyl-1,2,4,5-tetrazin-3-yl)methanol*

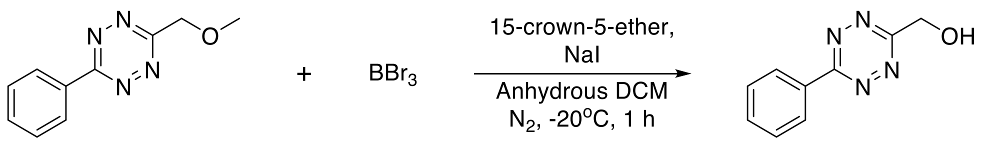


From the corresponding methoxy protected tetrazine (100 mg), the compound was synthesized following general procedure B and purified by silica column chromatography (Hex:DCM, 1:1, $R_f = 0.2$) to afford the desired compound as a pink solid (60 mg, 65%). $^1H$ NMR (400 MHz, $CDCl_3$) δ 8.61 (d, J = 7.0 Hz, 2H), 7.69 – 7.56 (m, 3H), 5.34 (s, 2H), 3.21 (s, 1H). $^{13}C$ NMR (100 MHz, $CDCl_3$) δ 167.30, 165.39, 133.01, 131.39, 129.35, 128.20, 77.32, 77.00, 76.69, 62.79. HRMS (ESI): calcd. for $C_9H_8N_4O$ $[M+H]^+$ 189.0771, found 189.0775.

*(6-(tert-butyl)-1,2,4,5-tetrazin-3-yl)methyl (4-nitrophenyl)carbamate (**4a**)*

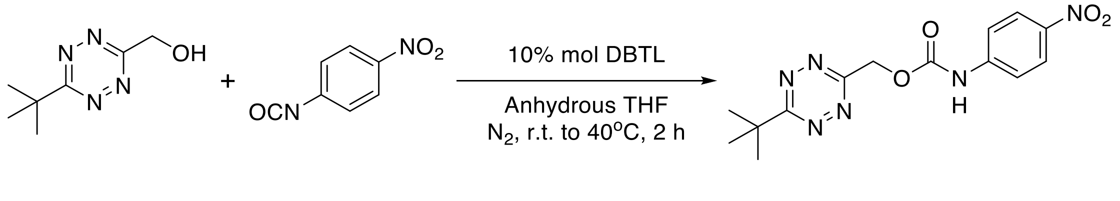


From the corresponding hydroxy tetrazine (10 mg), the compound was synthesized following general procedure C and purified by silica column chromatography (DCM:MeOH, 200:1, v/v,

$R_f$ = 0.3) to afford the desired compound as a pink solid (16 mg, 83%). $^1$H NMR (400 MHz, $CDCl_3$) δ 8.21 (d, J = 9.0 Hz, 2H), 7.58 (d, J = 9.0 Hz, 2H), 7.35 (s, 1H), 5.83 (s, 2H), 1.59 (s, 9H). $^{13}$C NMR (100 MHz, $CDCl_3$) δ 177.22, 164.26, 151.98, 143.42, 143.19, 125.21, 118.06, 77.31, 77.00, 76.68, 63.79, 38.20, 29.12. HRMS (ESI): calcd. for $C_{14}H_{16}N_6O_4$ $[M+H]^+$ 333.1306, found 333.1304.

*1-(6-(tert-butyl)-1,2,4,5-tetrazin-3-yl)ethyl (4-nitrophenyl)carbamate (**4c**)*

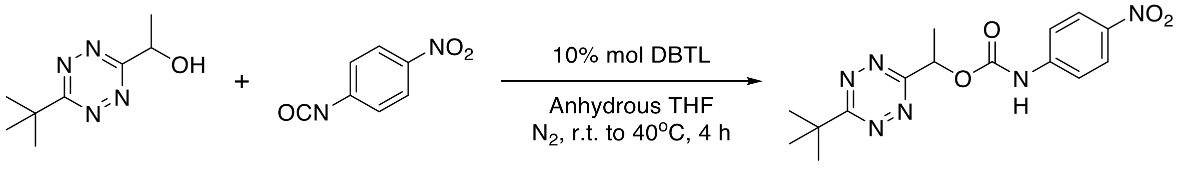


From the corresponding hydroxy tetrazine (14 mg), the compound was synthesized following general procedure C and purified by silica column chromatography (DCM:Hex, 5:1, v/v + 1% MeOH, $R_f$ = 0.3) to afford the desired compound as a pink solid (14 mg, 53%). $^1$H NMR (400 MHz, $CDCl_3$) δ 8.20 (d, J = 9.0 Hz, 2H), 7.54 (d, J = 9.0 Hz, 2H), 7.25 (s, 1H), 6.38 (q, J = 6.8 Hz, 1H), 1.90 (d, J = 6.8 Hz, 3H), 1.59 (s, 9H). $^{13}$C NMR (100 MHz, $CDCl_3$) δ 176.98, 167.58, 151.78, 143.29, 125.20, 117.95, 77.31, 76.99, 76.68, 71.47, 38.16, 29.13, 19.75. HRMS (ESI): calcd. for $C_{15}H_{18}N_6O_4$ $[M+H]^+$ 347.1462, found 347.1460.

*(6-(tert-butyl)-1,2,4,5-tetrazin-3-yl)(phenyl)methyl (4-nitrophenyl)carbamate (**4d**)*

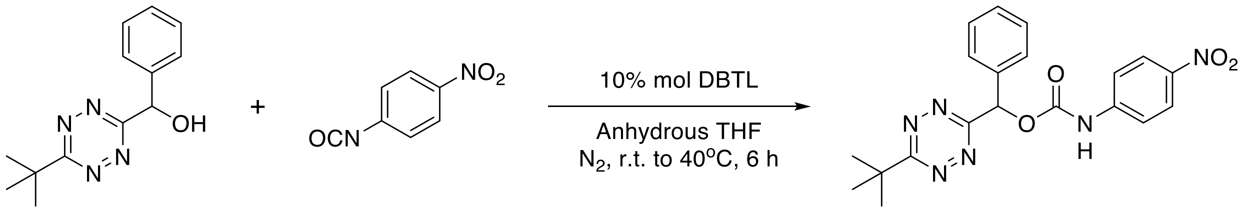


From the corresponding hydroxy tetrazine (5 mg), the compound was synthesized following general procedure C and purified by silica column chromatography (DCM:Hex, 50:1, v/v, $R_f$ = 0.4) to afford the desired compound as a pink solid (5 mg, 60%). $^1$H NMR (400 MHz, $CDCl_3$) δ 8.20 (d, J = 9.0 Hz, 2H), 7.66 (d, J = 6.8 Hz, 2H), 7.55 (d, J = 9.0 Hz, 2H), 7.45 – 7.38 (m, 3H), 7.37 (s, 1H), 7.25 (s, 1H), 1.57 (s, 9H). $^{13}$C NMR (100 MHz, $CDCl_3$) δ 129.65, 129.16, 127.80, 125.23, 118.05, 77.26, 77.16, 77.01, 76.75, 53.42, 38.20, 29.13. HRMS (ESI): calcd. for $C_{20}H_{20}N_6O_4$ $[M+H]^+$ 409.1619, found 409.1632.

*(6-phenyl-1,2,4,5-tetrazin-3-yl)methyl (4-nitrophenyl)carbamate (**4e**)*

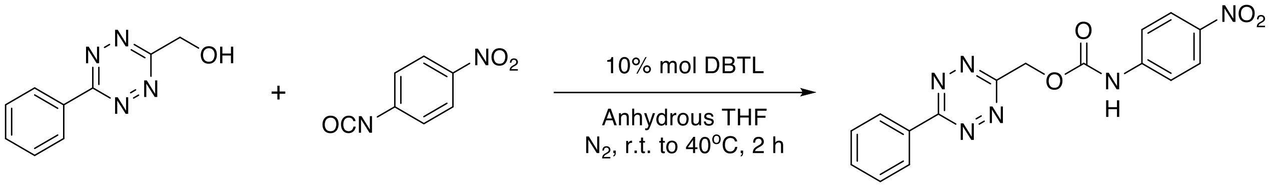


From the corresponding hydroxy tetrazine (10 mg), the compound was synthesized following general procedure C and purified by silica column chromatography (DCM:MeOH, 200:1, $R_f$ = 0.5) to afford the desired compound as a pink solid (15 mg, 82%). $^1$H NMR (400 MHz, $CDCl_3$) δ 8.62 (d, J = 7.0 Hz, 2H), 8.22 (d, J = 9.0 Hz, 2H), 7.91 – 7.50 (m, 5H), 7.30 (s, 1H), 5.87 (s, 2H). $^{13}$C NMR (100 MHz, $CDCl_3$) δ 165.33, 164.72, 151.96, 143.45, 143.18, 133.26, 131.17, 129.40, 128.31, 125.23, 118.07, 77.31, 76.99, 76.67, 63.94. HRMS (ESI): calcd. for $C_{16}H_{12}N_6O_4$ $[M+H]^+$ 353.0993, found 353.0997.

*3-(bromomethyl)-6-(tert-butyl)-1,2,4,5-tetrazine (**3**)*

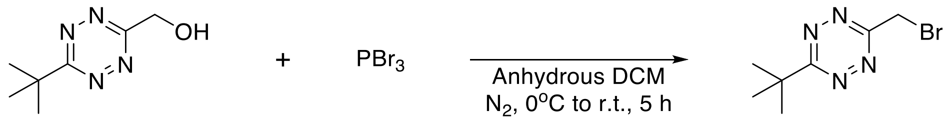


To an oven-dried two-neck flask equipped with a stir bar was added (6-(tert-butyl)-1,2,4,5-tetrazin-3-yl)methanol (50 mg, 0.3 mmol) in anhydrous DCM [0.2 M]. The flask was sealed, cooled to 0°C, and put under an atmosphere of nitrogen. To this stirring solution was added $PBr_3$ (20 µL, 0.2 mmol) dropwise over 10 minutes. Upon completion of addition, the ice bath was removed, and the mixture was allowed to stir for 5 hours at room temperature before being quenched by the dropwise addition of an ice-cold brine solution into the reaction mixture followed by additional DCM (25 mL). The layers were separated and the combined organic layers were washed with brine (3 x 15 mL), dried with $MgSO_4$, filtered, and concentrated under reduced pressure for purification by silica column chromatography (Hex:DCM, 1:1, v/v, $R_f$ = 0.45) to afford the desired product as a purple oil (60 mg, Caution: compound is volatile; 88%). This compound was made fresh and used immediately in the next step each time. $^1$H NMR (400 MHz, $CDCl_3$) δ 4.94 (s, 2H), 1.60 (s, 9H). $^{13}$C NMR (125 MHz, $CDCl_3$) δ 166.77, 77.25, 76.99, 76.74, 29.15, 27.57.

*7-((6-(tert-butyl)-1,2,4,5-tetrazin-3-yl)methoxy)-2H-chromen-2-one (**4b'**)*

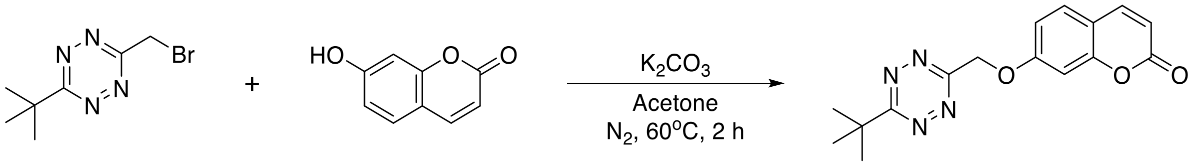


To an oven-dried two-neck flask equipped with a reflux condenser and a stir bar was solid umbelliferon (21 mg, 0.13 mmol) in acetone [0.2 M]. To the stirring solution was added solid anhydrous potassium carbonate (30 mg, 0.22 mmol) in a single portion followed by 3-(bromomethyl)-6-(tert-butyl)-1,2,4,5-tetrazine (20 mg, 0.09 mmol). The flask was sealed, put under an atmosphere of nitrogen, and the mixture heated to reflux. The reaction was stirred for 2 hours before allowing the reaction mixture to cool to room temperature. The cooled reaction mixture was concentrated under reduced pressure and the resulting residue was re-dissolved in DCM and filtered through celite. After removal of DCM, the crude material was re-dissolved in methanol for purification by semi-preparative reverse phase HPLC using a Gemini C18 column (5 μM, 250×10 mm, Phenomenex, USA) with a mobile phase consisting of A (0.1% TFA in water) and B (acetonitrile) running a gradient from 30% to 80% component B over a 10.5 minute timeframe (desired compound retention time 10 minutes) to afford the desired product as a pink solid (20 mg, 71%). $^1$H NMR (400 MHz, $CDCl_3$) δ 7.65 (d, J = 9.5 Hz, 1H), 7.43 (d, J = 8.5 Hz, 1H), 7.04 – 6.98 (m, 2H), 6.29 (d, J = 9.5 Hz, 1H), 5.72 (s, 2H), 1.61 (s, 9H). $^{13}$C NMR (125 MHz, $CDCl_3$) δ 164.44, 160.94, 155.71, 152.92, 143.13, 129.07, 113.95, 113.51, 112.92, 102.20, 77.25, 77.00, 76.75, 67.99, 29.15. HRMS (ESI): calcd. for $C_{16}H_{16}N_4O_3$ $[M+H]^+$ 313.1295, found 313.1298.

*tert-butyl2-((3'-((6-(tert-butyl)-1,2,4,5-tetrazin-3-yl)methoxy)-3-oxo-3H-spiro[isobenzofuran-1,9'-xanthen]-6'-yl)oxy)acetate*

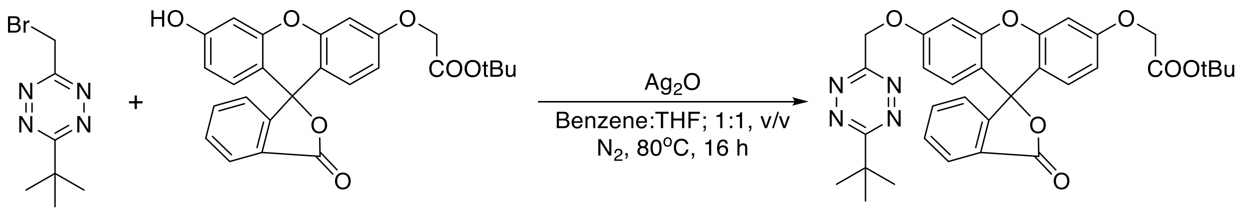


To an oven-dried two-neck flask equipped with a reflux condenser and a stir bar was added 3-(bromomethyl)-6-(tert-butyl)-1,2,4,5-tetrazine (42.7 mg, 0.18 mmol) and tert-butyl 2-((3'-

hydroxy-3-oxo-3H-spiro[isobenzofuran-1,9'-xanthen]-6'-yl)oxy)acetate[2] (55.0 mg, 0.12 mmol) in a mixture of anhydrous benzene and THF [0.05 M]. To the stirring solution was added solid silver(I) oxide (48.5 mg, 0.21 mmol) in a single portion. The flask was sealed, put under an atmosphere of nitrogen, and heated to 80°C. The reaction was stirred for 16 hours before allowing the reaction mixture to cool to room temperature. The cooled reaction mixture was filtered through celite and concentrated under reduced pressure for purification by silica column chromatography (EA:Hex, 4:1, v/v, $R_f = 0.4$) to afford the desired product as an orange solid (7 mg, 10%). $^1$H NMR (400 MHz, $CDCl_3$) δ 8.36 (d, J = 7.6 Hz, 1H), 7.79 – 7.67 (m, 2H), 7.32 (d, J = 7.6 Hz, 1H), 7.15 (s, 1H), 7.00 – 6.82 (m, 3H), 6.55 (d, J = 9.2 Hz, 1H), 6.45 (s, 1H), 5.74 (s, 2H), 4.48 (q, J = 15.6 Hz, 2H), 1.61 (s, 9H), 1.37 (s, 9H). HRMS (ESI): calcd. for $C_{33}H_{32}N_4O_7$ $[M+H]^+$ 597.2344, found 597.2340.

*2-((3'-((6-(tert-butyl)-1,2,4,5-tetrazin-3-yl)methoxy)-3-oxo-3H-spiro[isobenzofuran-1,9'-xanthen]-6'-yl)oxy)acetic acid (**4b**)*

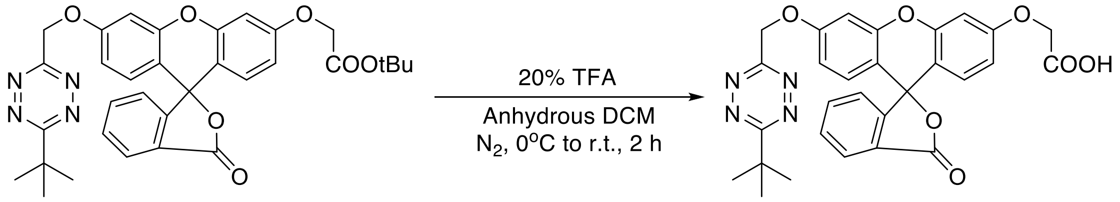


To an oven-dried two-neck flask equipped with a stir bar was added tert-butyl 2-((3'-((6-(tert-butyl)-1,2,4,5-tetrazin-3-yl)methoxy)-3-oxo-3H-spiro[isobenzofuran-1,9'-xanthen]-6'-yl)oxy)acetate (5 mg, 8.38 μmol) in anhydrous DCM, sealed. The flask was cooled to 0°C and put under an atmosphere of nitrogen. To the stirring solution was added TFA dropwise. Upon completion of addition, the ice bath was removed and allowed to stir at room temperature. The reaction was monitored by TLC and when deemed complete (2 hours), volatiles were removed under reduced pressure. The resulting residue was then co-evaporated with toluene (2x) and re-dissolved in methanol for purification by semi-preparative reverse phase HPLC using a Gemini C18 column (5 μM, 250×10 mm, Phenomenex, USA) with a mobile phase consisting of A (0.1% TFA in water) and B (acetonitrile) running a gradient from 40% to 55% component B over a 6 minute timeframe (desired compound retention time 5.3 minutes) to afford the desired product as an orange solid (2.5 mg, 55%). $^1$H NMR (500 MHz, DMSO-$d_6$) δ (Rotational

isomers present, major peaks reported when possible) 10.17 (bs, 1H), 8.00 (d, $J$ = 7.6 Hz, 1H), 7.79 (t, $J$ = 5.5 Hz, 1H), 7.74 – 7.69 (m, 2H), 7.29 (d, $J$ = 7.6 Hz, 1H), 7.17 (d, $J$ = 2.6 Hz, 1H), 7.03 (dd, $J$ = 8.9, 2.5 Hz, 1H), 6.86 (s, 1H), 6.70 (s, 1H), 6.58 (s, 1H), 5.80 (s, 2H), 4.59 – 4.55 (m, 2H), 1.52 (s, 9H). HRMS (ESI): calcd. for $C_{29}H_{24}N_4O_7$ $[M+H]^+$ 541.1718, found 541.1723.

*N-(4-(((2-amino-9H-purin-6-yl)oxy)methyl)benzyl)-2-((3'-((6-(tert-butyl)-1,2,4,5-tetrazin-3-yl)methoxy)-3-oxo-3H-spiro[isobenzofuran-1,9'-xanthen]-6'-yl)oxy)acetamide (**4b-BG**)*

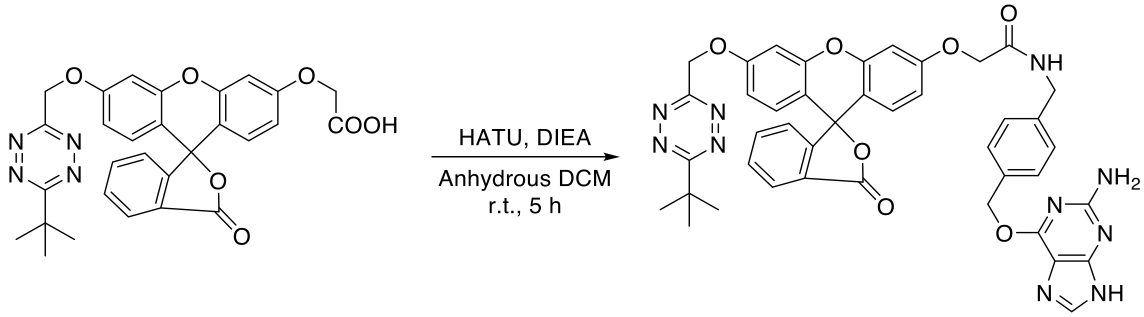


To an oven-dried 0.5 dram glass vial equipped with a stir bar was added 2-((3'-((6-(tert-butyl)-1,2,4,5-tetrazin-3-yl)methoxy)-3-oxo-3H-spiro[isobenzofuran-1,9'-xanthen]-6'-yl)oxy)acetic acid (1 mg, 1.85 µmol), 6-((4-(aminomethyl)benzyl)oxy)-9H-purin-2-amine (0.6 mg, 2.22 µmol), and DIEA (1 µL, 4.63 µmol) in anhydrous DCM [0.1 M]. To the stirring solution was added HATU (0.91 mg, 2.41 µmol) in a single portion and the reaction was allowed to proceed for 5 hours before the mixture was filtered through a short celite plug and volatiles removed under reduced pressure. The resulting residue was re-dissolved in methanol for purification by semi-preparative reverse phase HPLC using a Gemini C18 column (5 µM, 250×10 mm, Phenomenex, USA) with a mobile phase consisting of A (0.1% TFA in water) and B (acetonitrile) running a gradient from 30% to 33% component B over a 38.5 minute timeframe (desired compound retention time 22.8 minutes) to afford the desired product as an orange solid (0.15 mg, 10%). HRMS (ESI): calcd. for $C_{42}H_{36}N_{10}O_7$ $[M+H]^+$ 793.2841, found 793.2838.

*3-(tert-butyl)-6-(doxorubicin)-1,2,4,5-tetrazine (**Tz-Dox, 5**)*

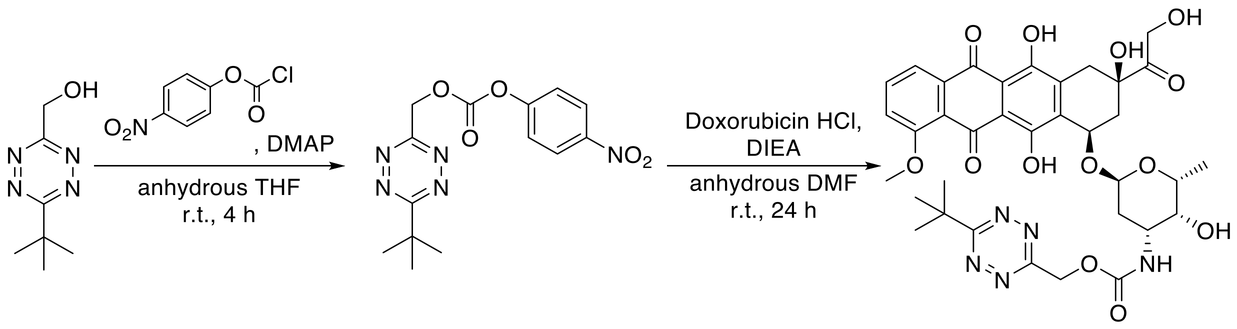


To an oven-dried round-bottom flask equipped with a stir bar was added (6-(tert-butyl)-1,2,4,5-tetrazin-3-yl)methanol (16.8 mg, 0.1 mmol) and solid 4-nitrophenyl chloroformate (24.2 mg, 0.12 mmol) in anhydrous THF [0.2 M]. To the stirring solution was added DMAP (24.4 mg, 0.2 mmol) in a single portion. The reaction mixture was stirred for 4 hours before being quenched by the addition of water (10 mL) and diluted with DCM (10 mL). The layers were separated, and the organic layer was washed with brine (1 x 10 mL), dried with $Na_2SO_4$, filtered, and concentrated to afford the chloroformate intermediate, which was used immediately in the next step without further purification. The chloroformate intermediate was dissolved in anhydrous DMF [0.15 M] and doxorubicin hydrochloride (70 mg, 0.12 mmol) was added in a single portion. To the stirring solution was added DIEA (138 μL, 0.8 mmol) and the mixture was allowed to stir at room temperature in the dark for 24 h before volatiles were removed under reduced pressure. The resulting residue was re-dissolved in methanol for purification by semi-preparative reverse phase HPLC using a Gemini C18 column (5 μM, 250×10 mm, Phenomenex, USA) with a mobile phase consisting of A (0.1% TFA in water) and B (acetonitrile) running a gradient from 25% to 72% component B over a 17 minute timeframe (desired compound retention time 16.6 minutes) to afford the desired product as a red solid (4 mg, 5%). $^1$H NMR (400 MHz, DMSO-$d_6$) δ 13.24 (s, 1H), 7.89 (d, J = 2.0 Hz, 1H), 7.88 (s, 1H), 7.67 – 7.60 (m, 1H), 7.21 (d, J = 8.0 Hz, 1H), 5.48 (q, J = 14.6 Hz, 2H), 5.21 (d, J = 2.0 Hz, 1H), 4.95 – 4.88 (m, 1H), 4.54 (s, 2H), 4.12 (dd, J = 12.6, 6.2 Hz, 1H), 3.97 (s, 2H), 3.73 – 3.63 (m, 2H), 2.93 (d, J = 5.6 Hz, 1H), 2.52 (s, 3H), 2.19 – 2.05 (m, 2H), 1.86 (td, J = 12.6, 3.6 Hz, 1H), 1.44 (s, 9H), 1.10 (d, J = 6.4 Hz, 2H). HRMS (ESI): calcd. for $C_{35}H_{39}N_5O_{13}$ $[M+Na]^+$ 760.2437, found 760.2433.

## Photospectrometric Analysis of Bimolecular Reaction

In 96-well clear-bottom plates, tetrazines were incubated at T = 37°C for 10 minutes to equilibrate the temperature. A pre-warmed (T = 37°C) solution of the corresponding dienophile was added in to initiate the reaction. The reactions of tetrazines (c = 0.2 mM) with dienophiles (c = 2 - 10 mM) in DMSO:PBS pH 7.4 = 4:1 (v/v) at T = 37°C were followed by monitoring the disappearance of tetrazine at $\lambda_{abs}$ 530 nm in one minute intervals. $\lambda_{abs}$ 530 nm is a local absorbance maximum for tetrazines.

Observed rate constants were determined by fitting the time-dependent tetrazine absorbance signal to a single-exponential equation assuming pseudo-first order kinetics. Reported bimolecular rate constants ($k_2$) were calculated by either plotting observed values against concentration followed by a linear fit or by direct exponential curve fitting and dividing by the concentration of the excess component. All reaction kinetic experiments were performed in triplicates. The reported rate constants are the mean of the three individual rate measurements and the error is provided as the standard error (**Table S1**). All data processing was done on GraphPad Prism 8.0.

**Table S1.** Bi-molecular rate constants ($M^{-1}s^{-1}$) calculated from reactions in a DMSO:PBS pH 7.4 = 4:1 (v/v) system at T = 37°C with the listed isonitriles (n.d. = not determined, MeNCAc = methyl isocyanoacetate). [a] = determined by HPLC analysis due to absorbance overlap in UV spectrophotometer experiments. [b] = determined in a DMSO:Tris pH 8.0 = 4:1 (v/v) system at 37°C.

| Tetrazine | $k_{2,\ t\text{-}OcNC}$ | $k_{2,\ \text{n-BuNC}}$ | $k_{2,\ \text{TMS-MeNC}}$ | $k_{2,\ \text{MeNCAc}}$ |
|---|---|---|---|---|
| 4a | 0.137 ± 0.004 | 0.117 ± 0.001 | 0.344 ± 0.013 | 0.049 ± 0.009 |
| 4b[a] | n.d. | 0.301 ± 0.018 | n.d. | n.d. |
| 4b’ | n.d. | 0.216 ± 0.015 | 0.778 ± 0.047[b] | n.d. |
| 4c | n.d. | 0.167 ± 0.005 | 0.335 ± 0.010 | n.d. |
| 4d | n.d. | 0.190 ± 0.014 | 0.380 ± 0.029 | n.d. |
| 4e | 0.152 ± 0.008 | 0.065 ± 0.0003 | 0.193 ± 0.008 | n.d. |

## Human Serum Albumin vs. General Base Catalysis

In 96-well clear-bottom plates, the Tzmoc-caged pNA probe (4a) was incubated at T = 37 °C for 10 minutes to equilibrate the temperature. A pre-warmed (T = 37 °C) solution of n-BuNC was added in to initiate the reaction. The reactions of 4a (c = 8 μM) with n-BuNC (c = 6 mM) in DMSO:PBS pH 7.4 = 1:4 (v/v) +/- 2 mg/mL human serum albumin (HSA, obtained from Sigma-Aldrich (A9511) and used without further purification) or in DMSO:PBS pH 7.4 = 1:4 (v/v) + 2 mM tris-base to release pNA were followed by monitoring the appearance of pNA at $\lambda_{abs}$ 385 nm in one-minute intervals. $\lambda_{abs}$ 385 nm is a local absorbance maximum for pNA. The addition of 2 mM of tris-base (concentration equal to that of surface amines in HSA containing solutions) did not catalyze the release of pNA (**Fig. S1**).

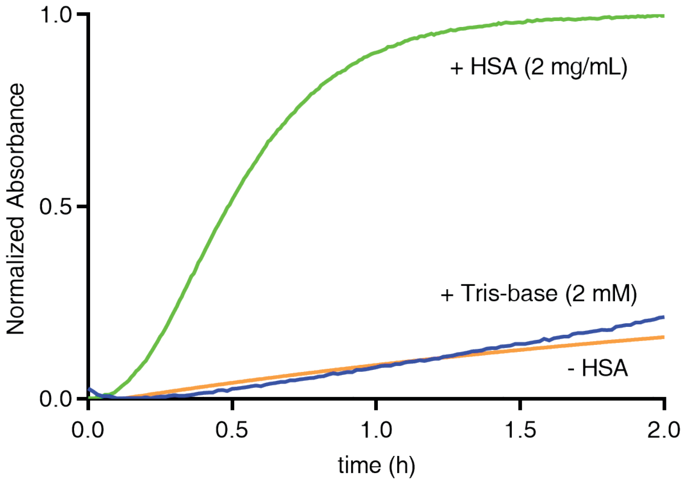


**Figure S1.** Kinetics of pNA release from 4a triggered by n-BuNC in the presence of HSA, tris-base, or alone (c(4a) = 8 μM, c(n-BuNC) = 6 mM, c(HSA) = 2 mg/mL, c(tris-base) = 2 mM, DMSO: PBS pH 7.4 (1:4, v/v), T = 37°C, λ = 385 nm, n = 3.

## Photospectrometric Analysis of Uncaging Step

### a. para-nitroaniline release kinetics and yields:

In 96-well clear-bottom plates, Tzmoc-caged pNA probes (4a, 4c, 4d, 4e) were incubated at T = 37 °C for 10 minutes to equilibrate the temperature. A pre-warmed (T = 37 °C) solution of the corresponding dienophile was added in to initiate the reaction. The reactions of tetrazines (c = 0.2 mM) with dienophiles (c = 2 mM) in DMSO:PBS pH 7.4 = 4:1 (v/v) at T = 37 °C to release pNA were followed by monitoring the appearance of pNA at $\lambda_{abs}$ = 435 nm in one-minute intervals. $\lambda_{abs}$ 435 nm is a shoulder absorbance peak from the local absorbance maximum of 385 nm for free pNA. For the reactions of tetrazines (c = 8 µM) with dienophiles (c = 6 mM) in DMSO:PBS pH 7.4 = 1:4 (v/v) at T = 37 °C, the release of pNA was followed by monitoring the appearance of pNA at $\lambda_{abs}$ = 385 nm in one-minute intervals. $\lambda_{abs}$ 385 nm is a local absorbance maximum for pNA. Release yields were calculated from endpoint measurements taken after 8 hours and plotted against a standard curve of para-nitroaniline obtained in the appropriate solvent systems (data not shown).

### b. para-nitroaniline release kinetics and yields with HSA:

In 96-well clear-bottom plates, the Tzmoc-caged pNA probe (4a) was incubated at T = 37 °C for 10 minutes to equilibrate the temperature. A pre-warmed (T = 37 °C) solution of the corresponding dienophile was added in to initiate the reaction. The reactions of tetrazines (c = 8 µM) with dienophiles (c = 6 mM) in DMSO:PBS pH 7.4 = 1:4 (v/v) + 2 mg/mL human serum albumin (HSA, obtained from Sigma-Aldrich (A9511) and used without further purification) to release pNA were followed by monitoring the appearance of pNA at $\lambda_{abs}$ 385 nm in one-minute intervals. $\lambda_{abs}$ 385 nm is a local absorbance maximum for pNA. Release yields were calculated from endpoint measurements taken after 8 hours and plotted against a standard curve of para-nitroaniline obtained in the DMSO:PBS pH 7.4 = 1:4 (v/v) solution containing 2 mg/mL HSA (data not shown).

### c. TzMe-caged 7-hydroxycoumarin (4b') release kinetics:

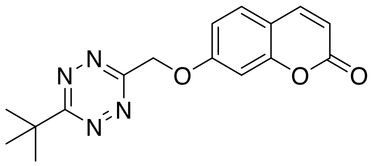


**Figure S2.** Structure of TzMe-caged 7-hydroxycoumarin dye used in this analysis.

In 96-well opaque plates, TzMe-caged 7-hydroxycoumarin (4b', **Fig. S2**) was incubated at T = 37 °C for 10 minutes to equilibrate the temperature. A pre-warmed (T = 37 °C) solution of the corresponding dienophile was added in to initiate the reaction. The reactions of tetrazines (c = 8 μM) with dienophiles (c = 6 mM) in DMSO:Tris pH 8.0 = 1:4 (v/v) at T = 37 °C to release 7-hydroxycoumarin were followed by monitoring the appearance of the fluorescent signal from 7-hydroxycoumarin ($\lambda_{ex}$ = 380 nm) at $\lambda_{em}$ 456 nm in one-minute intervals and using a cutoff filter of 455 nm (**Fig. S3**). Fluorescence turn-on fold was recorded upon completion of the reaction compared against control solutions (**Fig. S5**).

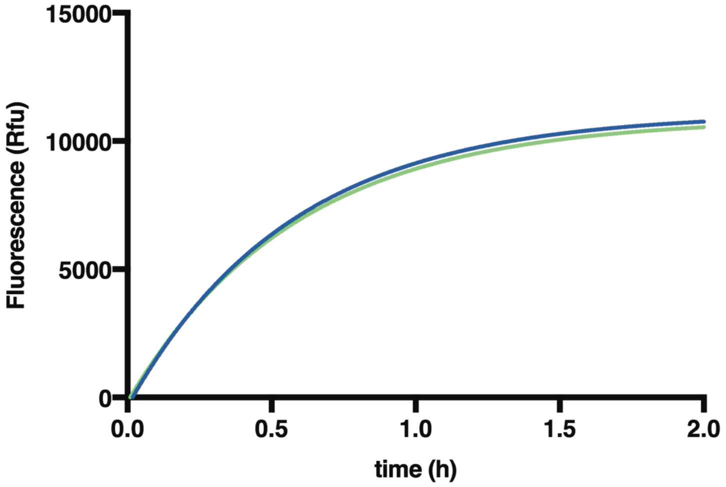


**Figure S3.** Kinetics of 7-hydroxycoumarin release (n = 3) from 4b' triggered by TMS-MeNC (green) or n-BuNC (blue); c(4b') = 8 μM, c(R-NC) = 6 mM, DMSO: Tris pH 8.0 (1:4), T = 37°C, $\lambda_{ex}$ = 380 nm, $\lambda_{em}$ = 456 nm.

**d. TzMe-caged O-carboxymethyl-fluorescein (4b) release kinetics and yields:**

In 96-well opaque plates, TzMe-caged O-carboxymethyl-fluorescein probe (4b) was incubated at T = 37 °C for 10 minutes to equilibrate the temperature. A pre-warmed (T = 37 °C) solution of the corresponding dienophile was added in to initiate the reaction. The reactions of tetrazines (c = 8 μM) with dienophiles (c = 6 mM) in DMSO:PBS pH 7.4 = 1:4 (v/v) to release O-carboxymethyl-fluorescein were followed by monitoring the appearance of the fluorescent signal from O-carboxymethyl-fluorescein ($\lambda_{ex}$ = 488 nm) at $\lambda_{em}$ 520 nm in one-minute intervals and using a cutoff filter of 515 nm (Figure 2f in the main text). Release yields were calculated from endpoint measurements taken after completion of the reaction and plotted against a standard curve of free O-carboxymethyl-fluorescein obtained in the appropriate solvent system (data not shown) and by UHPLC (A: 0.1% formic acid in $H_2O$; B = MeCN) (**Fig. S4**).

Top:

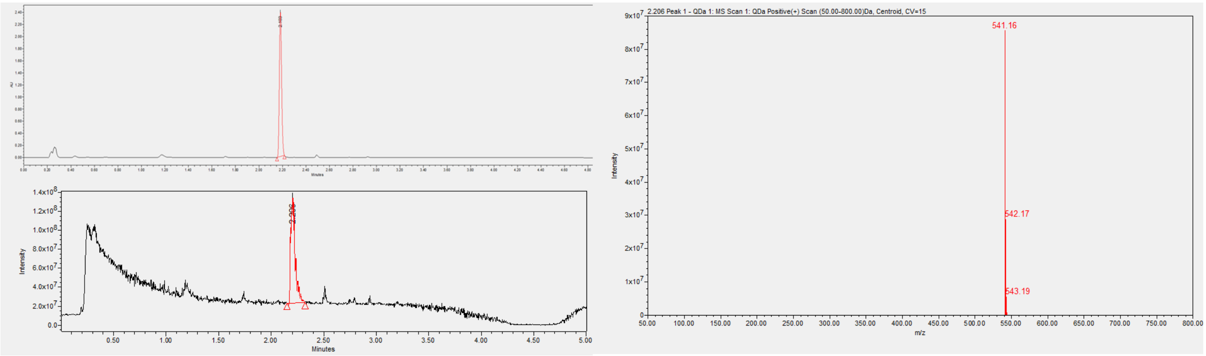


Bottom:

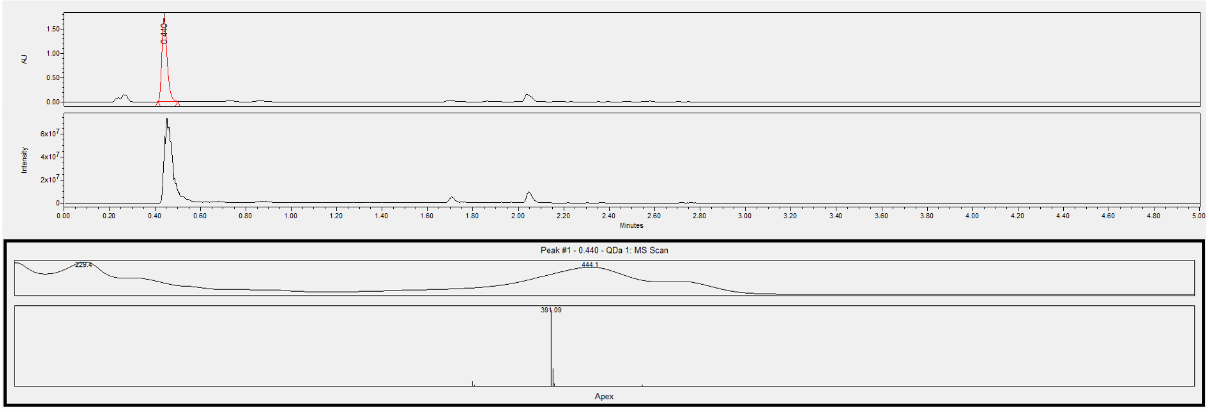


**Figure S4.** Top: UPLC trace of 4b (0.5 mM) at λ = 450 nm and associated low resolution TIC plot and extracted mass; Bottom: UPLC trace and associated low resolution mass of the release product from a reaction between 4b (0.5 mM) and TMS-MeNC (2 mM) after 1 h at 37°C in 4:1, DMSO:PBS pH 7.4

**e. Fluorescent turn-on fold upon release of TzMe from 4b' and 4b:**

Fluorescence turn-on fold was recorded upon completion of the reaction between TMS-MeNC and either 4b' or 4b and compared against control solutions (**Fig. S5**).

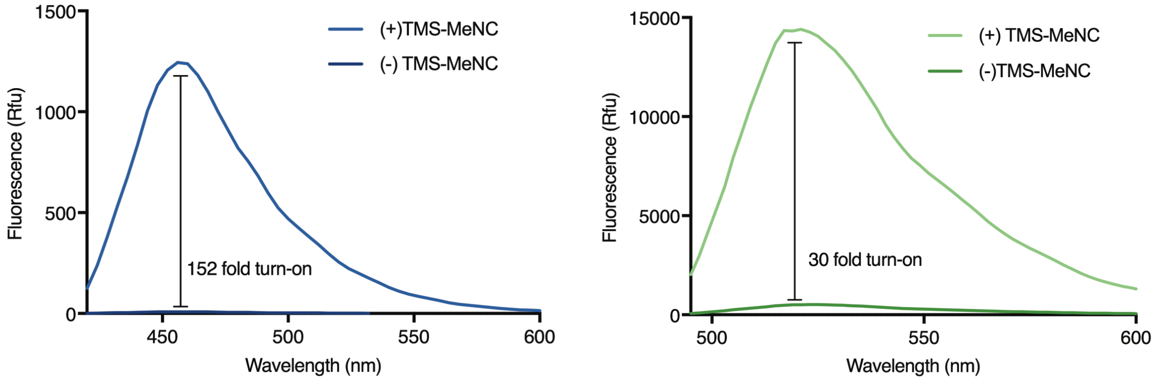


**Figure S5.** Turn-on fold of probes 4b' (left) and 4b (right) upon completion of TzMe deprotection in the presence of TMS-MeNC; left: (c(4b') = 8 µM, DMSO: Tris pH 8.0 (1:4), T = 37°C, $\lambda_{ex}$ = 380 nm); right: (c(4b) = 8 µM, DMSO: PBS pH 7.4 (1:4), T = 37°C, $\lambda_{ex}$ = 488 nm).

**f. First-order release rate constants ($k_1$) for release from probes 4a and 4b:**

$k_1$ values given in the main text for the release of para-nitroaniline from probe 4a (DMSO:PBS pH 7.4, 4:1, v/v at T=37°C) were calculated from the release of pNA from the postulated pyrazole intermediate by fitting the time-dependent absorbance curves using Wolfram Mathematica 11 to the rate law of a two-sequential first-order reactions as performed previously.[3]

$$[C] = (1 - \frac{-k_2 \exp(-k_{1,elim} t) + k_{1,elim} \exp(-k_2 t)}{k_{1,elim} - k_2})\, [A]_0$$

[C] = Concentration of C at time t; $k_2$ = bimolecular decay rate constant; $k_{1,\mathrm{elim}}$ = first order release rate constant; t = time; $[A]_0$ = initial concentration of A at time 0

$k_1$ value given in the main text for the release of O-carboxymethyl-fluorescein from probe 4b (DMSO:PBS pH 7.4 = 1:4 (v/v) at T = 37°C) was calculated from the release of O-carboxymethyl-fluorescein from the postulated pyrazole intermediate under the assumption that the bimolecular reaction rate rapid and negligible to fit the time-dependent fluorescence curves using the equation for a one-phase association given in GraphPad Prism 8.0.

## Human Liver Microsome Stability Assay

Stability of TzMe-caged 7-hydroxycoumarin (4b’, **Fig. S2**) was assessed in a human liver microsome assay at T = 37°C performed by Creative Bioarray (USA). Spiking solutions of test and reference compounds were prepared: 500 µM spiking solution: 5 µL of 10 mM stock solution of 4b’ into 95 µL of MeCN. 1.5 µL of the 500 µM spiking solution and 18.75 µL of 20 mg/mL liver microsomes solution was added into 479.75 µL of K-Buffer to obtain a 1.5 µM spiking solution in microsomes (0.75 mg/mL). Next, a 3X NADPH stock solution (6 mM, 5 mg/mL) was prepared by dissolving NADPH into buffer. 30 µL of the 1.5 µM spiking solution containing 0.75 mg/mL microsomes solution to the assay plates designated for different time points (0, 5, 15, 30, 45 min). For the 0-minute time point, 150 µL of MeCN containing an internal standard (IS) was added to the wells of the 0-minute plate and 15 µL of the 6 mM NADPH stock was added, while all other plates were incubated at 37°C. To the plates were then added 15 µL of the 6 mM NADPH stock solution to begin the measurement. At 5, 15, 30, and 45 minutes, 150 µL of MeCN containing IS was added to the wells of the corresponding plates to quench the reaction. Upon quenching, the plates were shaken for 10 minutes (600 rpm/min) followed by centrifuging at 6000 rmp for 15 minutes. 80 µL of the supernatant from each well was aliquoted and 120 µL of water was added for LC/MS/MS analysis (**Fig. S6**). Analysis was done in triplicate (n = 3). Creative Bioarray reported the half-life, $t_{1/2}$, and intrinsic clearance, $Cl_{int}$. $T_{1/2}$ was calculated with: $t_{1/2} = 0.693/K$ (K = rate constant from the plot of ln [concentration] vs. incubation time (**Fig. S6**)). $Cl_{int}$ was calculated with: $Cl_{int} = \left(\frac{0.693}{t_{\frac{1}{2}}}\right) \times \left(\frac{1}{microsomal\ protein\ cocentration\ \left(0.5\frac{mg}{mL}\right)}\right) \times Scaling\ Factors$ . (Scaling factor = (microsomal protein per gram of liver) × (liver weight per kilogram of body weight); Human = 1254.2)

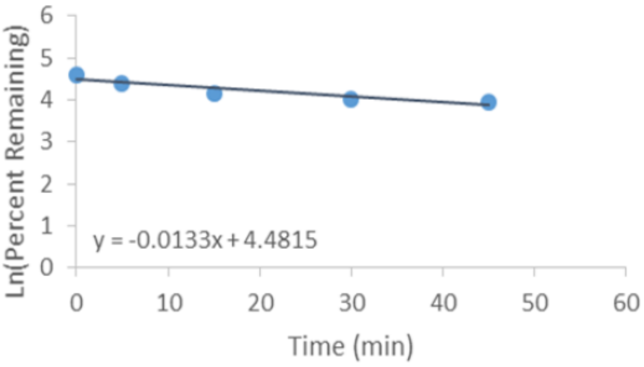


**Figure S6.** Graph of the natural log of the percent remaining of 4b' at indicated time-points in the human liver microsome assay; graph was used to calculate $t_{1/2}$ and $Cl_{int}$ using aforementioned formulas (Creative Bioarray, USA).

## Serum Stability of TzMe Probes

Stability of TzMe-caged O-carboxymethyl fluorescein (4b) was assessed in a dilute human serum solution. Solutions of 4b (c = 50 µM) in PBS: serum: DMSO (9:9:2, v/v/v) were thoroughly mixed and shaken at 37°C in the dark. Aliquots of 50 µL were taken at the indicated time points (5 min, 3 h, 6 h, 18 h, 24 h) and quenched by 100 µL methanol, followed by centrifugation at 13,300 rpm for 5 min. HPLC spectra (**Fig. S7**) were recorded at the time points at 440 nm to calculate the half-life of 4b in dilute serum ($t_{1/2}$ = 19 ± 4 h).

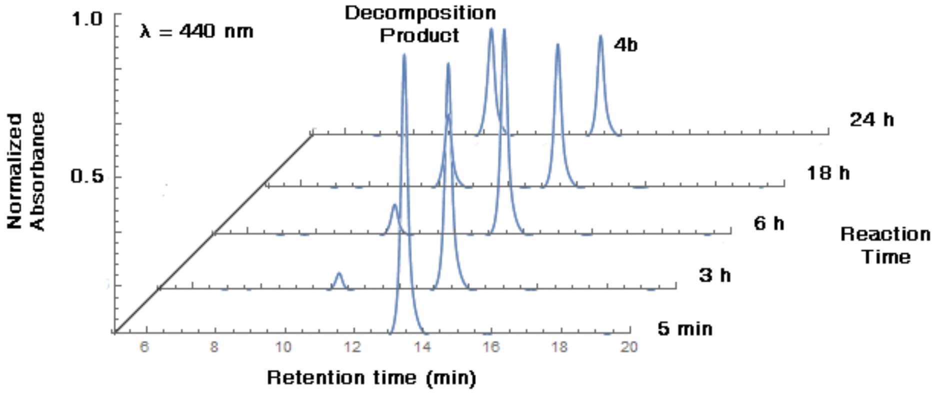


**Figure S7.** Representative spectra of HPLC analysis of the stability of 4b in human serum at various time points. The mobile phase A was water + 0.1% TFA and mobile phase B was acetonitrile. A gradient of 20-50% B ranging over 20 minutes at a flow rate of 0.6 mL/min was employed.

## Effects of Structural Modifications on TzMe probes

We investigated the release percentage of pNA from Tzmoc-caged pNA probes with modifications at the methylene position (4c, 4d) and at the 6' position (4e) triggered by n-BuNC (**Fig. S8**); we further compared the release rate of pNA release from probe 4a versus 4e triggered by n-BuNC (**Fig. S9**). The kinetics and release yields in the reaction between the modified probes and n-BuNC was performed analogous to as described in the previous section—"Photospectrometric Analysis of Uncaging Step."

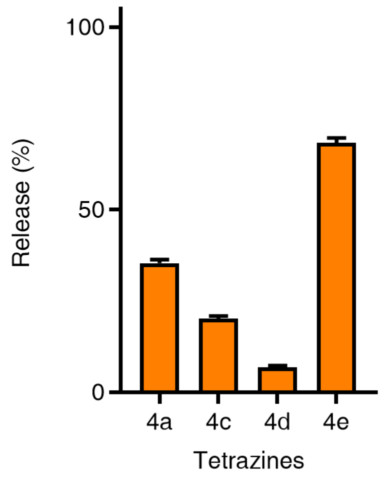


**Figure S8.** Bar plot for the release percentages of pNA from 4a, 4c, 4d, and 4e induced by n-BuNC (c(4x) = 0.2 mM, c(n-BuNC) = 2 mM, DMSO: PBS pH 7.4 (4:1), T = 37°C, t = 8 h).

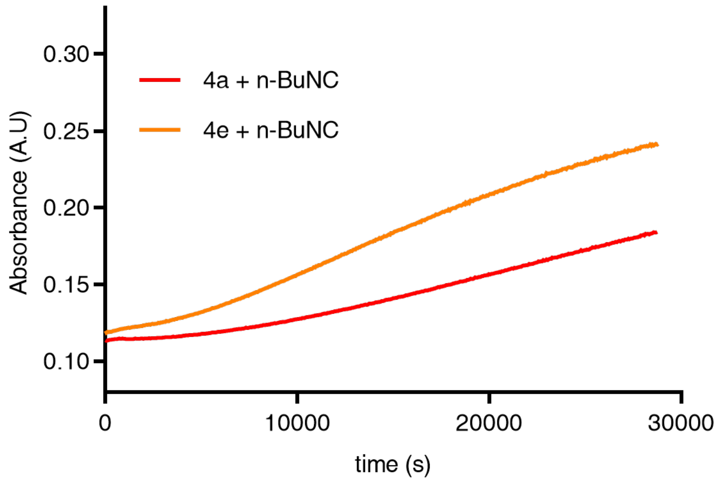


**Figure S9.** Kinetics of release of pNA from 4e compared to 4a (c(4a/4e) = 0.2 mM, c(n-BuNC) = 2 mM, DMSO: PBS pH 7.4 (4:1), T = 37°C, λ = 435 nm).

## Removal of TzMe groups by Alternative Dienophiles

We measured the release of para-nitroaniline or O-carboxymethyl fluorescein from 4a or 4b, respectively, triggered by reactions with several dienophiles (**Fig. S10**) other than isonitriles by photospectrometric assays performed on a multi-plate reader. The release percentages obtained, as described previously, for both pNA and O-carboxymethyl fluorescein, are reported in Fig. 3b in the main text.

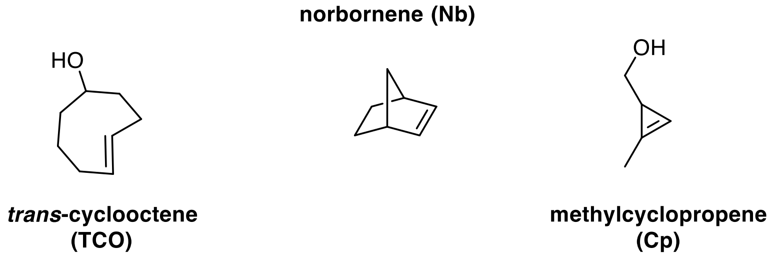


**Figure S10.** Structures of the alternative dienophiles used in this portion of the study.

## NMR Analyses

To analyze the possible reaction pathway (**Fig. S11**) for the release TzMe caged compounds, we performed various NMR studies. Stock solutions of isonitriles (20 mM) and tetrazines (40 mM) were prepared in DMSO-$d_6$. A separate stock solution of 15-crown-5-ether (internal standard, IS) in either $D_2O$ for experiments including 10% $D_2O$ or in DMSO-$d_6$ for experiments without $D_2O$ was prepared (10 mM). NMR experiments were performed on either a Varian Inova 500 MHz or a Varian Inova 600 MHz NMR spectrometer.

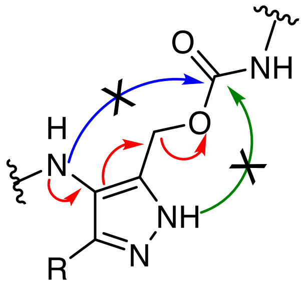


**Figure S11.** Possible reaction pathways leading to the release of amines or phenols. The blue and green arrows indicate release through a cyclization step providing a bicyclic compound and the desired release. The "X" indicates that these routes are unlikely because phenols can be released and due to the following NMR studies not showing a bicyclic compound following release.

## a. Reaction of Tzmoc-caged pNA (4a) with n-BuNC (Fig. S12):

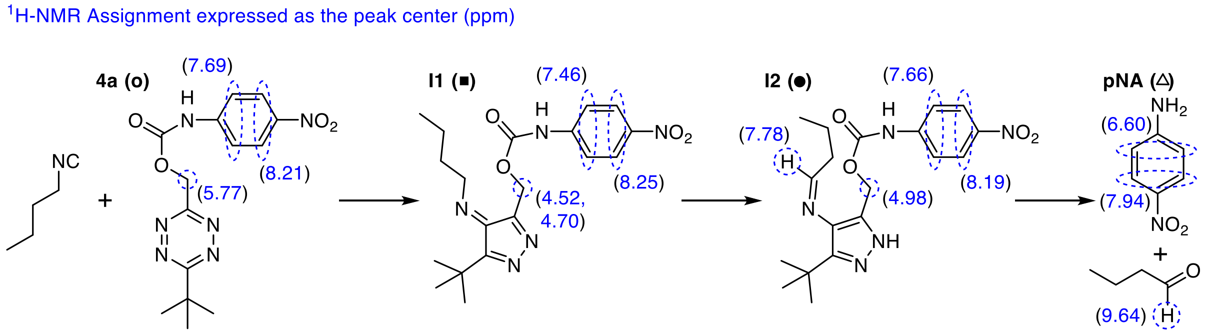


**Figure S12.** Observed compounds in NMR and proton assignments

In a standard 5 mm NMR tube, 75 µL of 4a (40 mM, DMSO-$d_6$) was combined with 375 µL of n-BuNC (20 mM, DMSO-$d_6$) followed by 50 µL of internal standard (IS) 15-crown-5-ether (10 mM, $D_2O$) for a final reaction sample containing 500 µL (DMSO-$d_6$:$D_2O$, 9:1, v/v) of 6 mM tetrazine, 15 mM isonitrile, and 1 mM IS. Time-dependent $^{1}$H-NMR experiments (n = 3) were conducted at T = 25°C on a Varian Inova 500 MHz (**Fig. S13**). Integration was normalized against the proton signal from the IS and plotted in figure 4c in the main text.

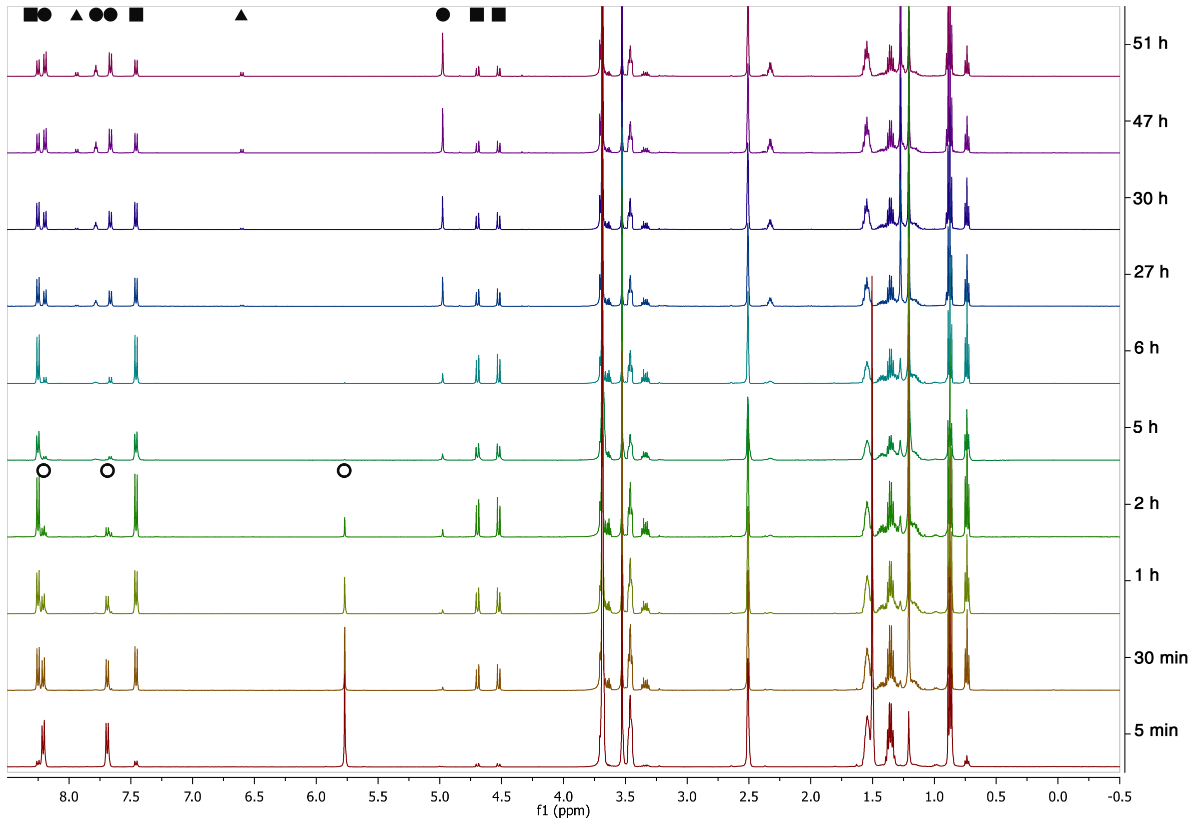


**Figure S13.** Spectra monitoring the reaction between 4a and n-BuNC

**b. Reaction of di-methyl-tetrazine with n-BuNC (Fig. S14):**

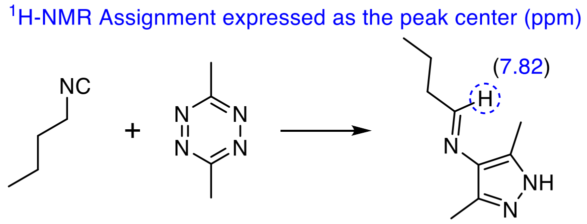


**Figure S14.** Observed compounds in NMR and proton assignment.

In a standard 5 mm NMR tube, 75 µL of di-methyl-tetrazine (40 mM, DMSO-$d_6$) was combined with 375 µL of n-BuNC (20 mM, DMSO-$d_6$) followed by 50 µL of internal standard (IS) 15-crown-5-ether (10 mM, DMSO-$d_6$) for a final reaction sample containing 500 µL (DMSO-$d_6$) of 6 mM tetrazine, 15 mM isonitrile, and 1 mM IS. Time-dependent $^1$H-NMR experiments were conducted at T = 25°C on a Varian Inova 500 MHz (**Fig. S15**).

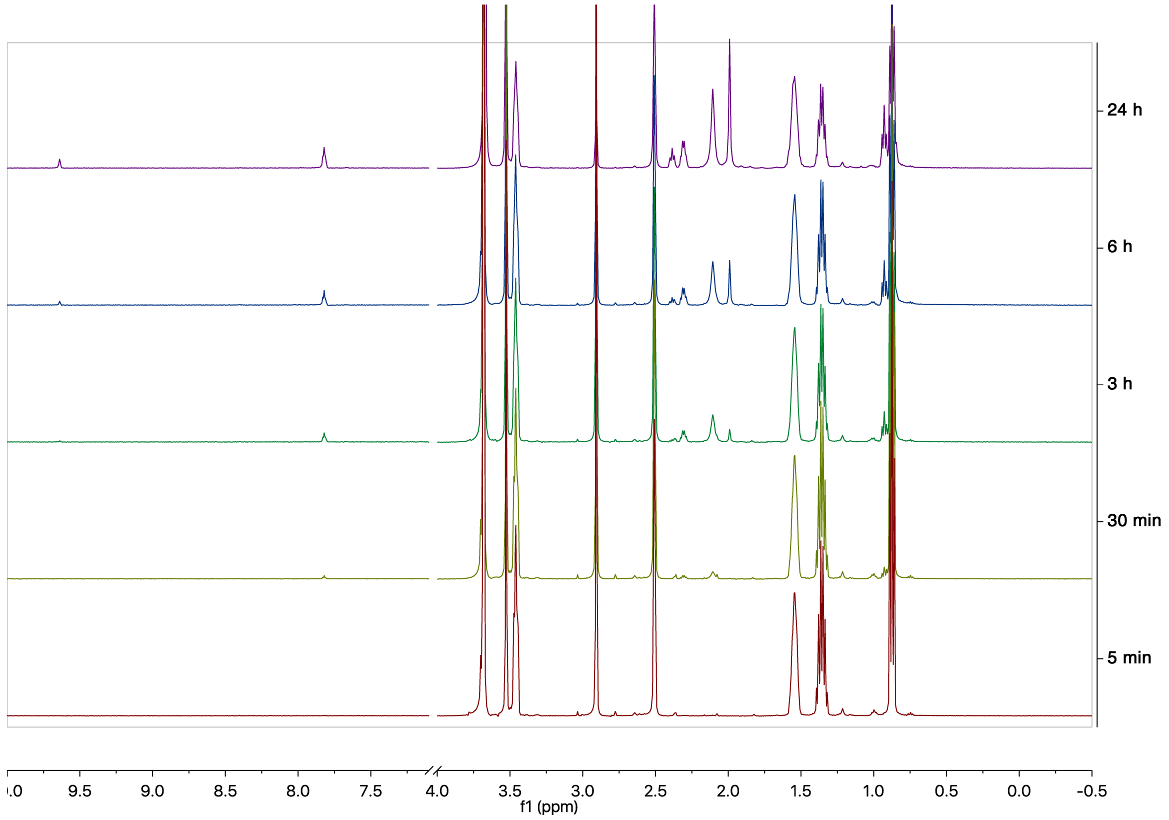


**Figure S15.** Spectra monitoring the reaction between di-methyl-tetrazine and n-BuNC.

### c. Reaction of di-*tert*-butyl-tetrazine with n-BuNC (Fig. S16):

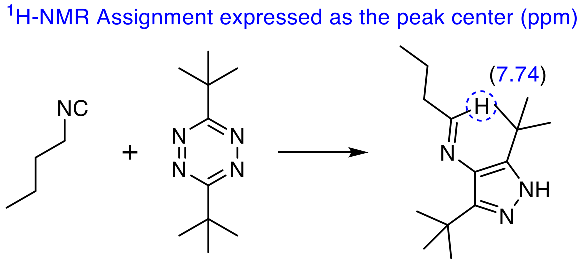


**Figure S16.** Observed compounds in NMR and proton assignment.

In a standard 5 mm NMR tube, 75 µL of bis-tert-butyltetrazine (40 mM, DMSO-$d_6$) was combined with 375 µL of n-BuNC (20 mM, DMSO-$d_6$) followed by 50 µL of internal standard (IS) 15-crown-5-ether (10 mM, DMSO-$d_6$) for a final reaction sample containing 500 µL (DMSO-$d_6$) of 6 mM tetrazine, 15 mM isonitrile, and 1 mM IS. Time-dependent $^1$H-NMR experiments were conducted at T = 25°C on a Varian Inova 500 MHz (**Fig. S17**).

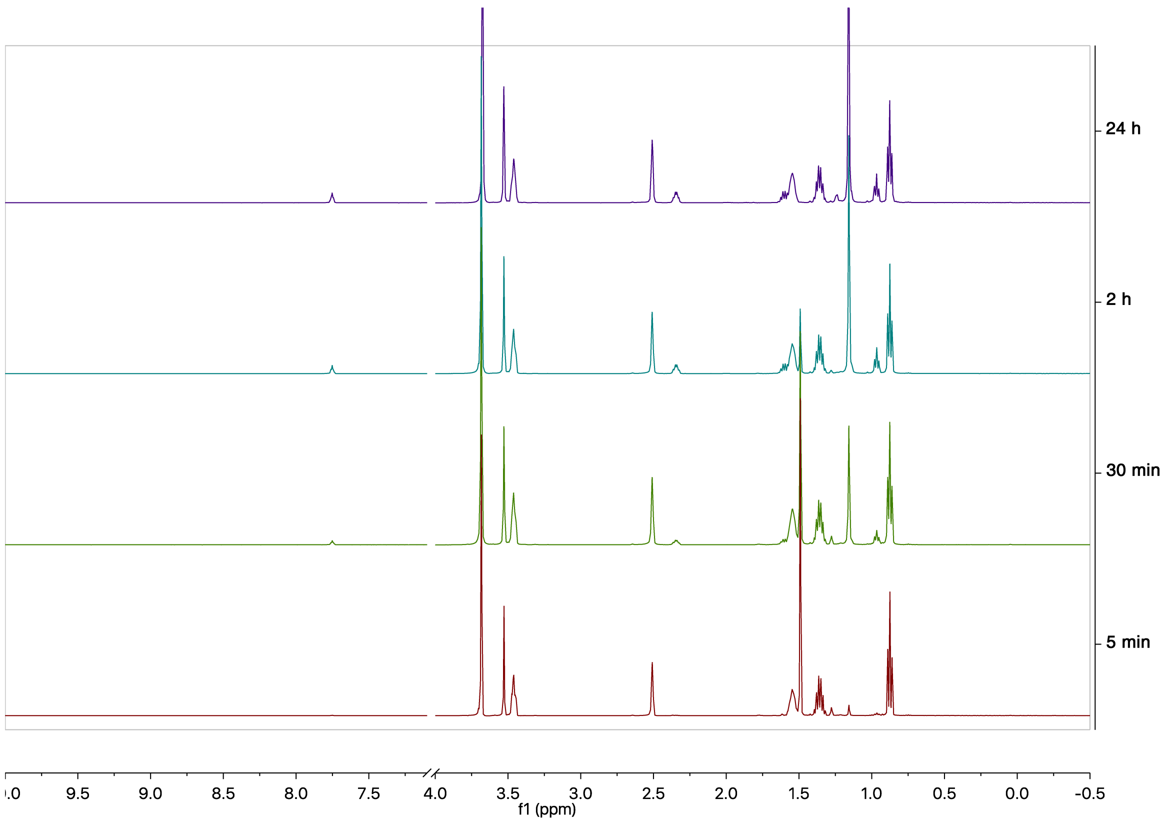


**Figure S17.** Spectra monitoring the reaction between di-*tert*-butyl-tetrazine and n-BuNC.

**d. Reaction of Tzmoc-caged pNA (4a) with TMS-MeNC (10% $D_2O$ in DMSO-$d_6$) (Fig. S18):**

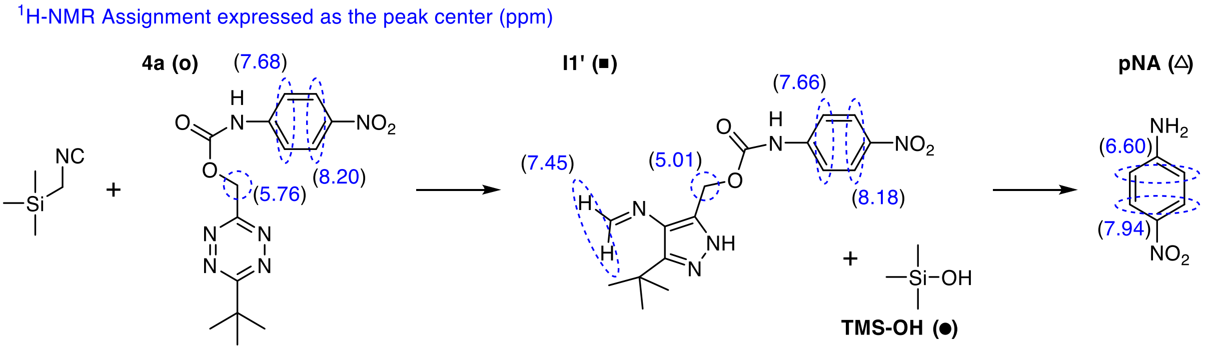


**Figure S18.** Observed compounds in NMR and proton assignment.

In a standard 5 mm NMR tube, 75 µL of 4a (40 mM, DMSO-$d_6$) was combined with 375 µL of TMS-MeNC (20 mM, DMSO-$d_6$) followed by 50 µL of internal standard (IS) 15-crown-5-ether (10 mM, $D_2O$) for a final reaction sample containing 500 µL (DMSO-$d_6$:$D_2O$, 9:1, v/v) of 6 mM tetrazine, 15 mM isonitrile, and 1 mM IS. Time-dependent $^1$H-NMR experiments (n = 3) were conducted at T = 25°C on a Varian Inova 500 MHz (**Fig. S19**). Integration was normalized against the proton signal from the IS and plotted in figure 4f in the main text.

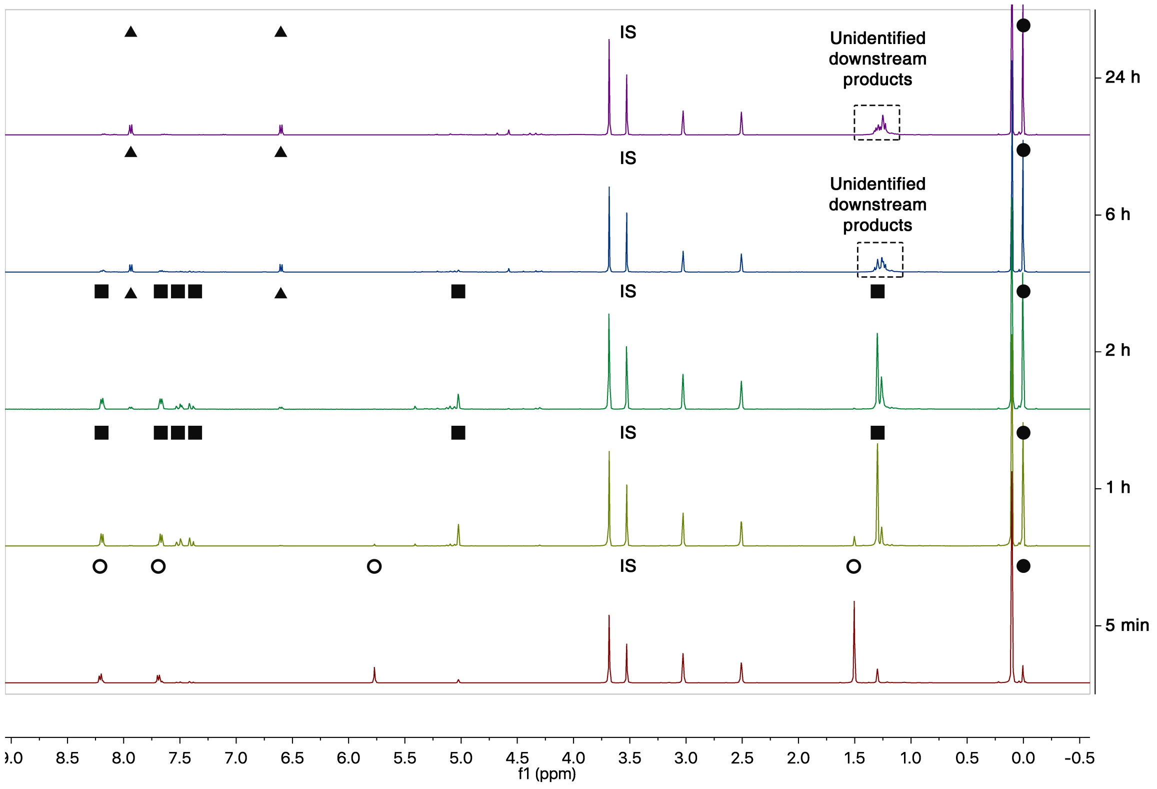


**Figure S19.** Spectra monitoring the reaction between 4a and TMS-MeNC.

## e. Reaction of Tzmoc-caged pNA (4a) with TMS-MeNC (100% DMSO-$d_6$) (Fig. S20):

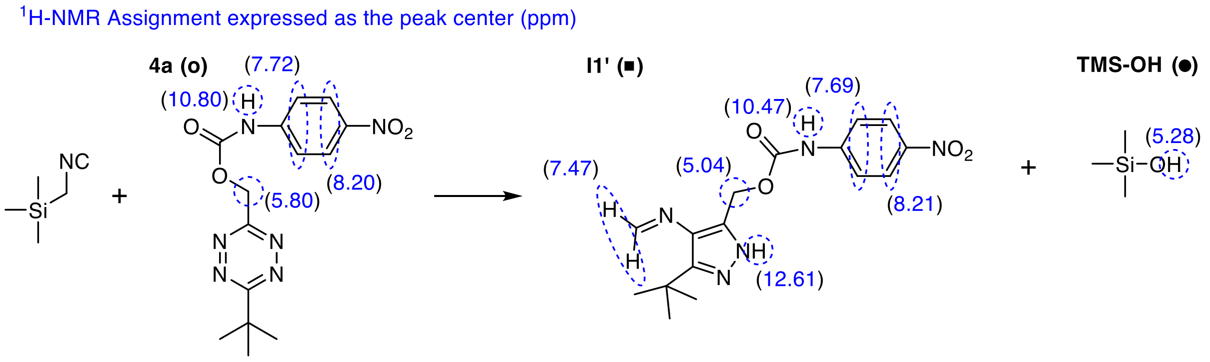


**Figure S20.** Observed compounds in NMR and proton assignment.

In a standard 5 mm NMR tube, 75 µL of 4a (40 mM, DMSO-$d_6$) was combined with 375 µL of TMS-MeNC (20 mM, DMSO-$d_6$) followed by 50 µL of internal standard (IS) 15-crown-5-ether (10 mM, DMSO-$d_6$) for a final reaction sample containing 500 µL (DMSO-$d_6$) of 6 mM tetrazine, 15 mM isonitrile, and 1 mM IS. Time-dependent $^{1}$H-NMR experiments were conducted at T = 25°C on a Varian Inova 500 MHz (**Fig. S21**).

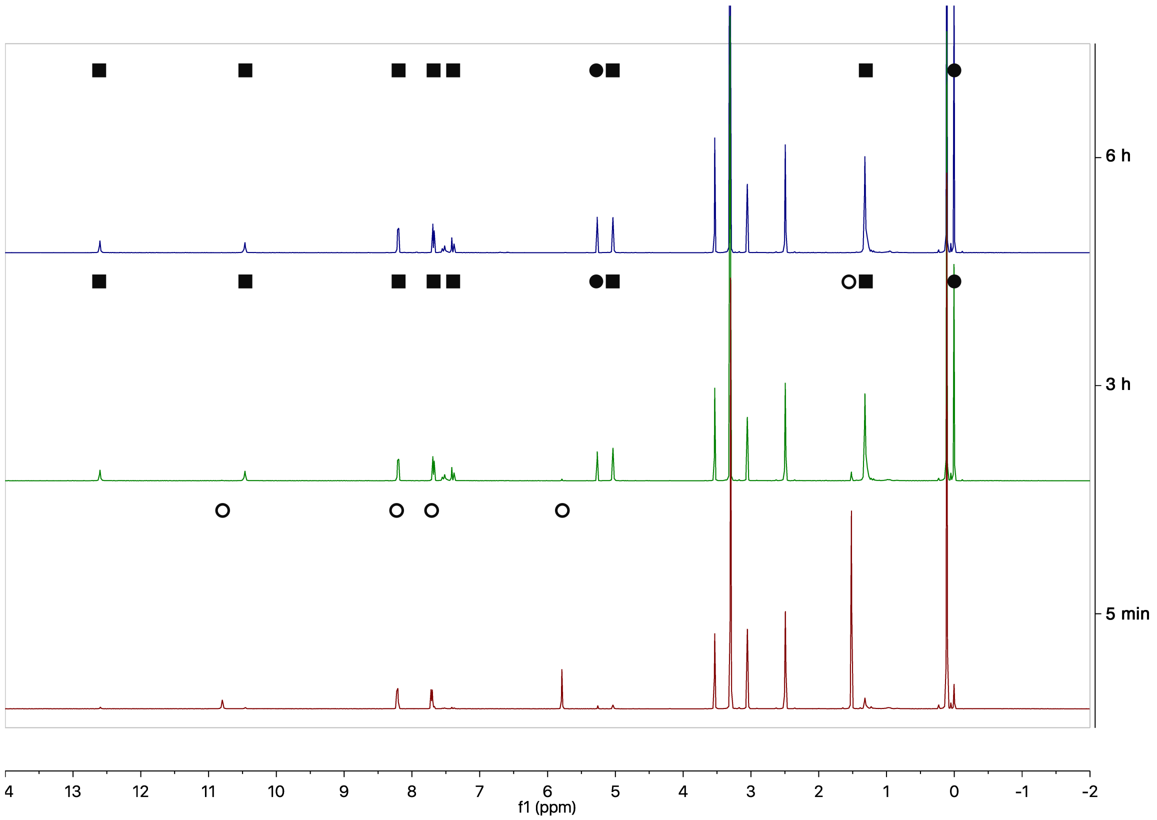


**Figure S21.** Full spectra monitoring the reaction between 4a and TMS-MeNC.

## f. Reaction of di-*tert*-butyl-tetrazine with TMS-MeNC (Fig. S22):

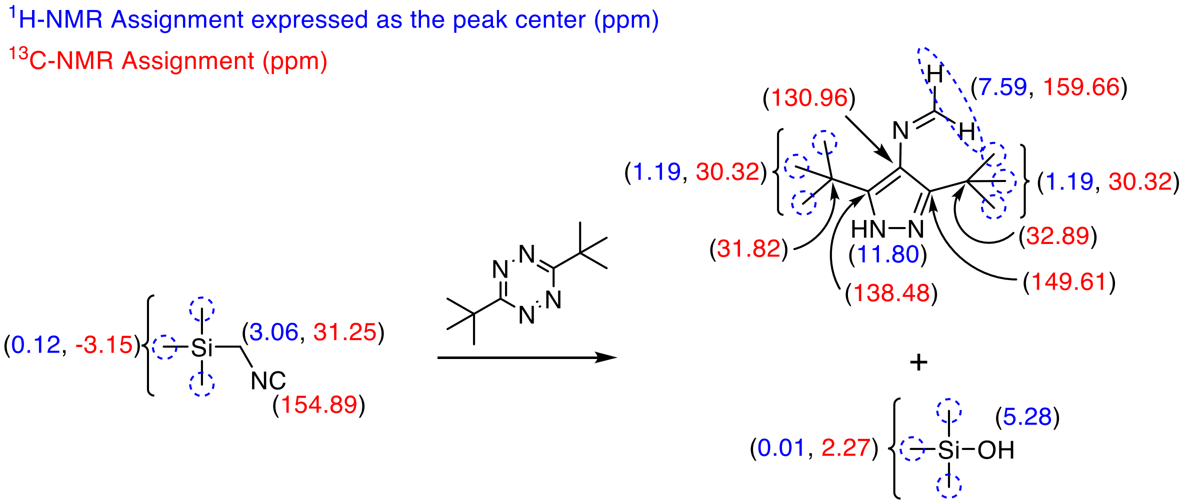


**Figure S22.** Observed compounds in NMR and proton/carbon assignment.

In a standard 5 mm NMR tube, 75 µL of bis-tert-butyltetrazine (40 mM, DMSO-$d_6$) was combined with 300 µL of n-BuNC (20 mM, DMSO-$d_6$) followed by 125 µL of DMSO-$d_6$ for a final reaction sample containing 500 µL (DMSO-$d_6$) of 6 mM tetrazine and 12 mM isonitrile. The internal standard was omitted in these studies to avoid artifacts arising from interactions with 15-crown-5 ether. Signals were referenced to and $^1$H-NMR integration values normalized to the DMSO-$d_6$ solvent peak (2.50 ppm, 3.75 integration). Time-dependent $^1$H-NMR experiments (not shown) were conducted and at t = 24 h, a $^1$H-NMR was recorded (**Fig. S23**), followed by gCOSY (**Fig. S24**), $^1$H-$^{13}$C gHSQC (**Figs. S25, 26**), and $^1$H-$^{13}$C gHMBC (**Fig. S27**) experiments taken in succession at T = 25°C on a Varian Inova 600 MHz with an HCN cryogenic probe.

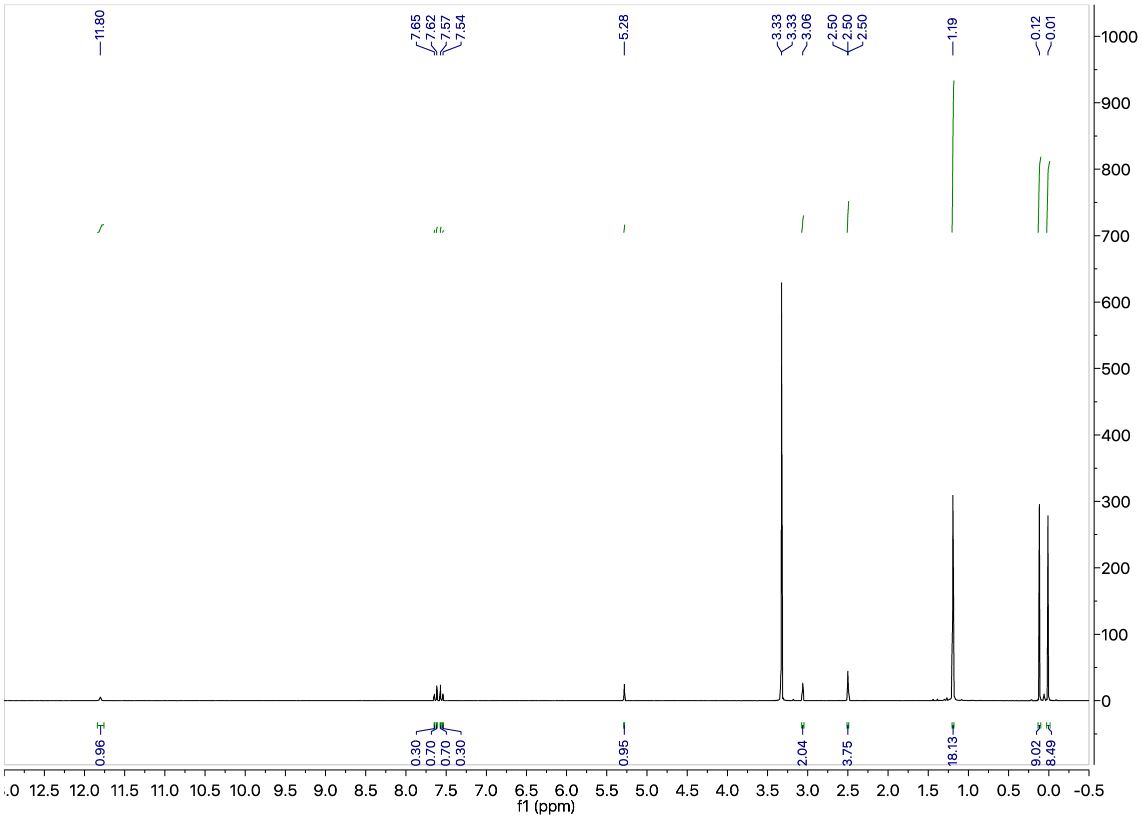


**Figure S23.** $^{1}$H-NMR taken at t = 24 h in DMSO-$d_6$ at T = 25°C

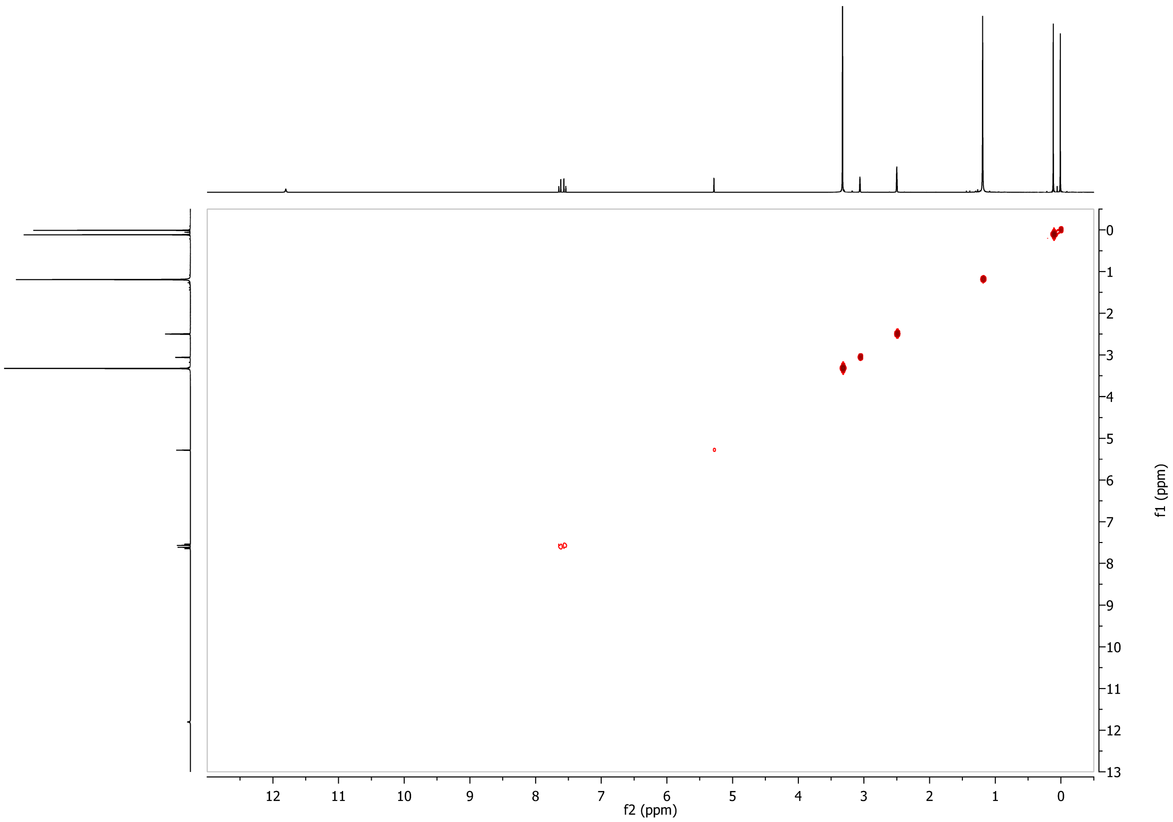


**Figure S24.** gCOSY taken following the $^{1}$H experiment in DMSO-$d_6$ at T = 25°C

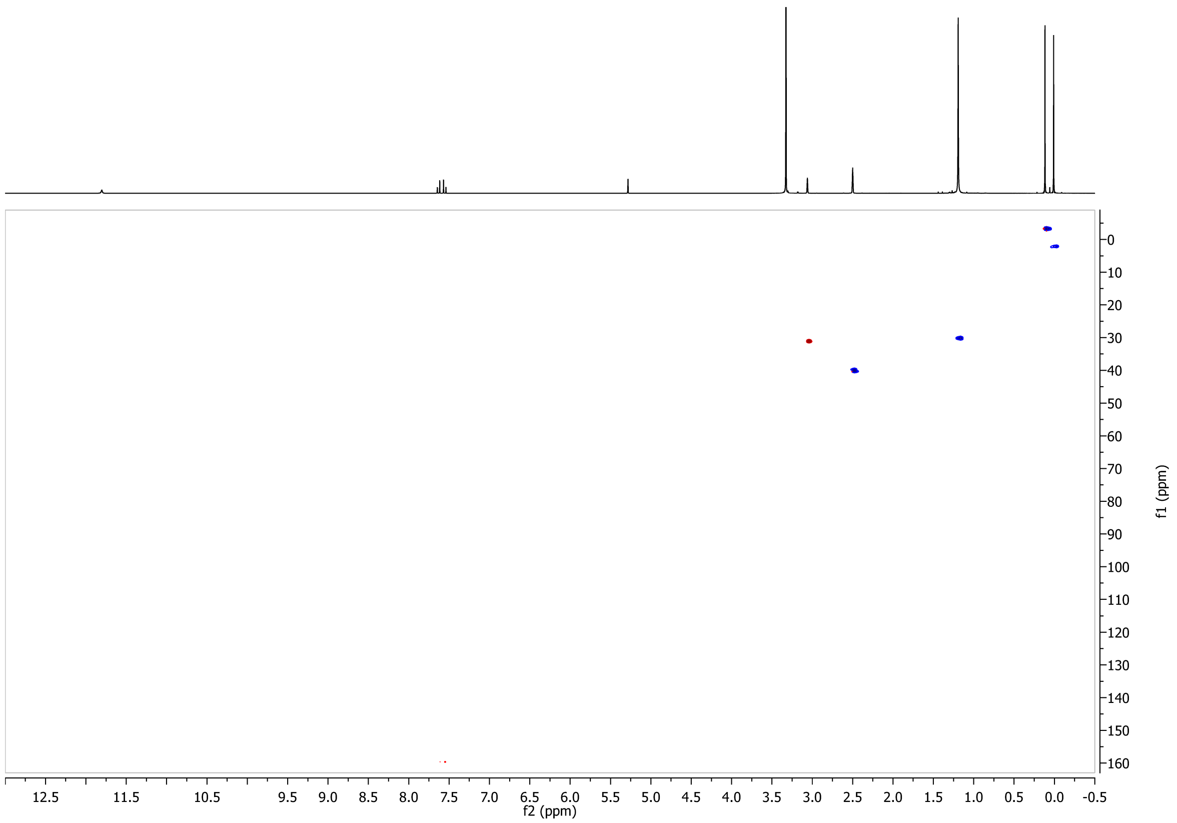


**Figure S25.** $^{1}H$-$^{13}C$ gHSQC taken following the gCOSY experiment in DMSO-$d_6$ at T = 25°C

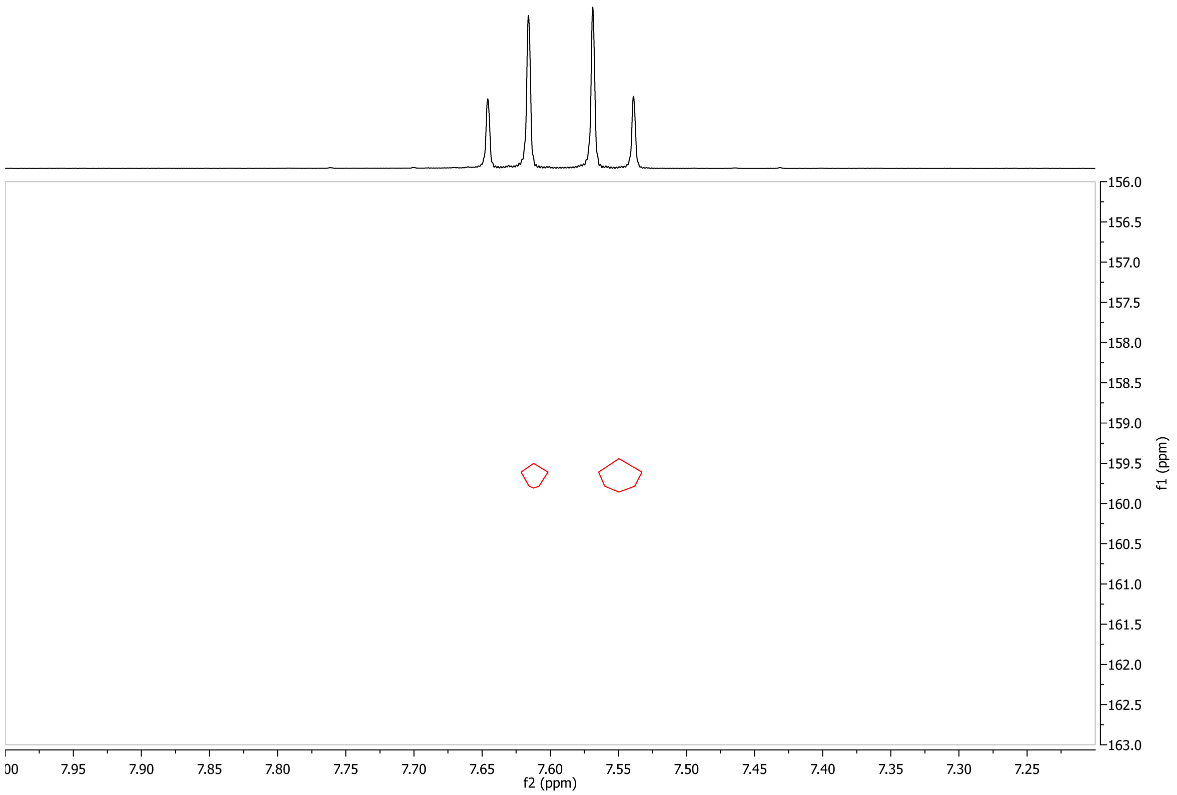


**Figure S26.** $^{1}H$-$^{13}C$ gHSQC taken following the gCOSY experiment in DMSO-$d_6$ at T = 25°C zoomed into the imine peak region.

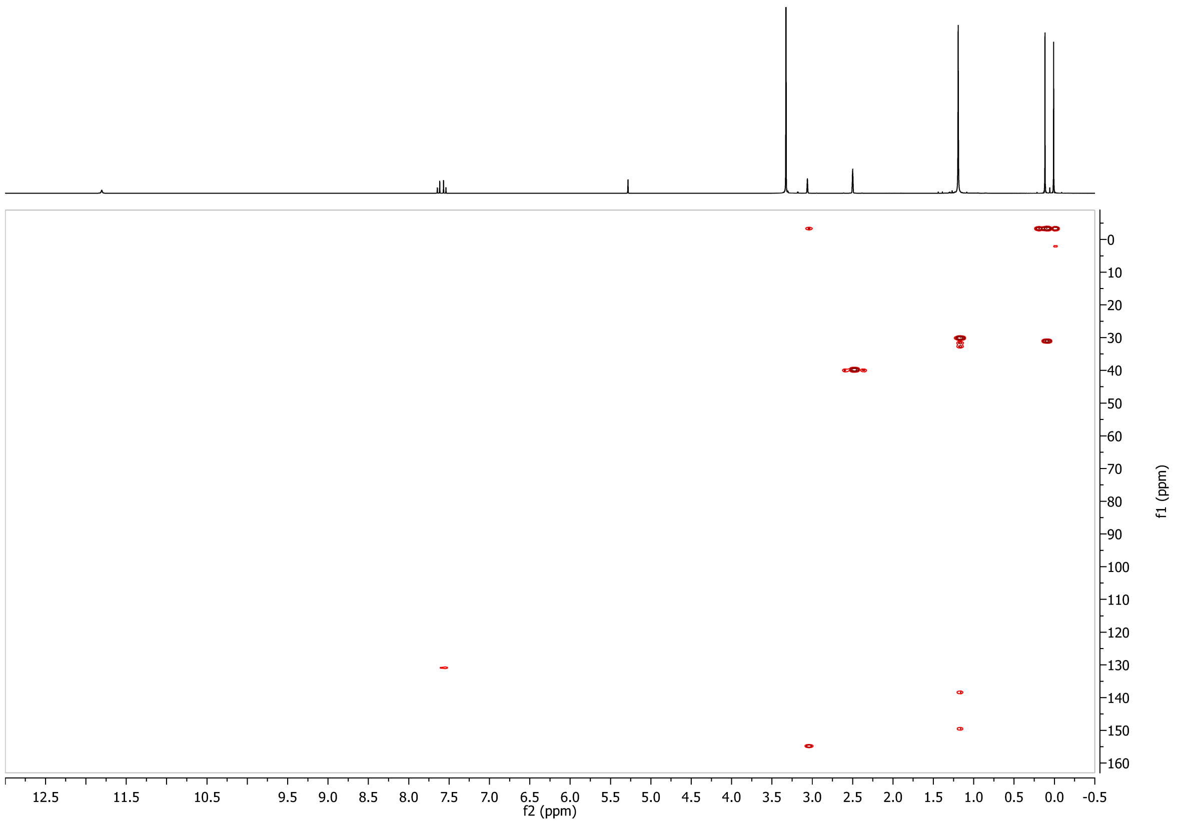


**Figure S27.** $^{1}H$-$^{13}C$ gHMBC taken following the $^{1}H$-$^{13}C$ gHSQC experiment in DMSO-$d_6$ at T = 25°C

## g. TzMe-caged 7-hydroxycoumarin (4b') reaction with n-BuNC (Fig. S28):

$^{1}$H-NMR Assignment expressed as the peak center (ppm)

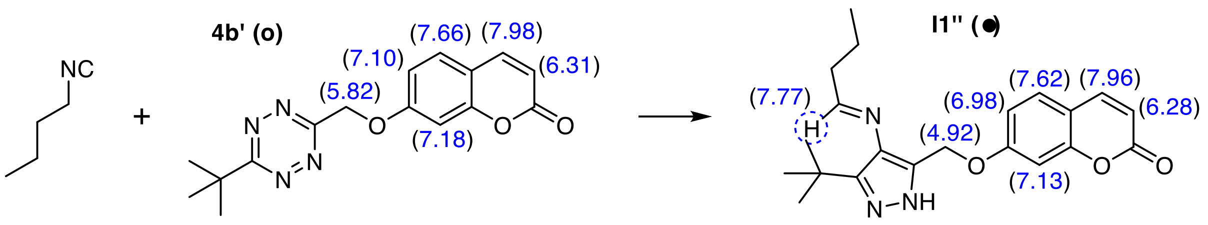


**Figure S28.** Observed compounds in NMR and proton assignment.

In a standard 5 mm NMR tube, 75 µL of 4b' (40 mM, DMSO-$d_6$) is combined with 375 µL of n-BuNC (20 mM, DMSO-$d_6$) followed by 50 µL of internal standard (IS) 15-crown-5-ether (10 mM, $D_2O$) for a final reaction sample containing 500 µL (DMSO-$d_6$:$D_2O$, 9:1, v/v) of 6 mM tetrazine, 15 mM isonitrile, and 1 mM IS. Time-dependent $^{1}$H-NMR experiments were conducted at T = 25°C on a Varian Inova 500 MHz; a focused spectra view is presented below (**Fig. S29**), revealing the concomitant appearance of I1'' with the disappearance of 4b'.

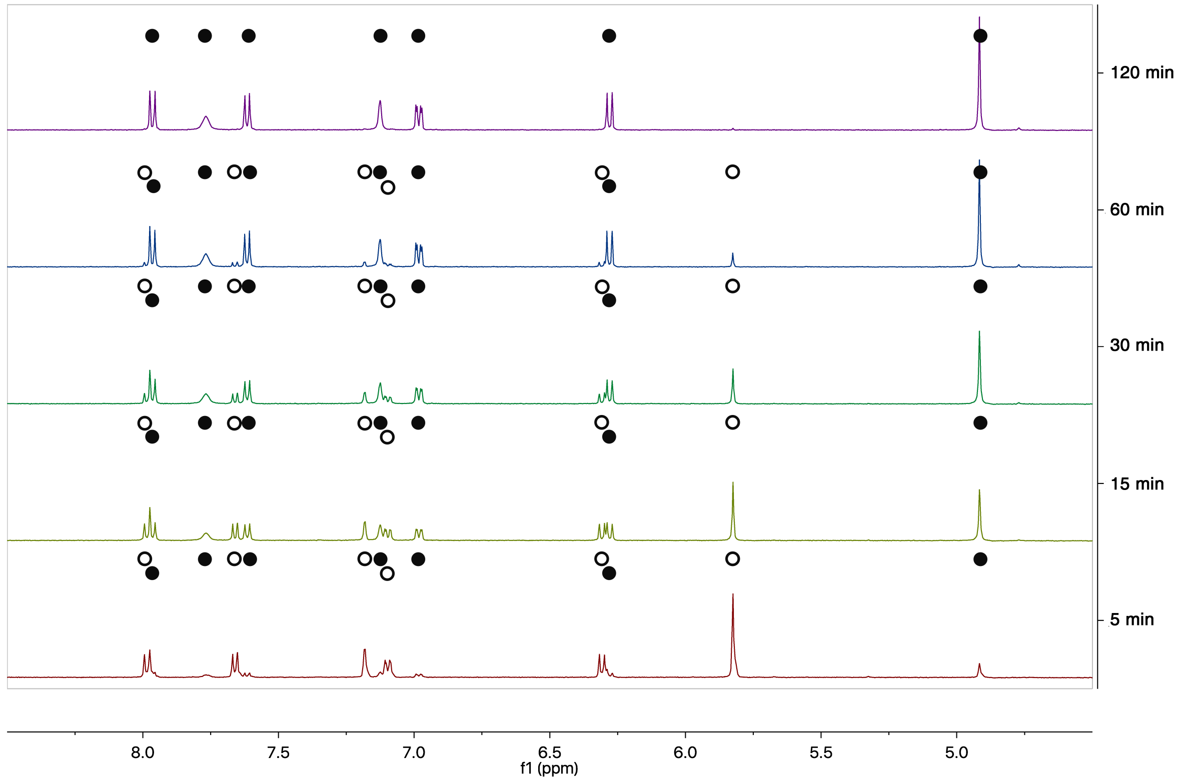


**Figure S29.** Focused time-dependent $^{1}$H-NMR spectra monitoring the reaction in DMSO-$d_6$:$D_2O$, 9:1, v/v.

## Computational Analyses

Computations were performed using Gaussian09 Rev D.01[4] on the Hoffman2 cluster of the Institute for Digital Research and Education at UCLA.

The M06-2X[5] density functional was used in combination with the def2-TZV basis set.[6] Grimme's D3 empirical dispersion was used included.[7] All calculations were performed in solution using water in the SMD model.[8] Stationary points were confirmed by frequency analyses where local minima showed no imaginary frequencies and transition states showed exactly one imaginary frequency. IRC calculations were performed to link transition states to local minima. Conformer searched were performed using Maestro (Schrödinger). Quasiharmonic correction was applied to the frequency analysis by setting all frequencies below 100 cm-1 to 100 cm-1 using the Goodvibes script by Robert Paton.[9] Thermodynamic data is reported for 1 atm and 298.15 K. 3,6-di-methyl-1,2,5,6-tetrazine derived intermediates A1 and B1 were used as model substances and the n-Bu substituent of n-BuNC was exchanged against an ethyl group. Investigated mechanisms are shown in **Figure S30** and corresponding energies are listed in **Table S2**. Cartesian coordinates are provided at the end of the supporting information.

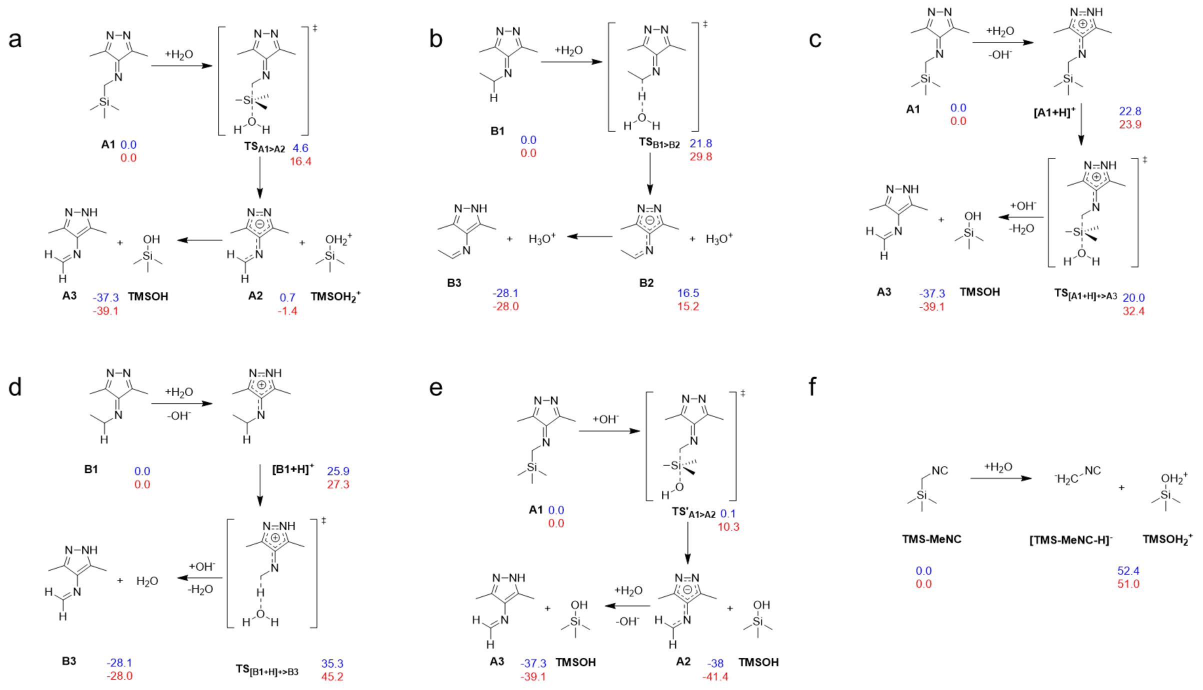


**Figure S30.** Investigated pathways. a) Cleavage of TMS from A1 by water with following protonation. b) Tautomerization of B1 induced by abstraction of a proton by water. c) Cleavage of TMS from A1 by protonation followed by attack of water. d) Tautomerization of B1 induced by protonation. e) Cleavage of TMS from A1 by hydroxide with following protonation. f) Cleavage of TMS from TMS-MeNC by water.

**Table S2.** M06-2X-D3/def2-TZV(SMD) calculated energies. All values in hartree. qh=quasi harmonic corrected.

| | ΔE | ZPE | ΔH | T*S | $T*S_{qh}$ | ΔG | $ΔG_{qh}$ |
|---|---|---|---|---|---|---|---|
| **$H_2O$** | -76.4116 | 0.020762 | -76.387 | 0.021433 | 0.021433 | -76.4085 | -76.4085 |
| **OH-** | -75.9228 | 0.008344 | -75.9112 | 0.019583 | 0.019583 | -75.9308 | -75.9308 |
| **$H_3O^+$** | -76.8299 | 0.034321 | -76.7916 | 0.023202 | 0.023202 | -76.8148 | -76.8148 |
| **TMSOH** | -485.018 | 0.125995 | -484.882 | 0.041796 | 0.041796 | -484.924 | -484.924 |
| **$TMSOH_2^+$** | -485.445 | 0.13935 | -485.296 | 0.042149 | 0.042149 | -485.338 | -485.338 |
| **A1** | -806.66 | 0.250981 | -806.391 | 0.060247 | 0.058617 | -806.451 | -806.45 |
| **A2** | -397.625 | 0.135579 | -397.479 | 0.043609 | 0.04295 | -397.523 | -397.522 |
| **A3** | -398.112 | 0.149447 | -397.953 | 0.043969 | 0.043529 | -397.997 | -397.996 |
| **B1** | -437.373 | 0.177554 | -437.184 | 0.046555 | 0.04644 | -437.231 | -437.373 |
| **B2** | -436.929 | 0.163669 | -436.754 | 0.047147 | 0.046648 | -436.801 | -436.929 |
| **B3** | -437.418 | 0.177902 | -437.229 | 0.046931 | 0.046723 | -437.276 | -437.275 |
| **$[A1+H]^+$** | -807.112 | 0.264074 | -806.83 | 0.061277 | 0.059261 | -806.892 | -806.89 |
| **$[B1+H]^+$** | -437.821 | 0.190984 | -437.618 | 0.046657 | 0.046539 | -437.665 | -437.665 |
| **$TS_{A1>A2}$** | -883.064 | 0.275018 | -882.769 | 0.065032 | 0.062867 | -882.834 | -882.832 |
| **$TS'_{A1>A2}$** | -882.582 | 0.26139 | -882.302 | 0.065074 | 0.062606 | -882.367 | -882.364 |
| **$TS_{B1>B2}$** | -513.75 | 0.195752 | -513.541 | 0.052209 | 0.051151 | -513.593 | -513.592 |
| **$TS_{[A1+H]+>A3}$** | -883.528 | 0.28821 | -883.219 | 0.067312 | 0.064924 | -883.287 | -883.284 |
| **$TS_{[B1+H]+>B3}$** | -514.217 | 0.209787 | -513.994 | 0.052145 | 0.051133 | -514.046 | -514.045 |
| **TMS-MeNC** | -541.242 | 0.148771 | -541.081 | 0.048787 | 0.047247 | -541.13 | -541.128 |
| **$[TMS-MeNC-H]^-$** | -132.124 | 0.0307 | -132.089 | 0.028183 | 0.028183 | -132.117 | -132.117 |

## Dependence of the Release Step on Water

We investigated the dependence of the release step on the inclusion of water by photospectrometric measurements performed in a multi-plate reader. The kinetics of the release of pNA from 4a triggered by TMS-MeNC in solutions with varying amounts of water was tested (**Fig. S31**). The kinetics experiment was performed analogous to as described in the previous section—"Photospectrometric Analysis of Uncaging Step." Water may be involved to elicit the elimination of leaving groups through several plausible pathways (**Fig. S32**).

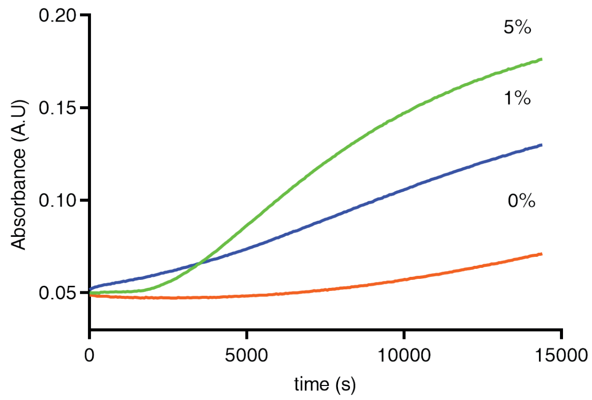


**Figure S31.** Kinetics of pNA release from 4a triggered by TMS-MeNC in increasing percentages of water content in DMSO (c(4a) = 0.2 mM, c(TMS-MeNC) = 2 mM, DMSO: PBS pH 7.4 (1:0 = Orange; 99:1 = Blue; 19:1 = Green), T = 37°C, λ = 435 nm).

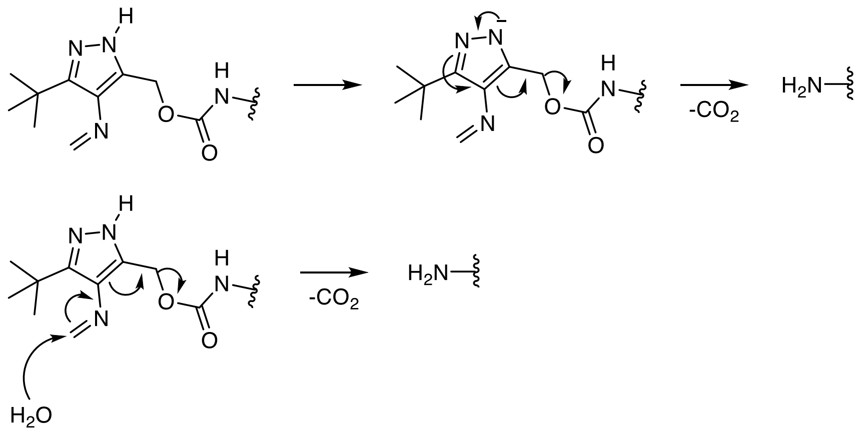


**Figure S32.** Plausible mechanisms for the elimination step. a) Elimination of the benzylic leaving group induced by deprotonation of the pyrazole. b) attack of the double bond by water and concerted electron migration leading to elimination of the leaving group.

## SNAP-protein In-Gel Fluorescence and Mass Spectroscopy

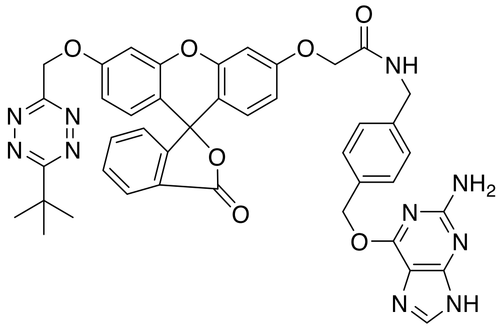

**Figure S33.** Structure of TzMe-caged O-carboxymethyl fluorescein-O$^6$-benzylguanine (4b-BG) used in this portion of the study

Purified SNAP-protein was obtained from New England BioLabs (NEB, USA, Catalog #P9312S). Labeling of the SNAP-protein was performed following the protocol provided by NEB ("Labeling SNAP-tag Purified Protein In Vitro," Protocol #P9312). In brief, the SNAP-protein was thawed and brought to room temperature before use. A 50 µL reaction mixture was prepared consisting of 1 mM DTT, 5 µM SNAP-protein, and 10 µM 4b-BG (**Fig. S33**) and allowed to react for 30 minutes in the dark at 37°C (final solution contains 4% DMSO in PBS pH 7.4). Subsequently, unreacted small molecules were removed by subjecting the reaction mixture through centrifugation with a 3 kDa MWCO filter. Proteins were re-suspended in PBS pH 7.4 (99.5 µL) and exposed to either vehicle (0.5 µL DMSO) or TMS-MeNC (0.5 µL of 20 mM stock for a final concentration of 100 µM) and incubated for another 2 hours at 37°C (final solution contains 0.5% DMSO). The solutions were then prepared for SDS-PAGE analysis and separated on a precast 12% BOLT Bis-Tris mini gel (ThermoFisher, USA) with MES running buffer. The protein ladder used was a PageRuler unstained low range standard ladder (ThermoFisher, USA). After electrophoresis, fluorescent proteins were detected using a GelDoc EZ Gel documentation system (Bio-Rad, USA) followed by fixing using a solution of acetic acid:MeOH:$H_2O$ (1:4:5) for 30 minutes. Finally, the gel was stained with Blue-Clean protein stain (IBI Scientific, USA) following manufacturer protocols, de-stained overnight, and imaged the following day with a GelDoc EZ Gel documentation system (Bio-Rad, USA). Expanded views of the gel images and a combined image are presented (**Fig. S34**).

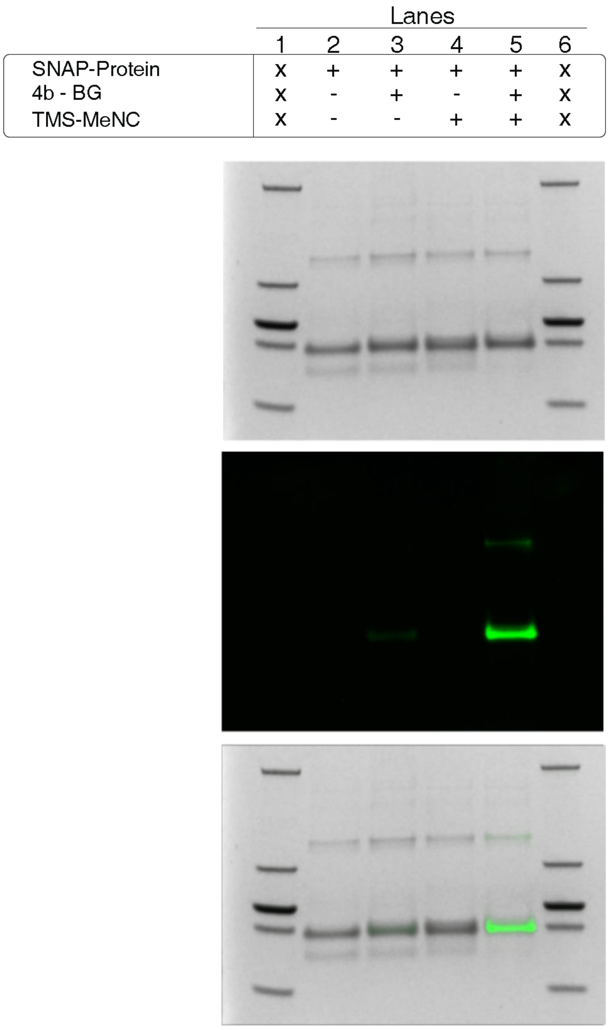


**Figure S34.** Expanded view of the Coomassie stained gel, fluorescent gel, and an overlay of the fluorescent gel over the Coomassie stained gel; lanes 1 and 6 contain the protein ladder. The fluorescence intensity is measured to be 11-fold greater in lane 5 compared to in lane 3 as measured by the histogram (GelDoc EZ Gel documentation system, Bio-Rad, USA).

To confirm the conjugation of 4b-BG to the purified SNAP protein (Lane 3, **Fig. S34**) and to evaluate the amount of TzMe removal from the SNAP protein-4b-BG construct when subjected to TMS-MeNC (Lane 5, **Fig. S34**), LCMS intact mass spectroscopy analysis was performed. In brief, reverse-phase LCMS was performed on an Eksigent Ekspert nanoLC 425 system (SciEx, USA) coupled to a Bruker MAXIS ETD II QToF mass spectrometer. Samples were prepared and diluted with water containing 0.1% formic acid and 5 µL were injected. A gradient consisting of buffer A (0.2% formic acid in water) and buffer B (0.2% formic acid in acetonitrile) was used at a flow rate of 10 µL/min starting at 15% buffer B to 95% over 53 minutes with a 10 cm long/ 1 mm inner diameter Waters Acquity UPLC BEH prewarmed column at 60°C. The purified SNAP protein (Lane 2, **Fig. S34**) from New England Biolabs is known to display two species with masses of 19694 m/z and 19829 m/z (with a C-terminal DTT moiety attached), which we have confirmed (**Fig. S35**). 4b-BG was confirmed to have been conjugated to the SNAP protein with masses of 20337 m/z being the addition of 4b-BG to the SNAP protein peak of 19694 m/z and 20472 m/z being the addition to the SNAP protein peak of 19829 m/z (**Fig. S36**) with, in the given 30 minute reaction period, an efficiency of loading ~30%. When exposed to TMS-MeNC, TzMe groups were effectively removed from the SNAP protein-4b-BG conjugate to obtain the fluorescent O-carboxymethyl fluorescein conjugated to the SNAP protein with masses of 20185 m/z being the removal of TzMe from the 20337 m/z conjugate and 20320 m/z being the removal of TzMe from the 20472 m/z conjugate (**Fig. S37**) with, in the given 2 hour reaction period, a deprotection of TzMe efficiency greater than 80%.

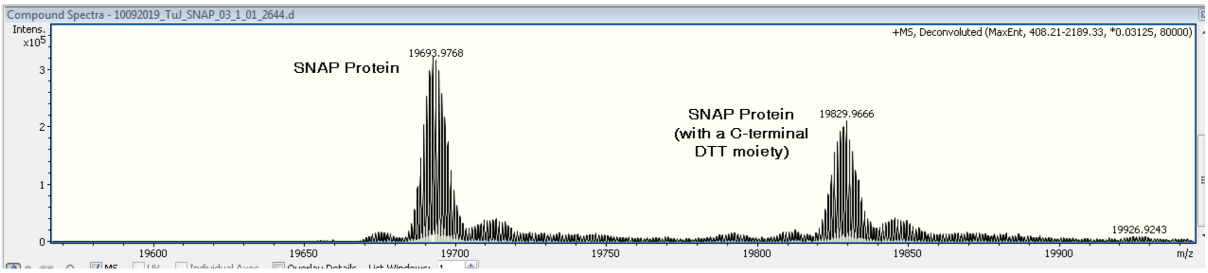


**Figure S35.** Zoomed in scan of the mass deconvolution of the purified SNAP protein standard.

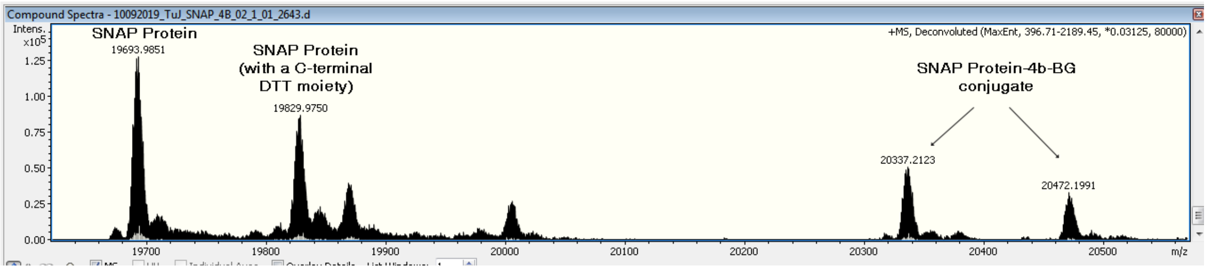


**Figure S36.** Zoomed in scan of the mass deconvolution of the reaction between the SNAP protein and our 4b-BG (Fig. S33) probe showing successful loading.

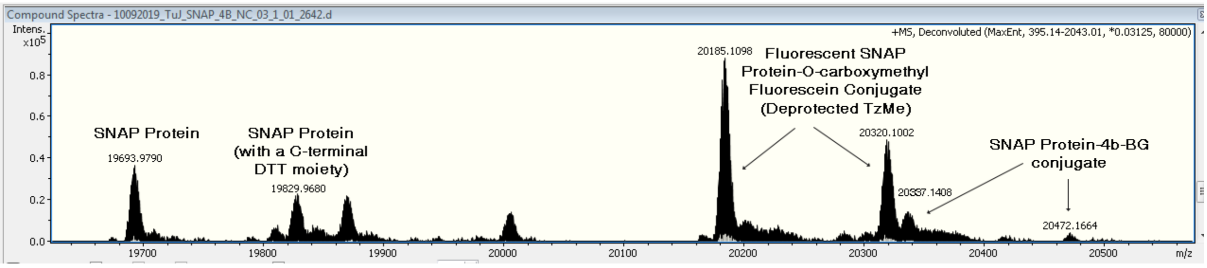


**Figure S37.** Zoomed in scan of the mass deconvolution of the reaction between the SNAP protein-4b-BG conjugate and TMS-MeNC showing greater than 80% removal of the TzMe-protecting group, revealing O-carboxymethyl fluorescein, giving the SNAP protein a fluorescence signal (Fig. S34).

.

## Cytotoxicity Assay

A549 lung adenocarcinoma cells (ATCC, USA) were maintained in a humidified $CO_2$ (5%) incubator at 37°C in RPMI (Thermo Fisher, USA) supplemented with 10% fetal bovine serum in the presence of 1% Penicillin-Streptomycin-Glutamine (Thermo Fisher, USA) and 0.2% Normocin (InvivoGen, USA). The cells were plated in 96-well TC treated plates (PerkinElmer, USA) at a 6,500 cells/well density prior to the experiment. For samples assessing the reaction-induced drug release, the Tzmoc-Dox prodrug (5) was added to the cells first (100 µL final volume per well) in a series of final concentrations ranging from 0.001 to 10 µM followed by addition of TMS-MeNC (100 µM). Doxorubicin was used as the positive control at the same concentrations. TMS-MeNC and Tzmoc-Dox alone were also tested with the same series of concentrations ranging from 0.01 to 100 µM to obtain their $EC_{50}$ values as negative controls. After a 72-hour incubation period at 37°C, cell viability was assessed by a CellTiter-Glo® assay (Promega, USA). After 15 min incubation at 25°C, the luminescence value was obtained on an Envision 2104 Multilabel Reader (PerkinElmer, USA). The proliferation assay was performed in triplicate. $EC_{50}$ values (**Table S3**) were derived from the normalized cell growth and corresponding sigmoidal curves were fitted and generated with GraphPad Prism 8.0.

**Table S3.** $EC_{50}$ values for compounds tested in the cell viability assay obtained through curve fitting with GraphPad Prism 8.0.

| **Compound** | **$EC_{50}$ (µM)** |
|---|---|
| Tzmoc-Dox (5) | >100 |
| TMS-MeNC | >100 |
| Doxorubicin | 0.202 ± 0.025 |
| Tzmoc-Dox (5) + TMS-MeNC (100 µM) | 0.239 ± 0.014 |

## Zebrafish Imaging

All zebrafish experiments were approved by the University of Utah Institutional Animal Care and Use Committee. Zebrafish were maintained using standard husbandry protocols. Embryos were collected after mating wild-type TuAB zebrafish strain. 24 hours post fertilization (hpf), zebrafish larvae were treated with 0.003% Phenyl thiourea (PTU) in E3 media to inhibit melanin pigmentation. Another 24 hours later (48 hpf), larvae were manually dechorinated and anesthetized in Tricaine. 1-2 nL of 1mM 4b (prepared in TAE buffer) was injected in the yolk sac of the larvae. Non-injected zebrafish larvae or those injected with 1mM 4b were then split in 24-well plates (6 larvae/well) and treated with indicated concentrations of ICPr-rsf or TMS-MeNC in E3 media for 2 hours. Subsequently, larvae were washed three times with E3 media before imaging using an epi-fluorescent microscope (Zeiss SteREO Discovery.V8 microscope) fitted with a PentaFluar S 120 vertical illuminator and coupled with an Xcite Series 120PC light source. Fluorescence intensity quantitation was performed using ImageJ. Briefly, the freehand selection tool was used to select the entire zebrafish yolk sac. The total red (resorufin) and green (fluorescein) fluorescence in the yolk sac of zebrafish was measured. For data analysis, the fluorescence intensity in the yolk sac of non-injected zebrafish was subtracted from zebrafish injected with 4b and treated under the same conditions. An unpaired t-test (GraphPad Prism 8.0) was used for comparison between the DMSO-treated sample and those treated with TMS-MeNC or ICPr-rsf. The data are presented as Mean +/- SEM. 6-10 larvae were imaged and analyzed per condition (**Fig. S38**).

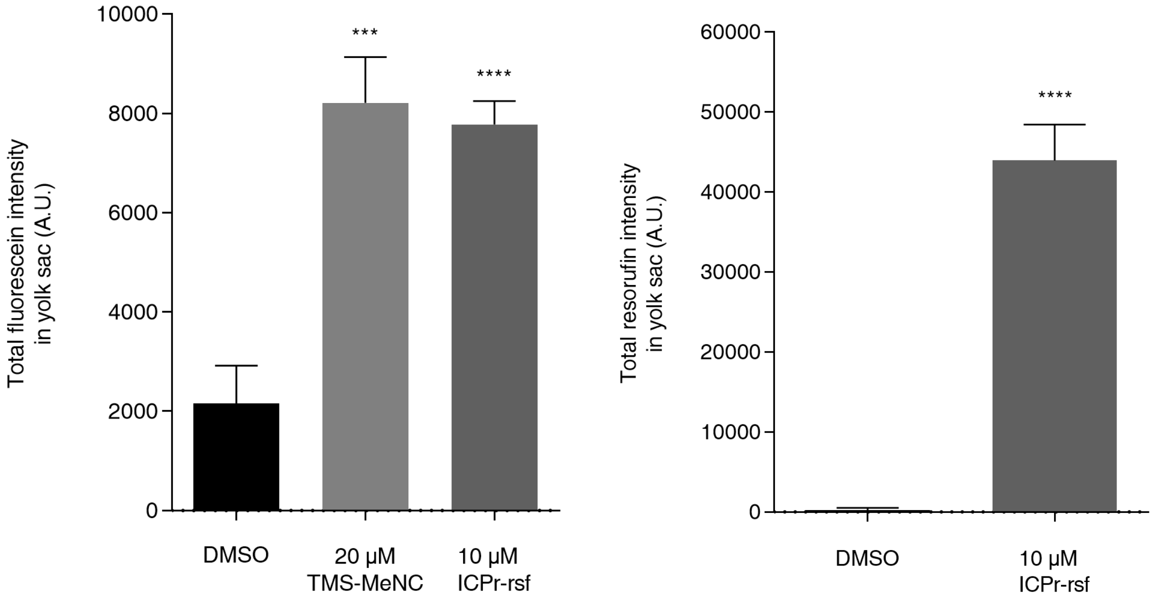


**Figure S38.** Quantification of fluorescence turn-on in zebrafish embryo for fluorescein (left) and resorufin (right); t = 2 h; *** $p \leq 0.001$; **** $p \leq 0.0001$.

## Dual Release of TzMe- and ICPr- caged Molecules

The dual release of two orthogonal fluorophores, resorufin and O-carboxymethyl-fluorescein, caged by previously disclosed 3-isocyanopropyl (ICPr) chemistry[3] and TzMe chemistry described here, respectively, was monitored by HPLC. In brief, 4b (0.5 mM) was incubated with ICPr-rsf (1 mM) and aliquots were subsequently taken out and diluted in MeOH for analysis by HPLC; the relevant retention timeframes of 5 – 14 minutes is displayed (**Fig. S39**). For HPLC analysis, we used a LUNA C18 column (5 µM, 150×2.0 mm, Phenomenex, USA) with a mobile phase consisting of A (0.1% TFA in water) and B (acetonitrile) running a gradient from 0% to 75% component B over a 19 minute timeframe monitoring a single 480 nm wavelength channel.

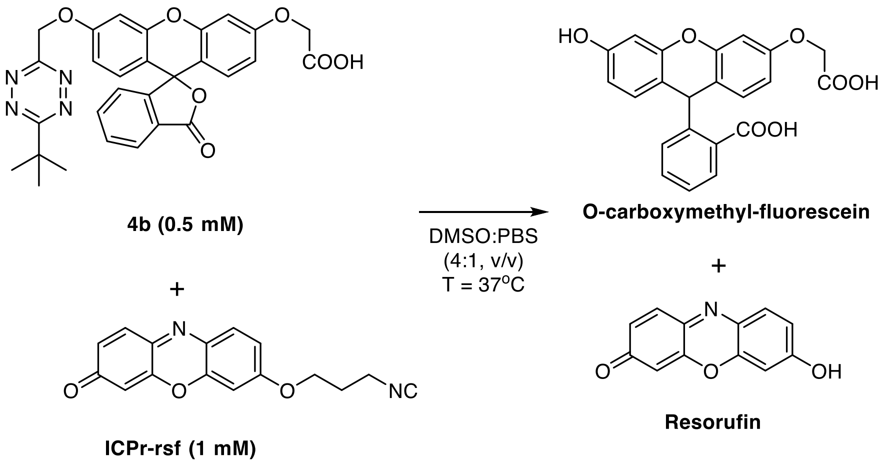


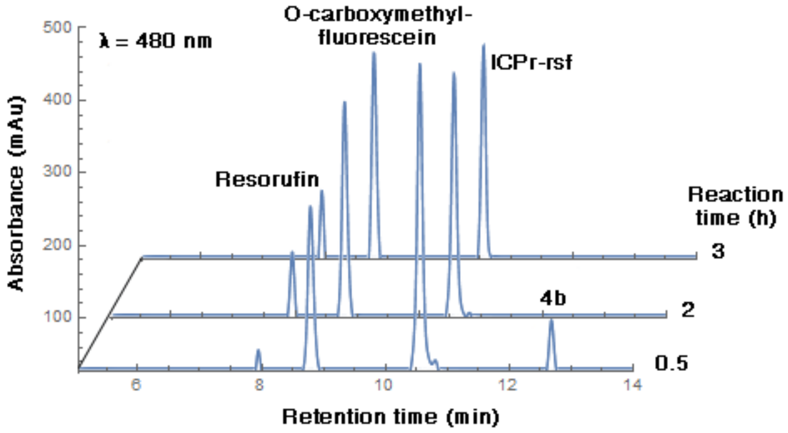


**Figure S39.** Reaction schematic for the dual release of O-carboxymethyl-fluorescein and resorufin and corresponding HPLC analysis of the dual release of O-carboxymethyl-fluorescein and resorufin; (c(4b) = 0.5 mM, c(ICPr-rsf) = 1 mM, DMSO: PBS pH 7.4 (4:1), T = 37°C, λ = 480 nm)

## References

[1] K. Utimoto, Y. Wakabayashi, Y. Shishiyama, M. Inoue and H. Nozaki, *Tetrahedron Lett.*, **1981**, 22, 4279-4280.

[2] J. Cai, X. Li, X. Yue and J. S. Taylor, *J. Am. Chem. Soc.*, **2004**, 126, 16324-16325.

[3] J. Tu, M. Xu, S. Parvez, R. T. Peterson and R. M. Franzini, *J. Am. Chem. Soc.*, **2018**, 140, 8410-8414.

[4] Frisch, M. J.; Trucks, G. W.; Schlegel, H. B.; Scuseria, G. E.; Robb, M. A.; Cheeseman, J. R.; Scalmani, G.; Barone, V.; Mennucci, B.; Petersson, G. A.; Nakatsuji, H.; Caricato, M.; Li, X.; Hratchian, H. P.; Izmaylov, A. F.; Bloino, J.; Zheng, G.; Sonnenberg, J. L.; Hada, M.; Ehara, M.; Toyota, K.; Fukuda, R.; Hasegawa, J.; Ishida, M.; Nakajima, T.; Honda, Y.; Kitao, O.; Nakai, H.; Vreven, T.; Montgomery, J. A.; Peralta, J. E.; Ogliaro, F.; Bearpark, M.; Heyd, J. J.; Brothers, E.; Kudin, K. N.; Staroverov, V. N.; Kobayashi, R.; Normand, J.; Raghavachari, K.; Rendell, A.; Burant, J. C.; Iyengar, S. S.; Tomasi, J.; Cossi, M.; Rega, N.; Millam, J. M.; Klene, M.; Knox, J. E.; Cross, J. B.; Bakken, V.; Adamo, C.; Jaramillo, J.; Gomperts, R.; Stratmann, R. E.; Yazyev, O.; Austin, A. J.; Cammi, R.; Pomelli, C.; Ochterski, J. W.; Martin, R. L.; Morokuma, K.; Zakrzewski, V. G.; Voth, G. A.; Salvador, P.; Dannenberg, J. J.; Dapprich, S.; Daniels, A. D.; Farkas; Foresman, J. B.; Ortiz, J. V.; Cioslowski, J.; Fox, D. J.: Wallingford CT, **2009**.

[5] Y. Zhao, D. G. Truhlar, *Theor. Chem. Acc.* **2007**, 120, 215.

[6] F. Weigend, R. Ahlrichs, *Phys. Chem. Chem. Phys.* **2005**, 7, 3297.

[7] S. Grimme, J. Antony, S. Ehrlich, H. Krieg, *J. Chem. Phys.* **2010**, 132, 154104.

[8] A. V. Marenich, C. J. Cramer, D. G. Truhlar, *J. Phys. Chem. B* **2009**, 113, 6378.

[9] Ignacio Funes-Ardoiz, & Robert S. Paton. (2016, August 24). GoodVibes: GoodVibes v1.0.1 (Version 1.0.1). Zenodo. http://doi.org/10.5281/zenodo.60811

# NMR of Synthetic Compounds

*3-(tert-butyl)-6-(methoxymethyl)-1,2,4,5-tetrazine*

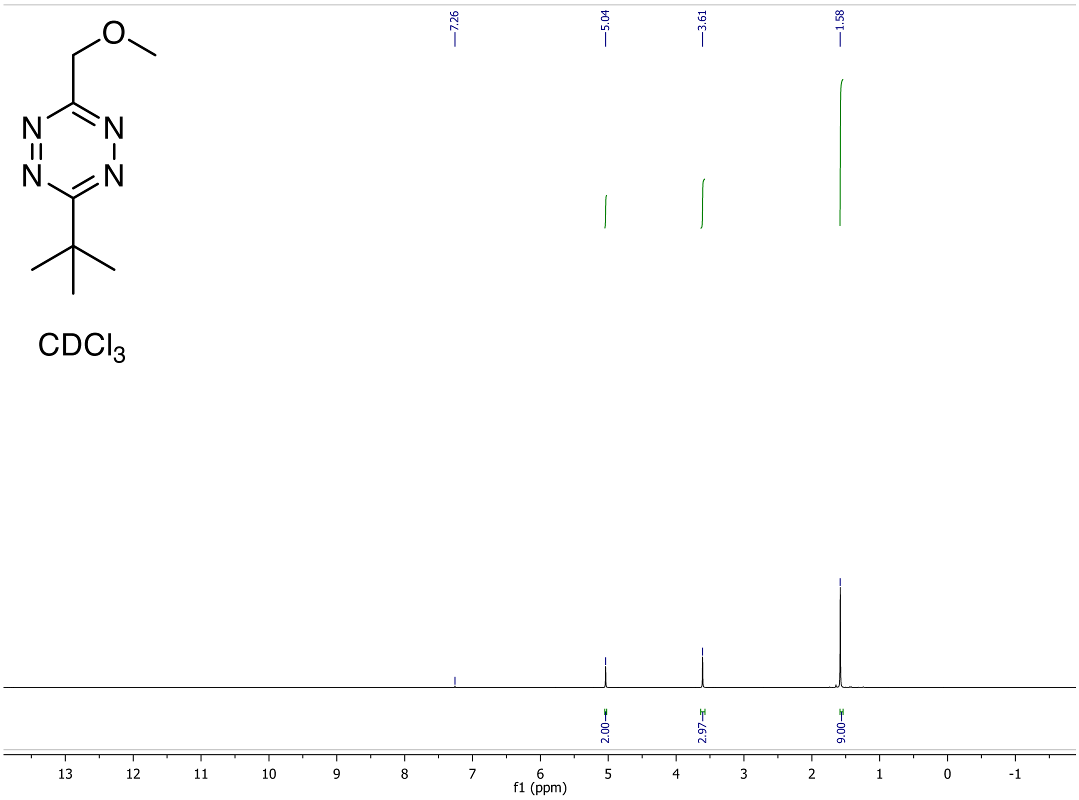


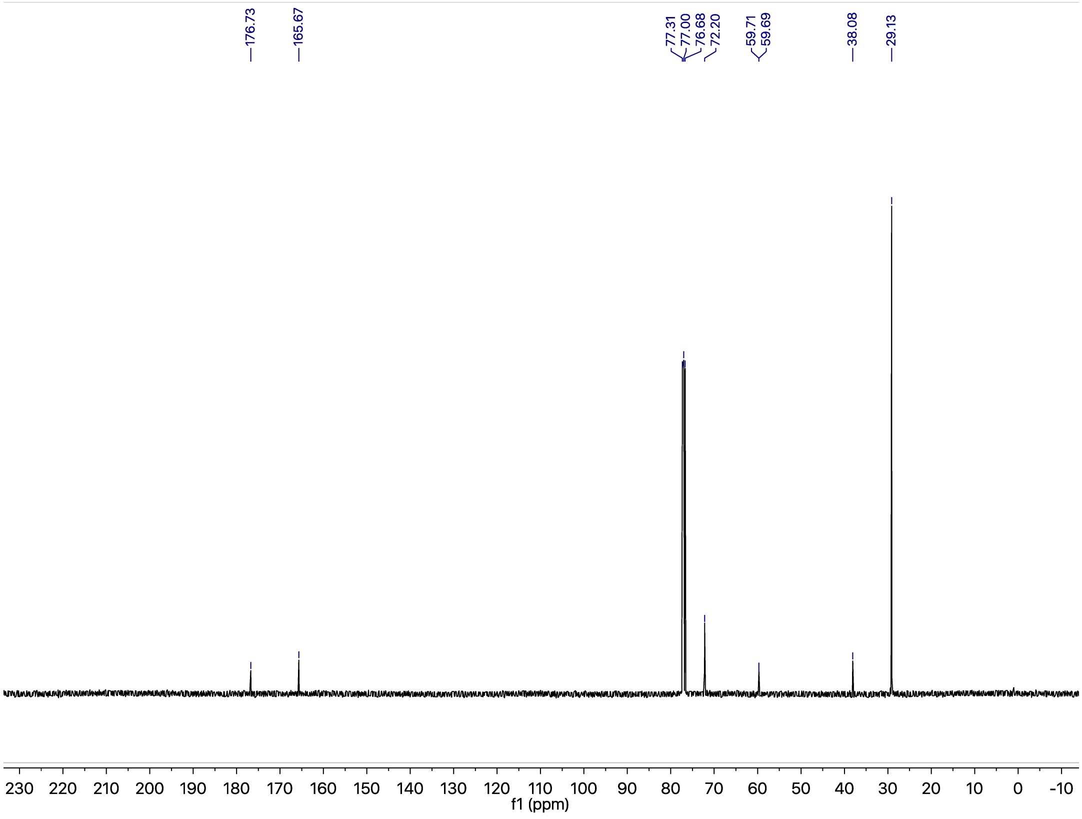

*2-methoxypropanenitrile*

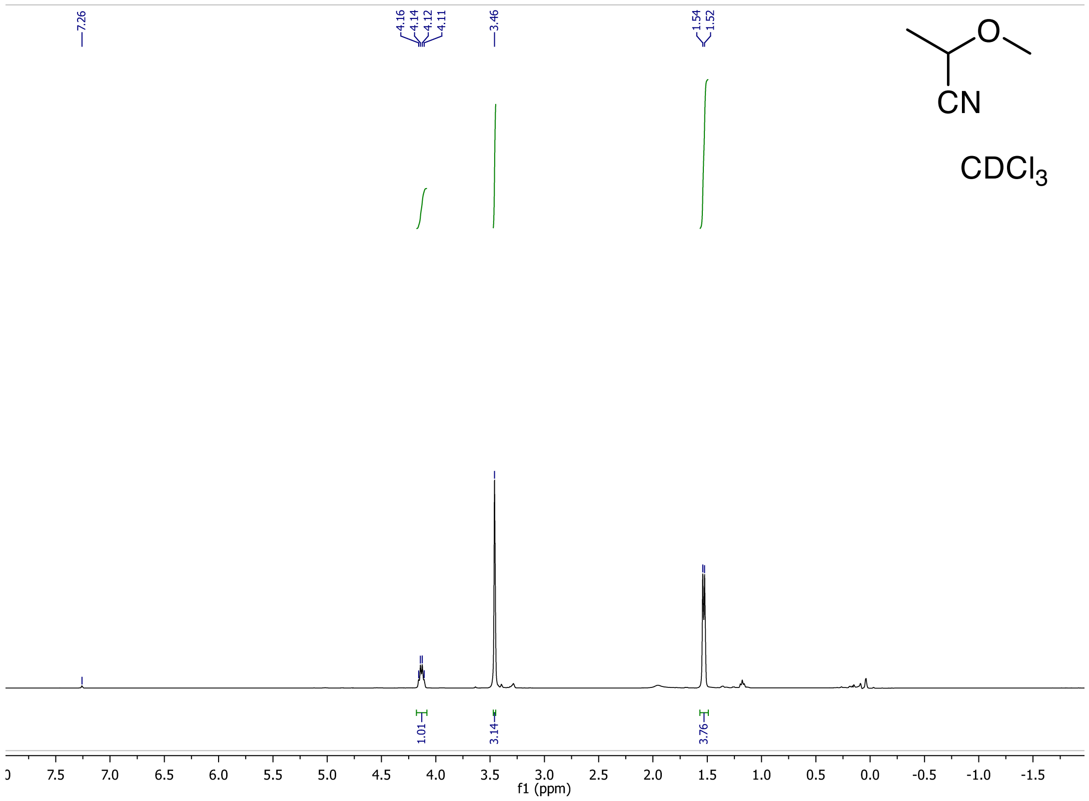


*2-methoxy-2-phenylacetonitrile*

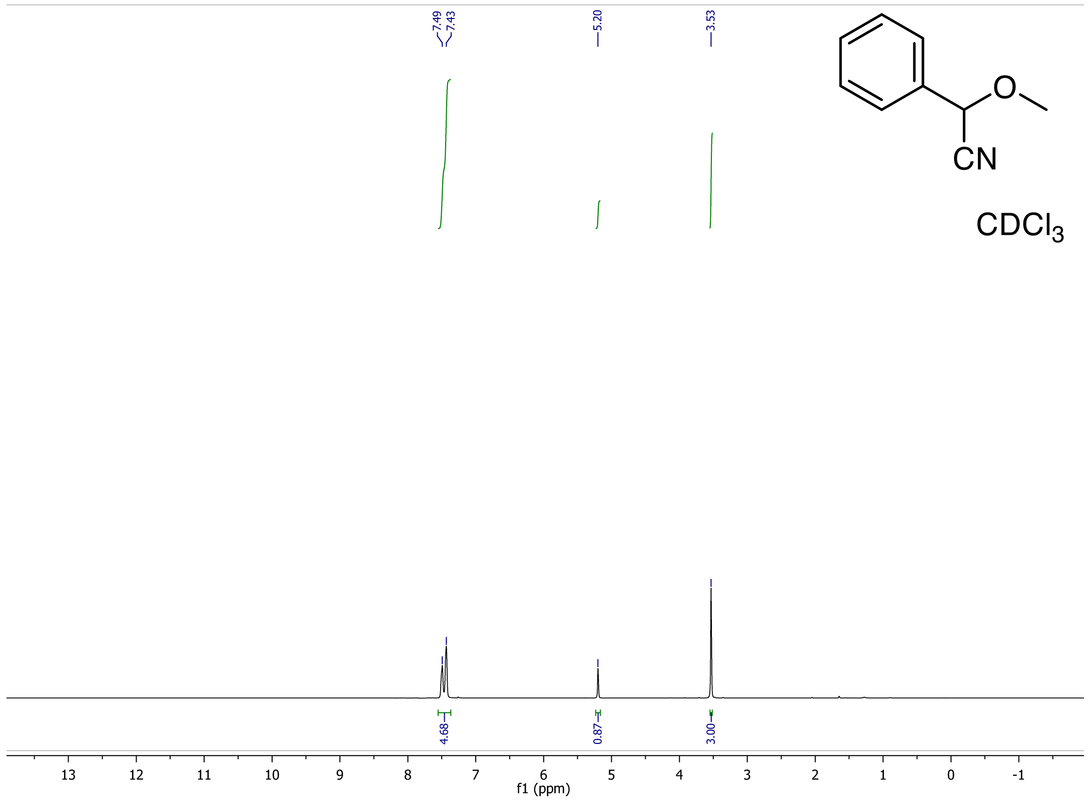

*3-(tert-butyl)-6-(1-methoxyethyl)-1,2,4,5-tetrazine*

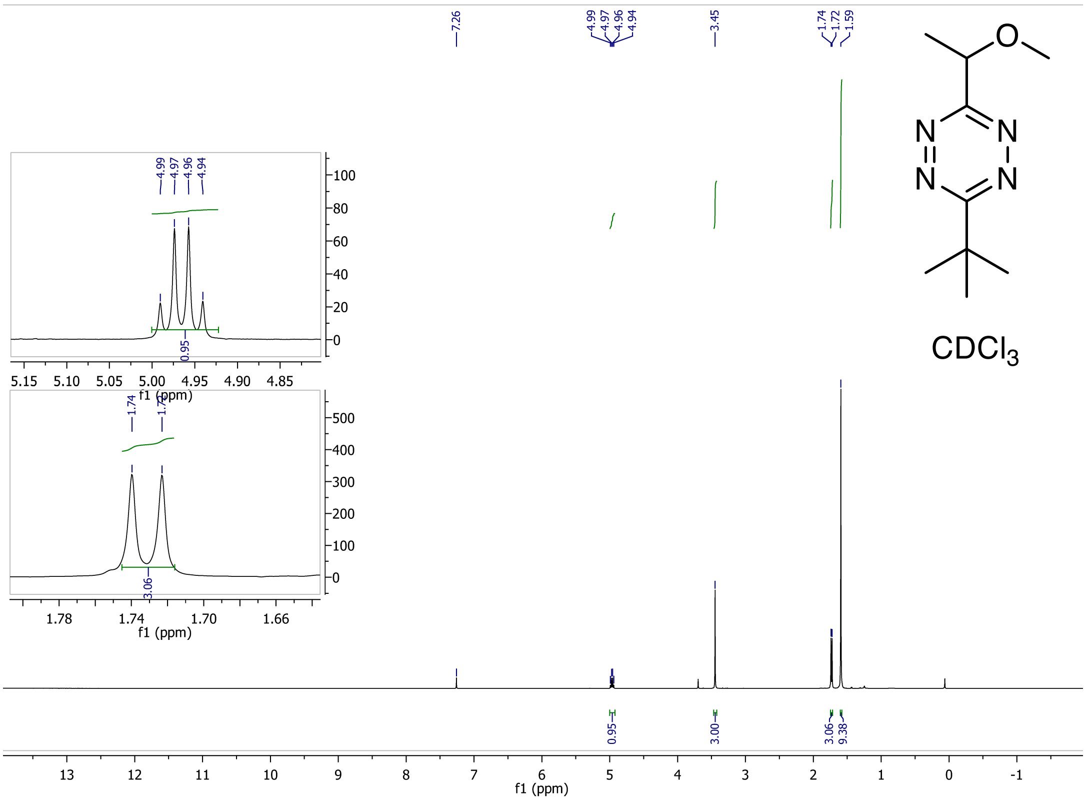


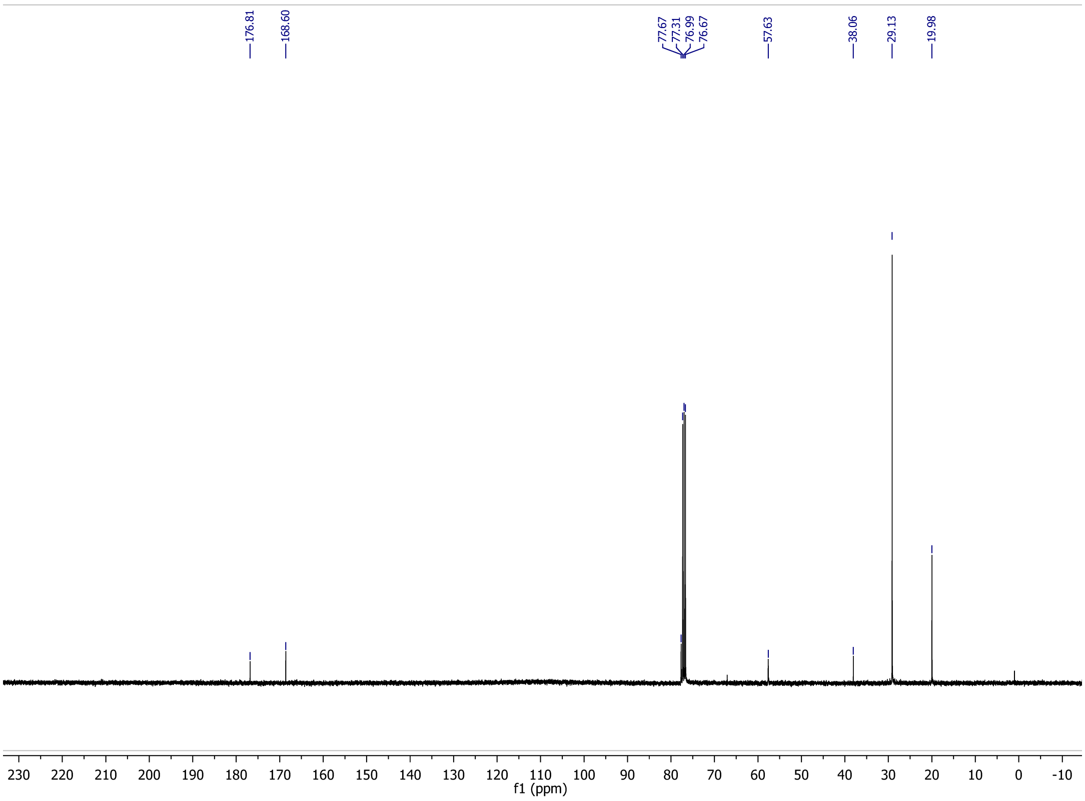

*3-(tert-butyl)-6-(methoxy(phenyl)methyl)-1,2,4,5-tetrazine*

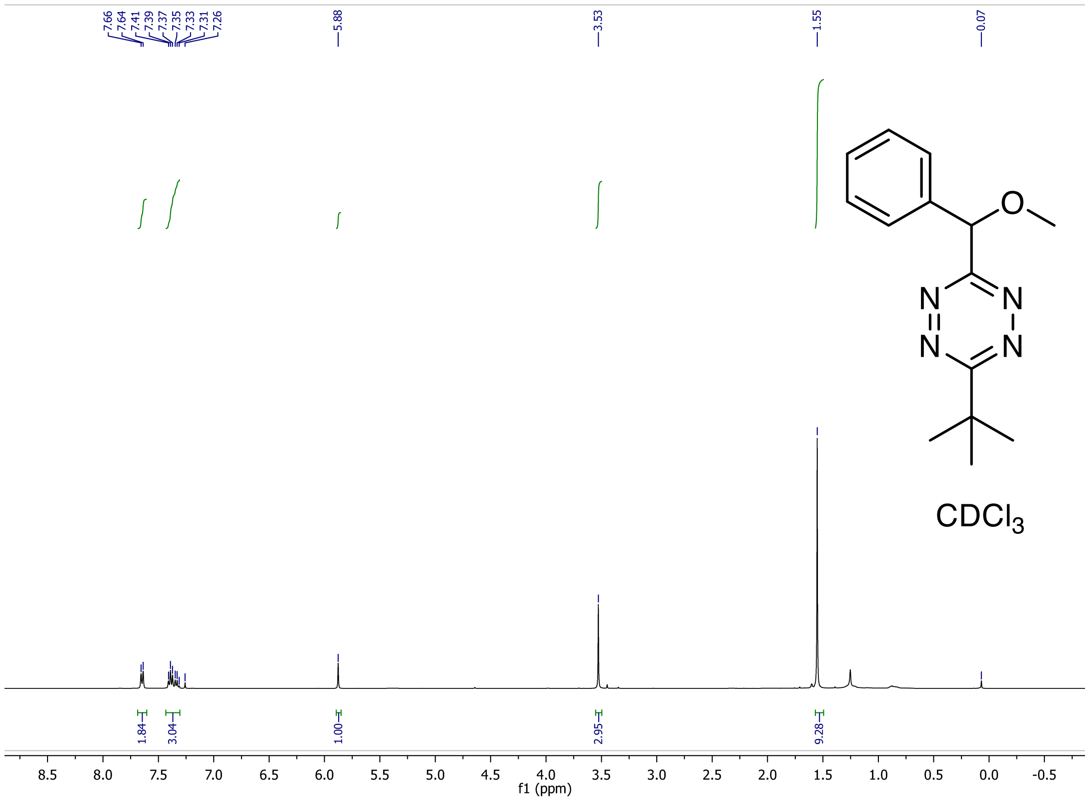


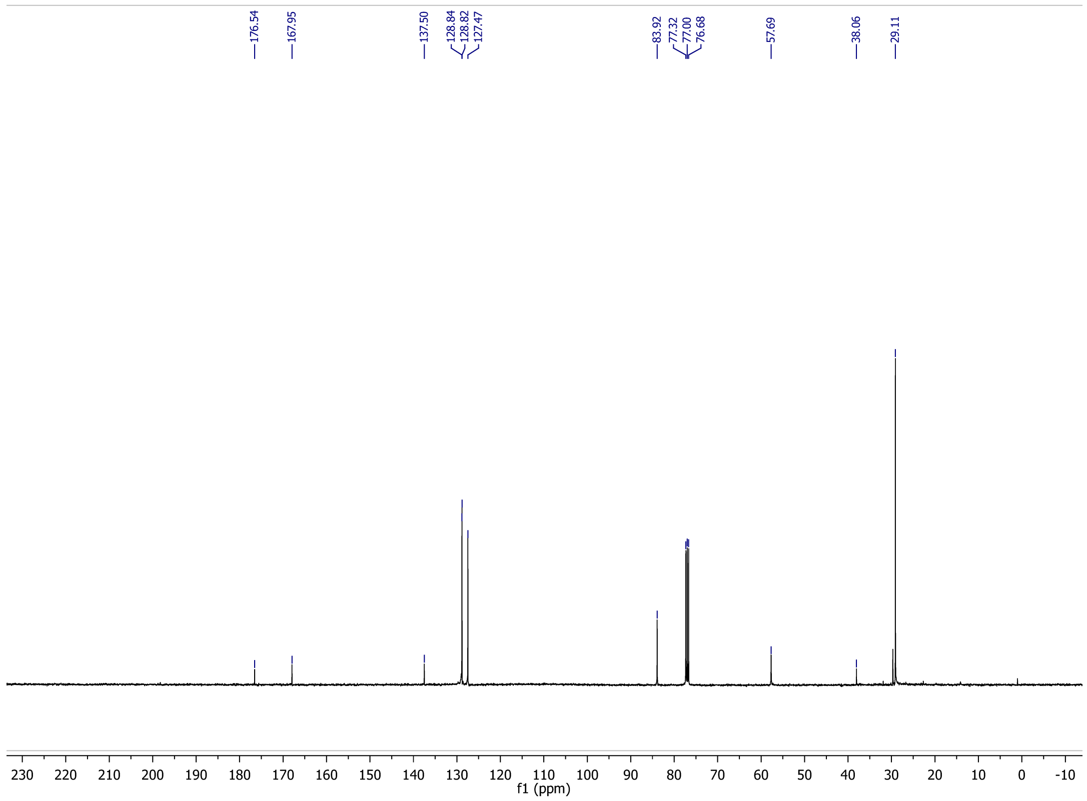

*3-(methoxymethyl)-6-phenyl-1,2,4,5-tetrazine*

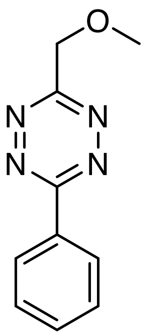


DMSO-$d_6$

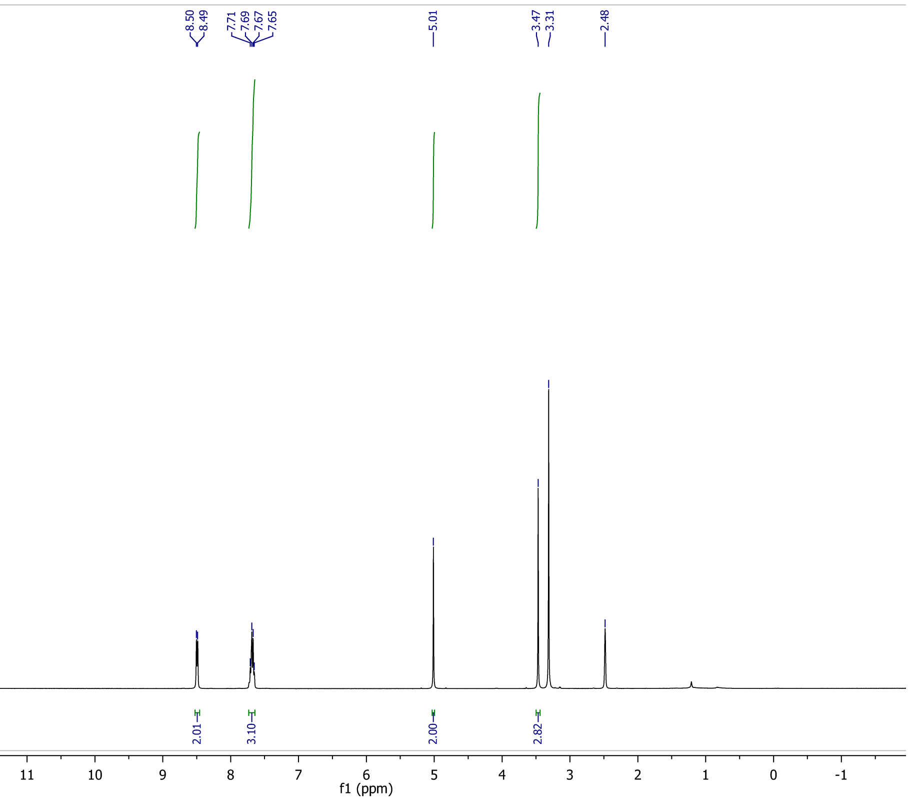


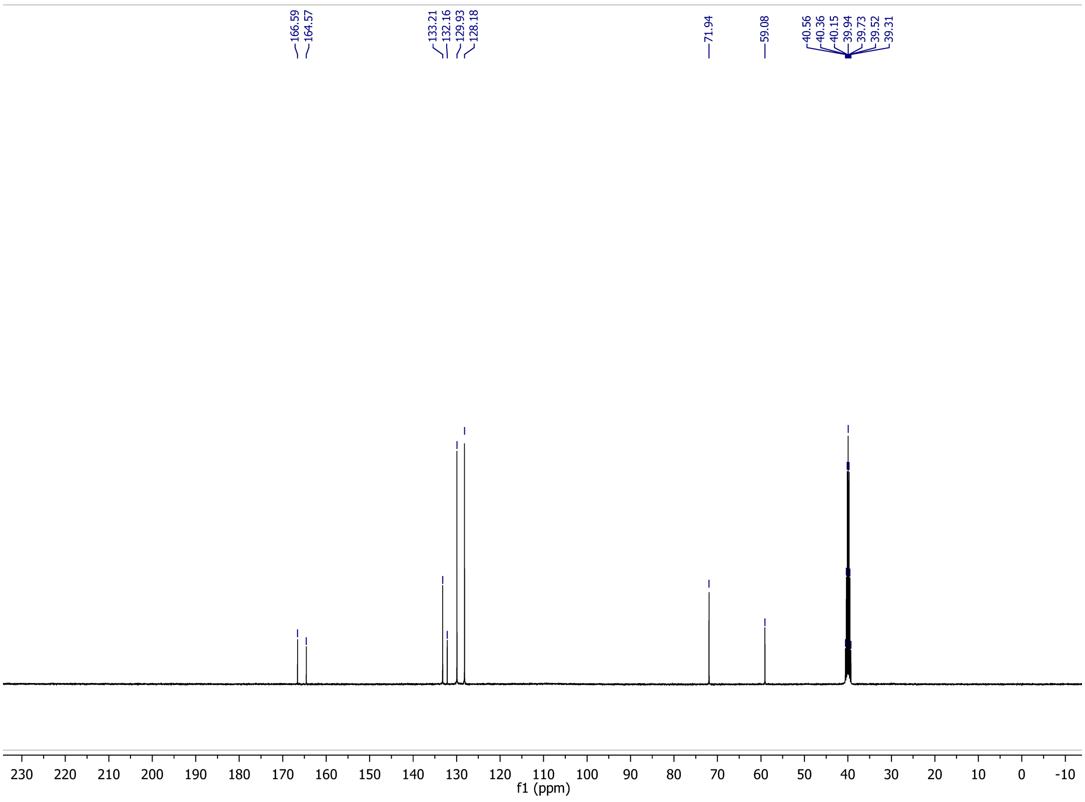

*(6-(tert-butyl)-1,2,4,5-tetrazin-3-yl)methanol*

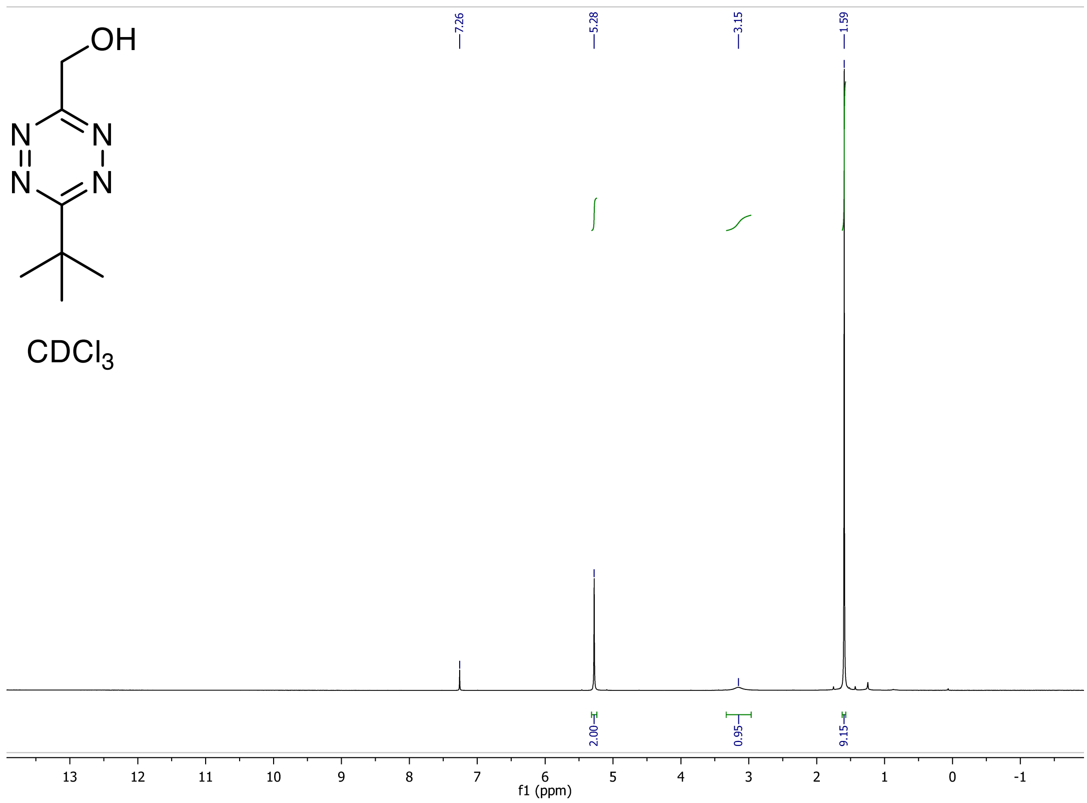


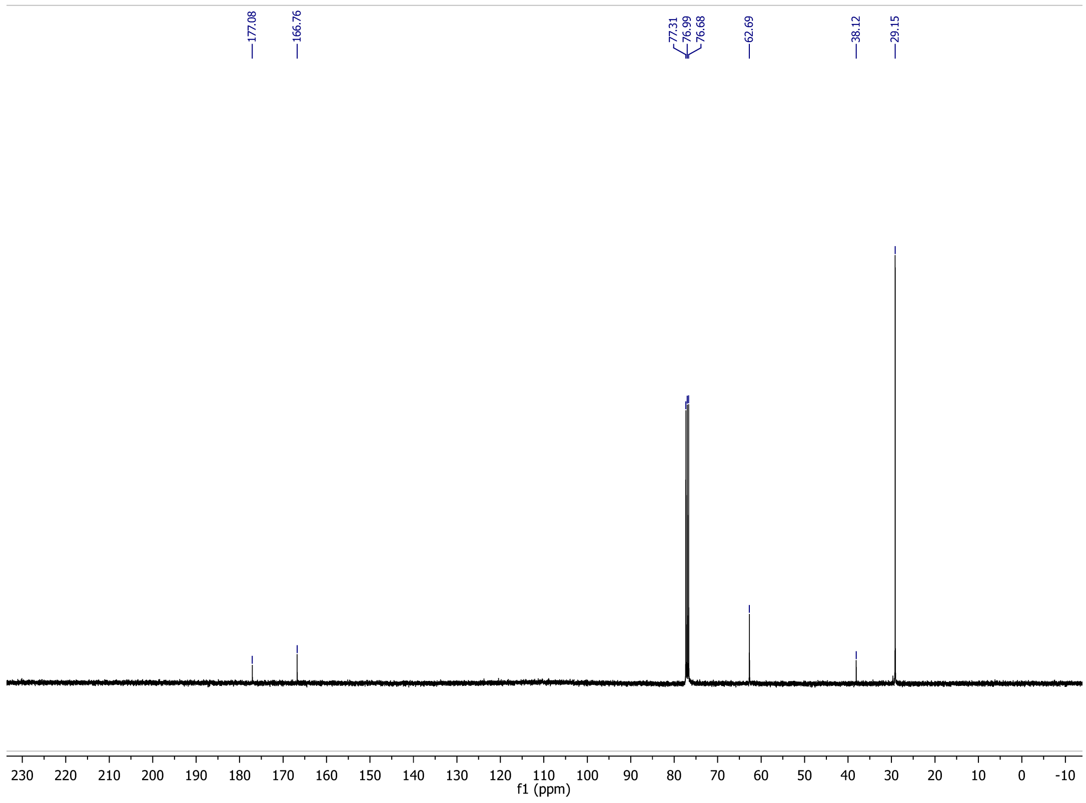

*1-(6-(tert-butyl)-1,2,4,5-tetrazin-3-yl)ethan-1-ol*

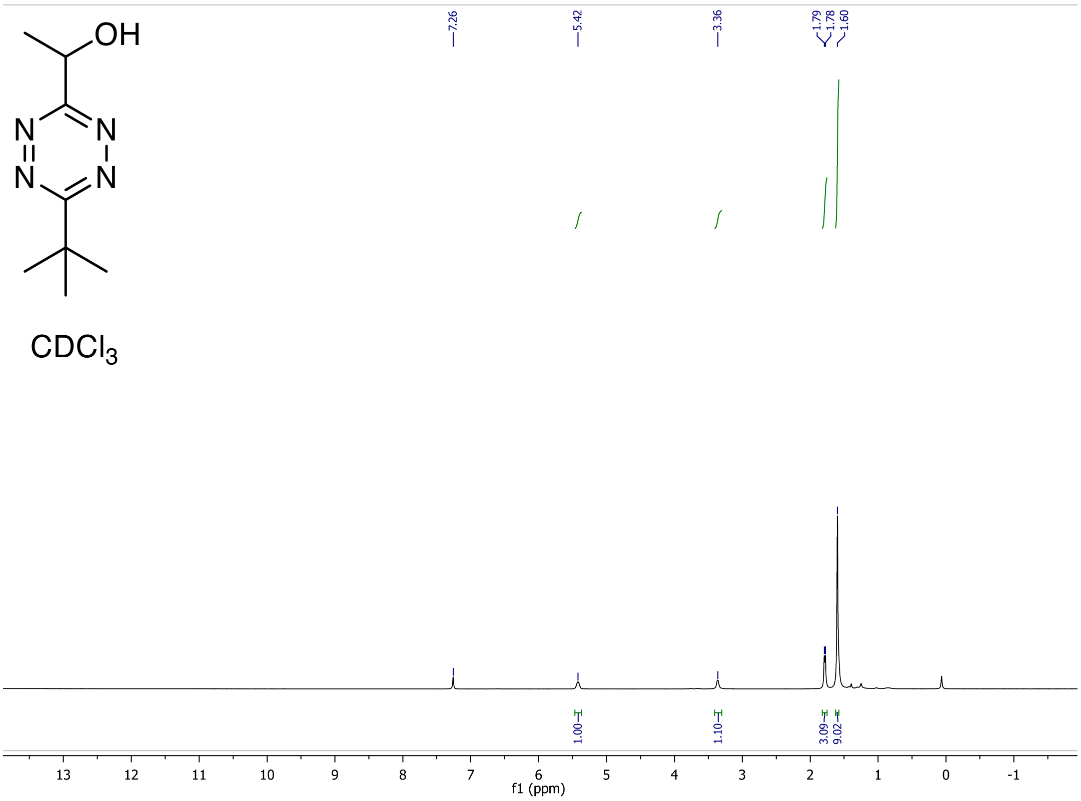


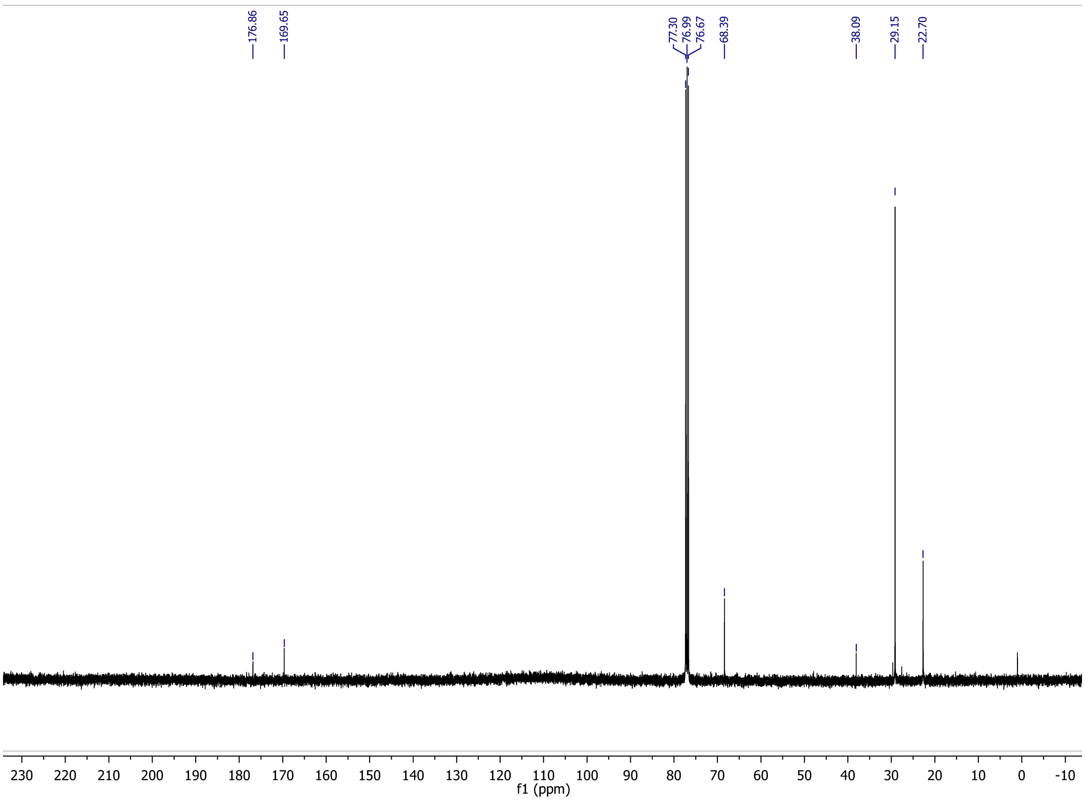

*(6-(tert-butyl)-1,2,4,5-tetrazin-3-yl)(phenyl)methanol*

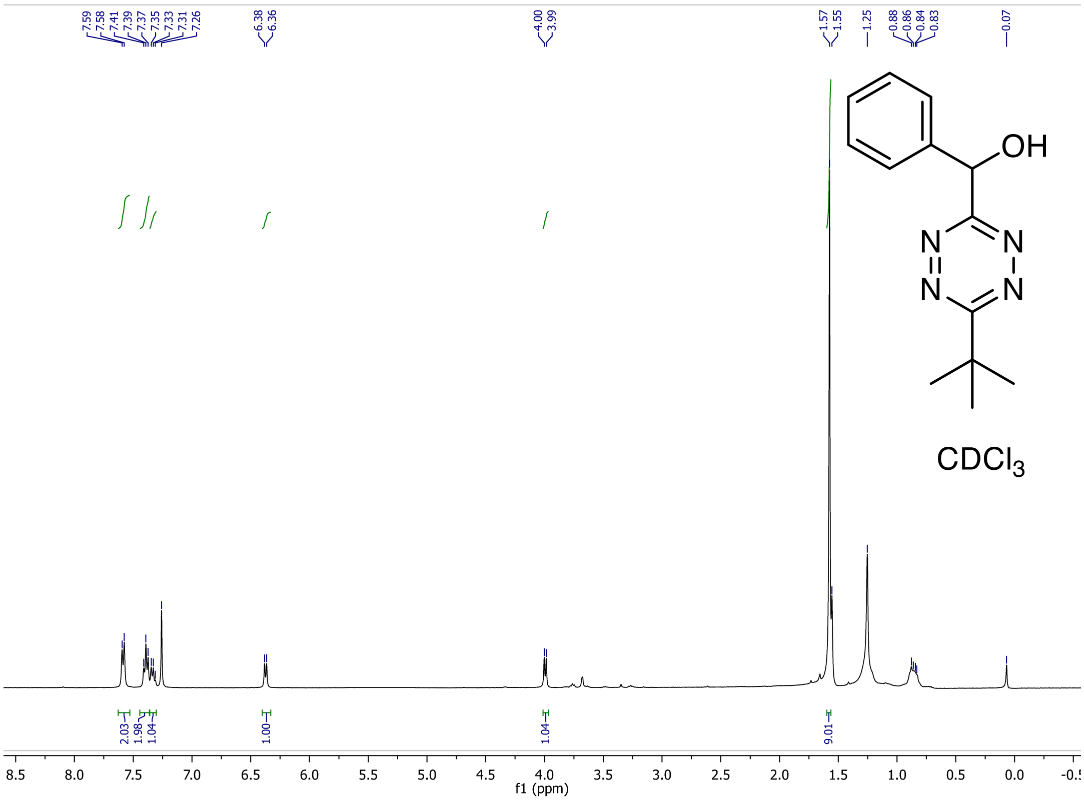


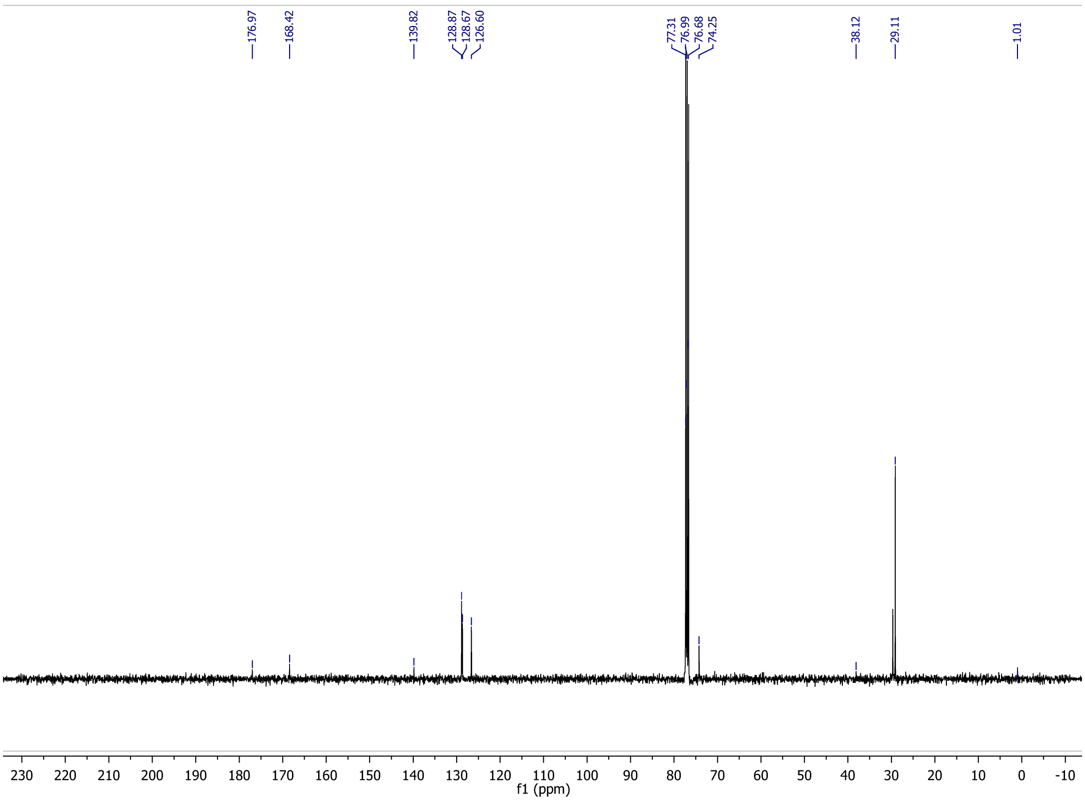

*(6-phenyl-1,2,4,5-tetrazin-3-yl)methanol*

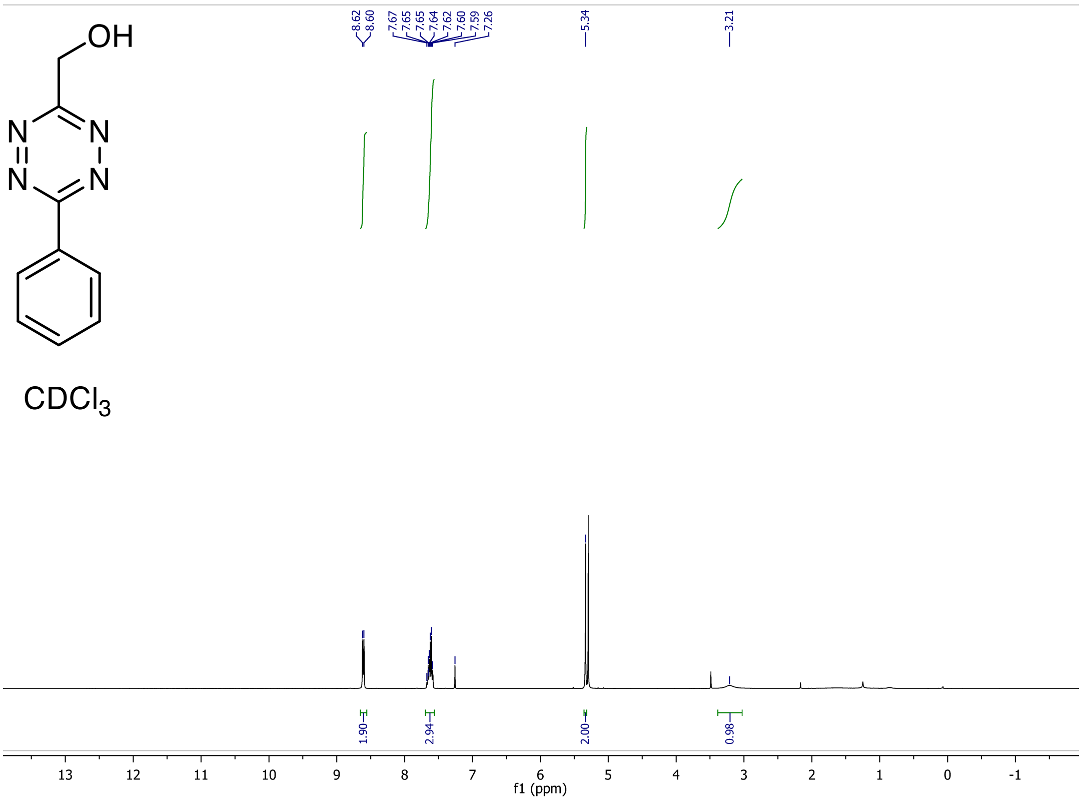


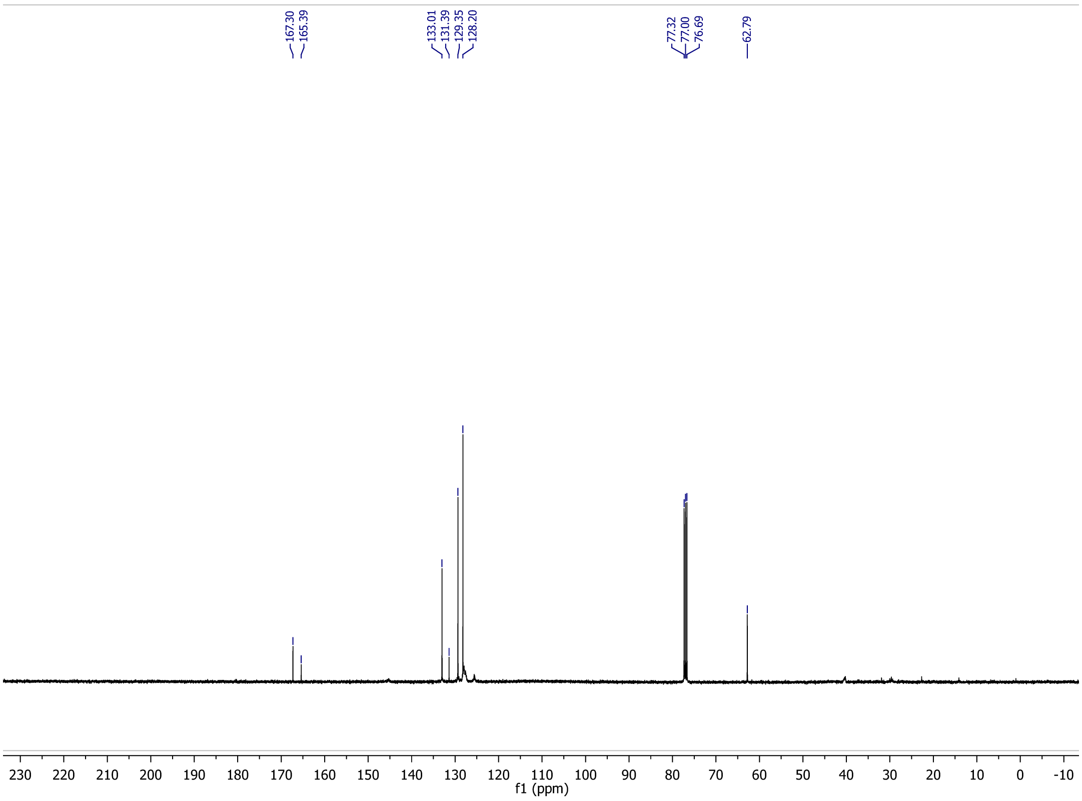

*(6-(tert-butyl)-1,2,4,5-tetrazin-3-yl)methyl (4-nitrophenyl)carbamate (**4a**)*

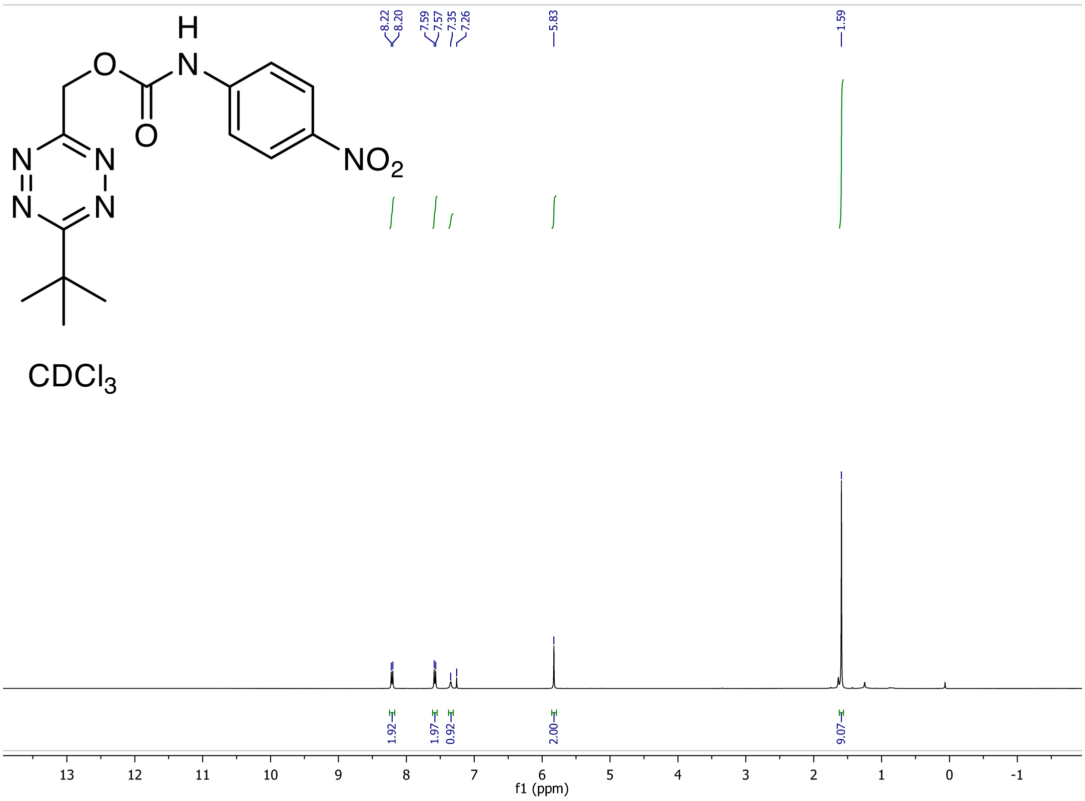


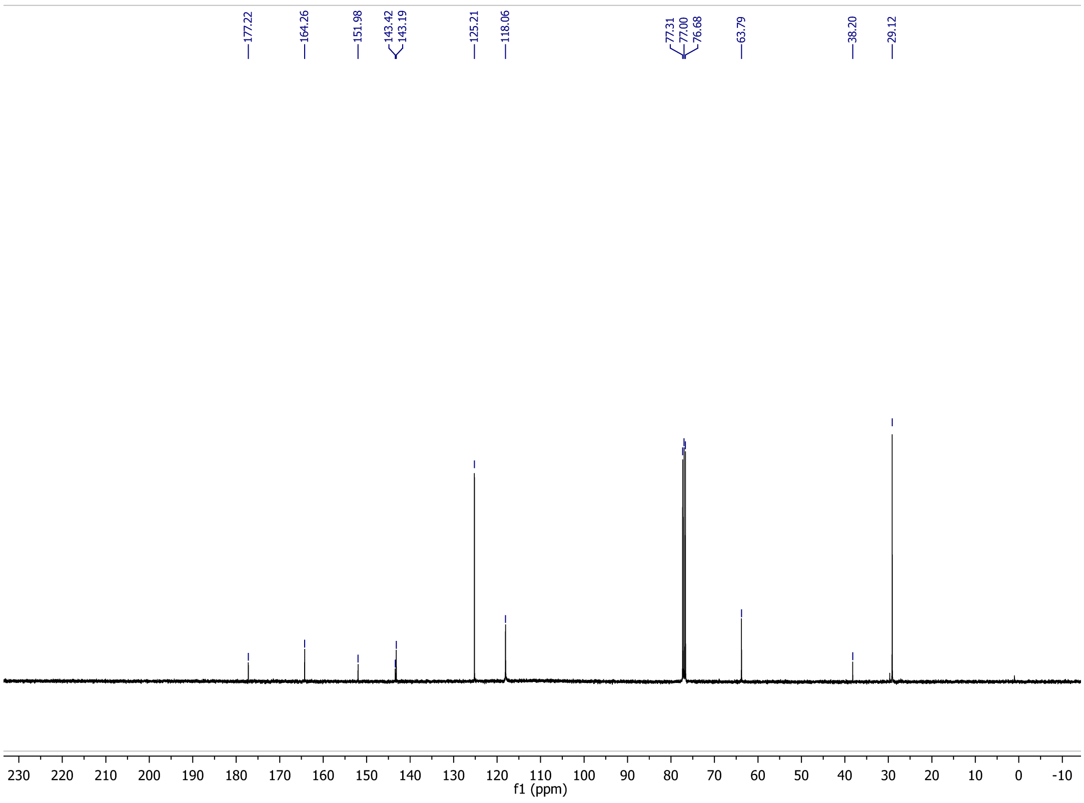

*1-(6-(tert-butyl)-1,2,4,5-tetrazin-3-yl)ethyl (4-nitrophenyl)carbamate (**4c**)*

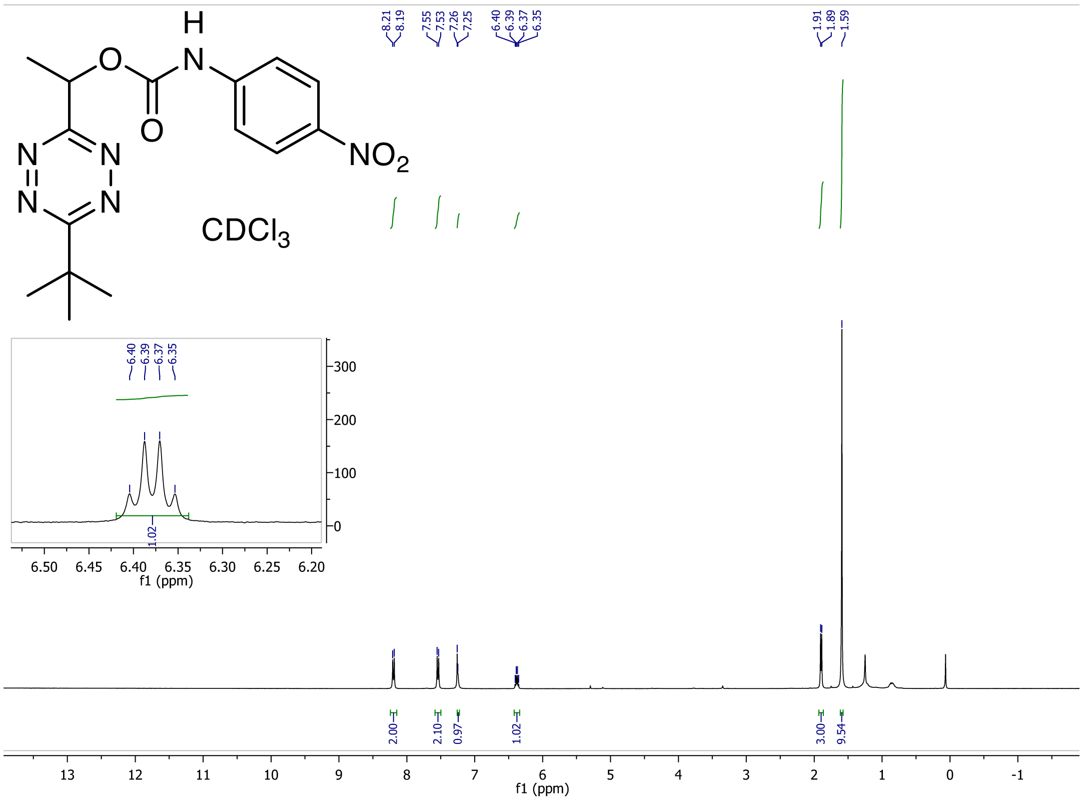


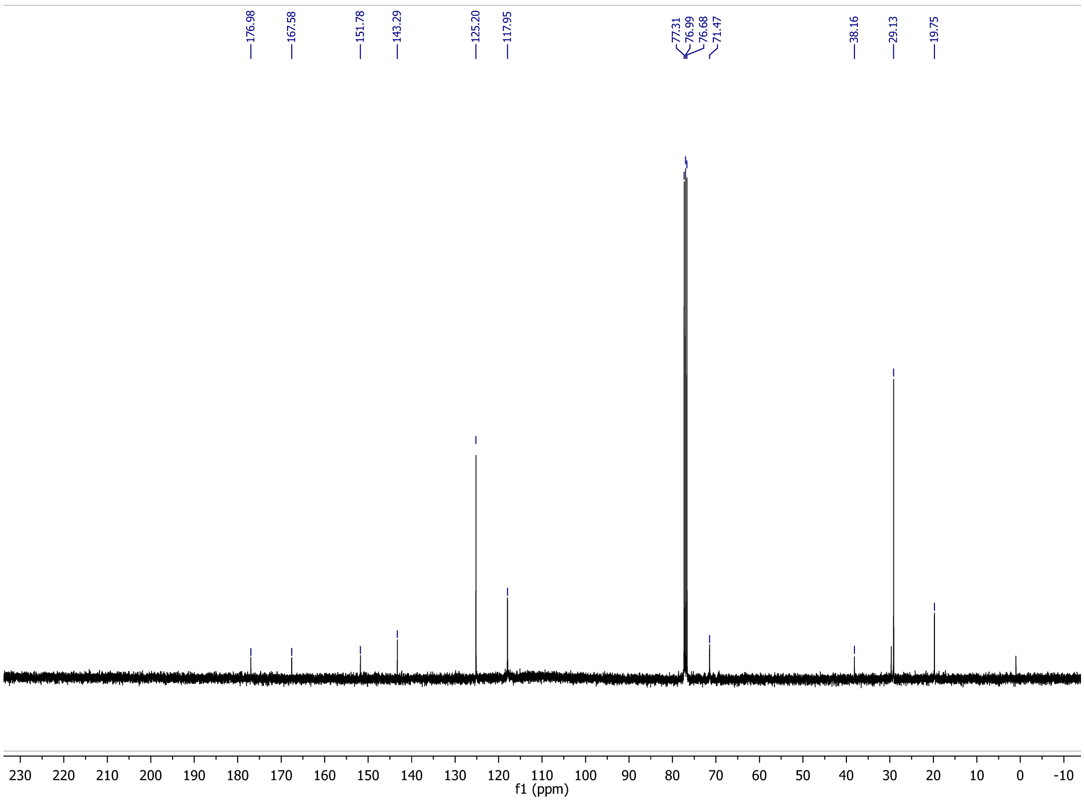

*(6-(tert-butyl)-1,2,4,5-tetrazin-3-yl)(phenyl)methyl (4-nitrophenyl)carbamate (**4d**)*

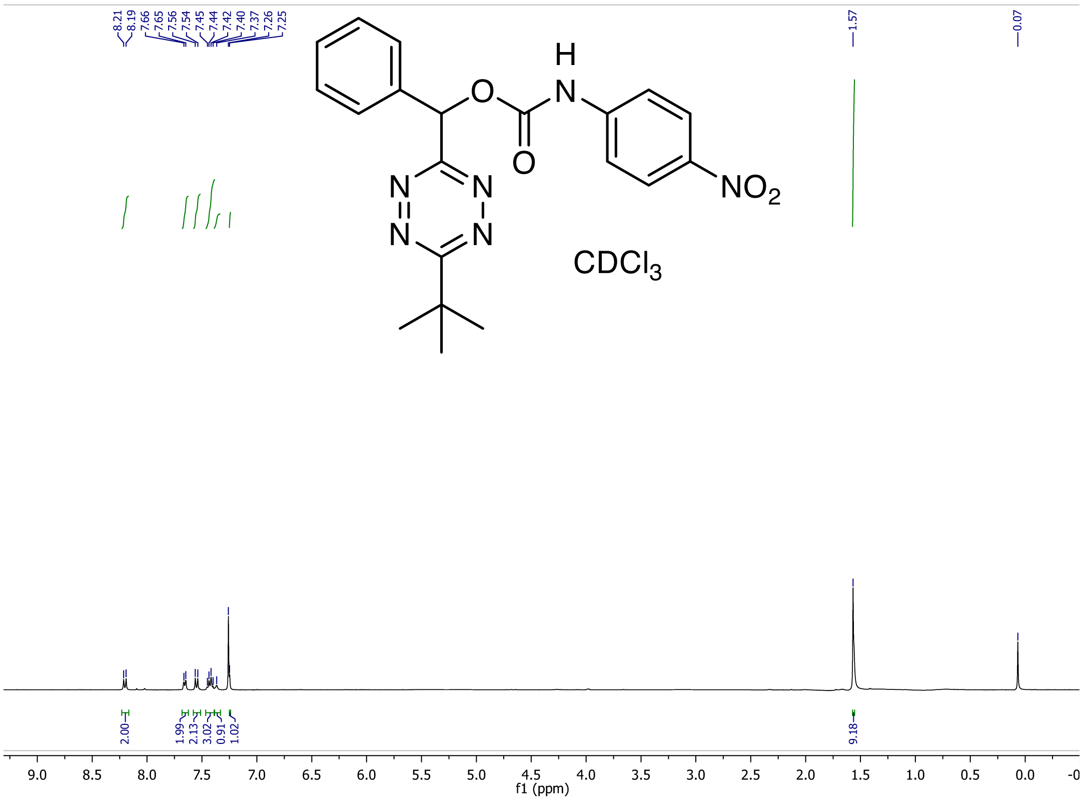


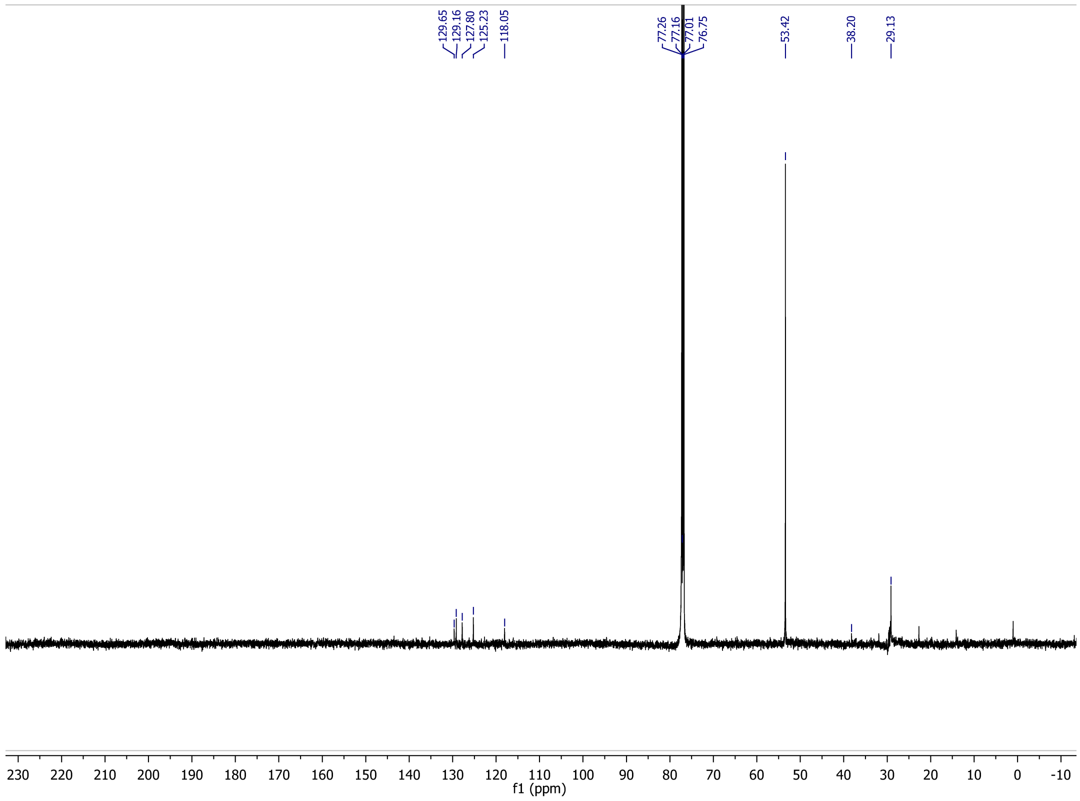

*(6-phenyl-1,2,4,5-tetrazin-3-yl)methyl (4-nitrophenyl)carbamate (**4e**)*

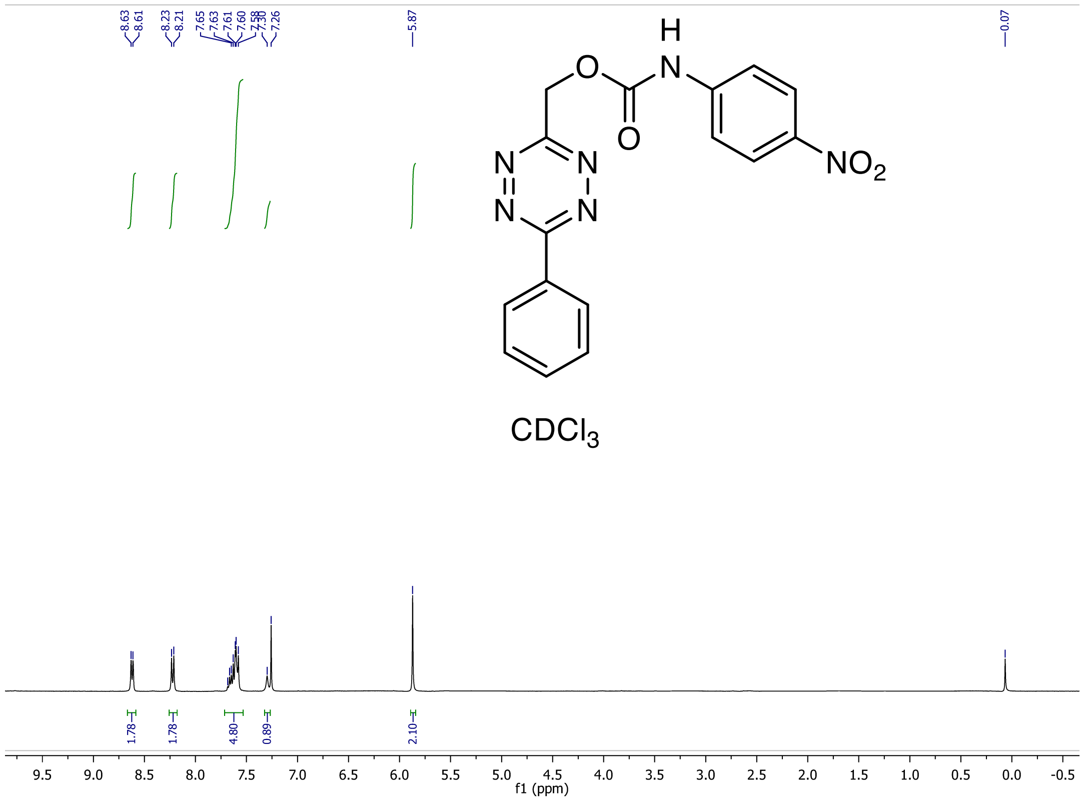


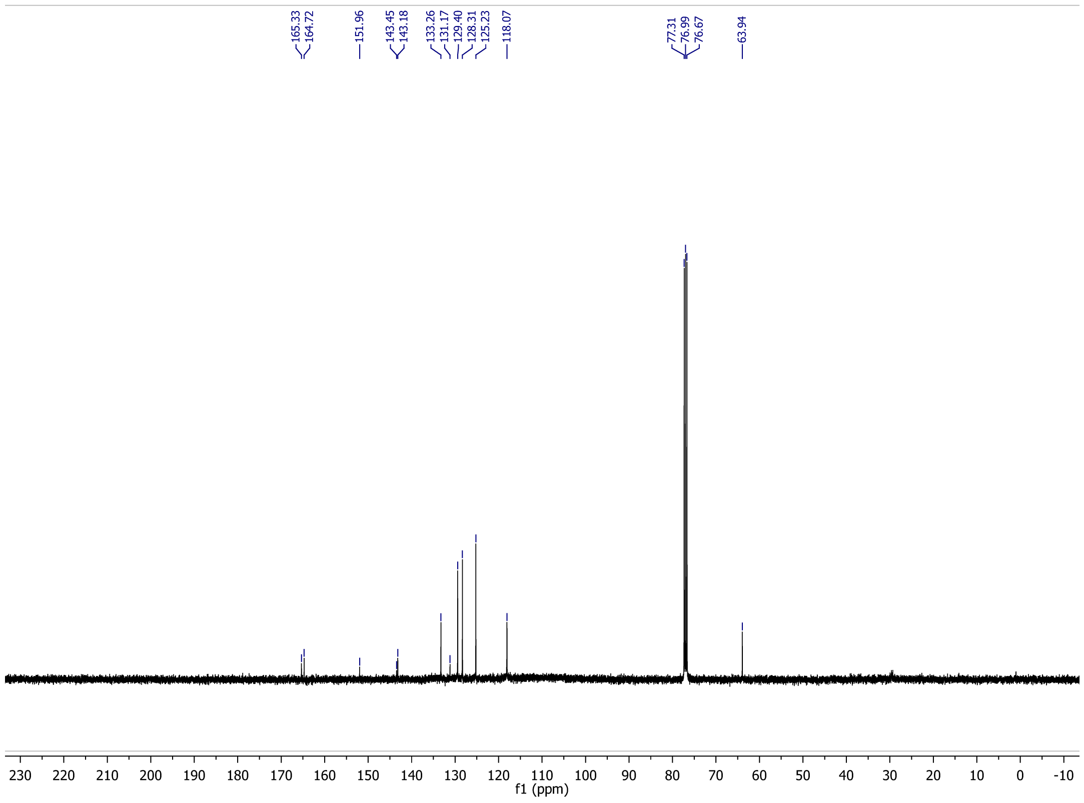

*3-(bromomethyl)-6-(tert-butyl)-1,2,4,5-tetrazine (**3**)*

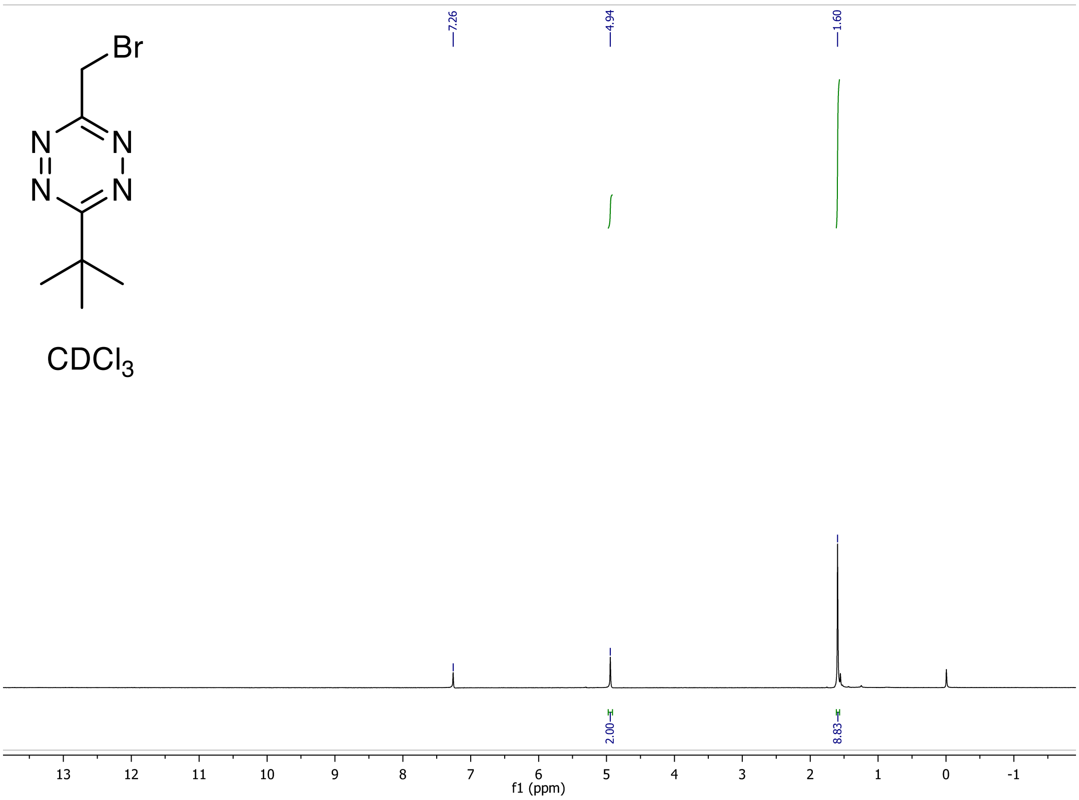


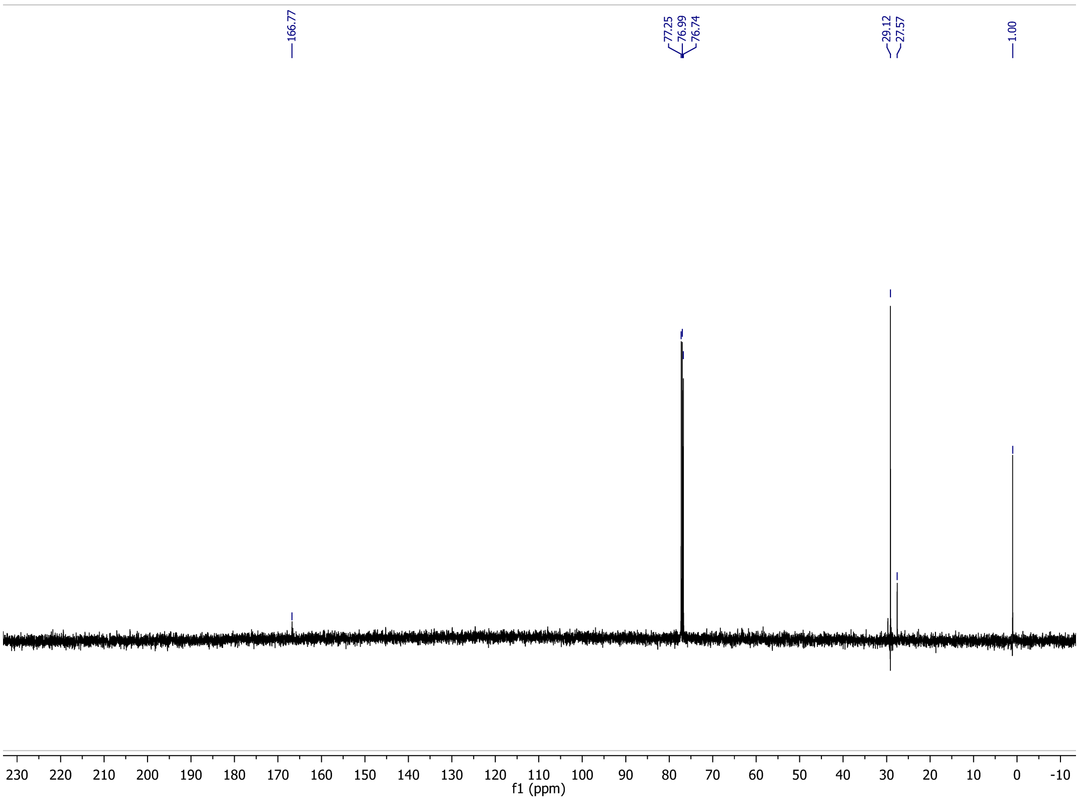

*tert-butyl2-((3'-((6-(tert-butyl)-1,2,4,5-tetrazin-3-yl)methoxy)-3-oxo-3H-spiro[isobenzofuran-1,9'-xanthen]-6'-yl)oxy)acetate*

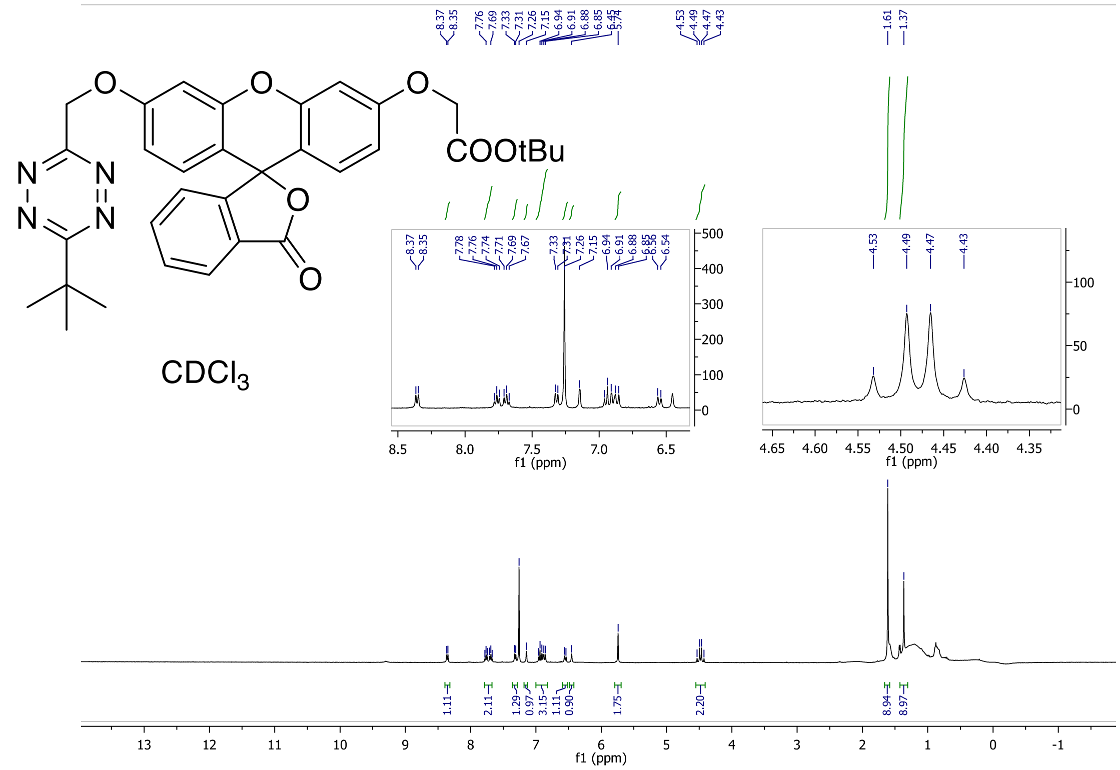


*2-((3'-((6-(tert-butyl)-1,2,4,5-tetrazin-3-yl)methoxy)-3-oxo-3H-spiro[isobenzofuran-1,9'-xanthen]-6'-yl)oxy)acetic acid (**4b**)*

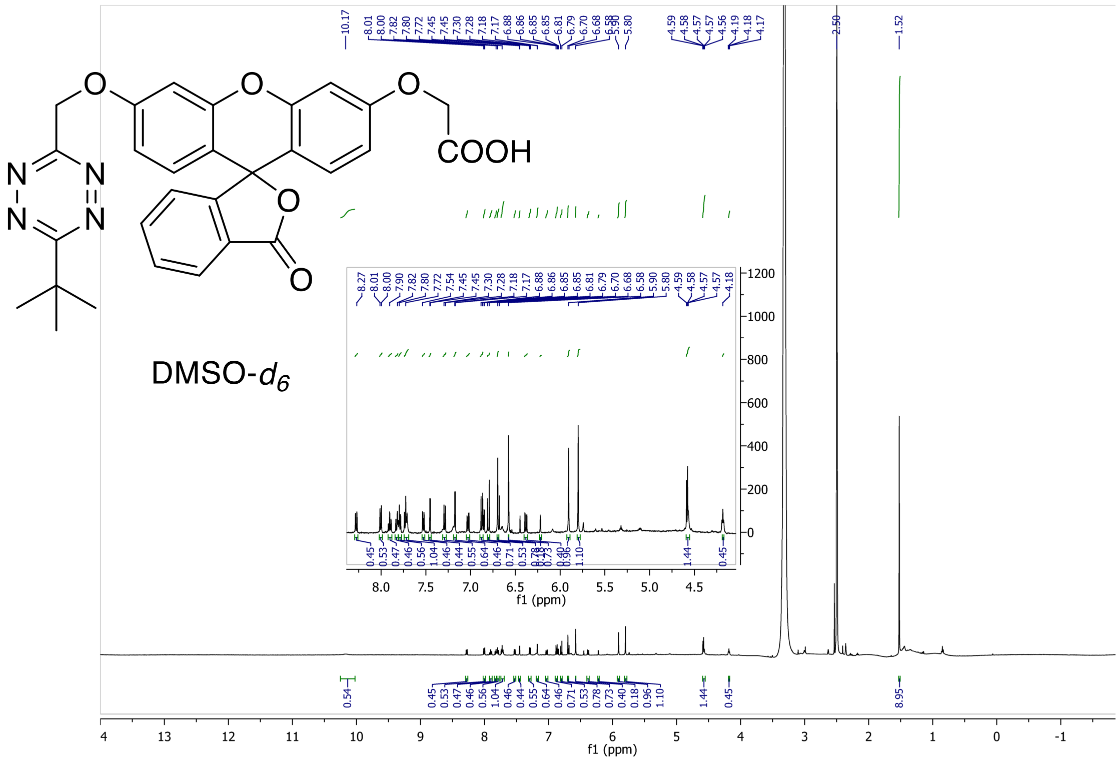

*7-((6-(tert-butyl)-1,2,4,5-tetrazin-3-yl)methoxy)-2H-chromen-2-one (**4b'**)*

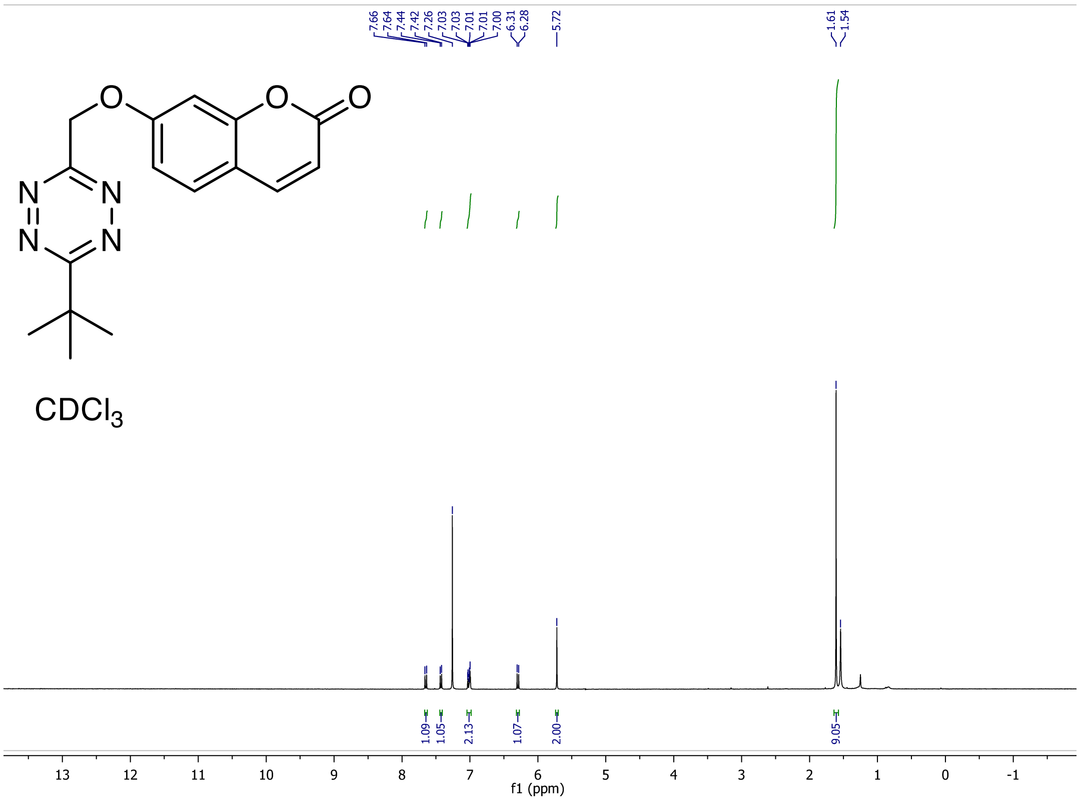


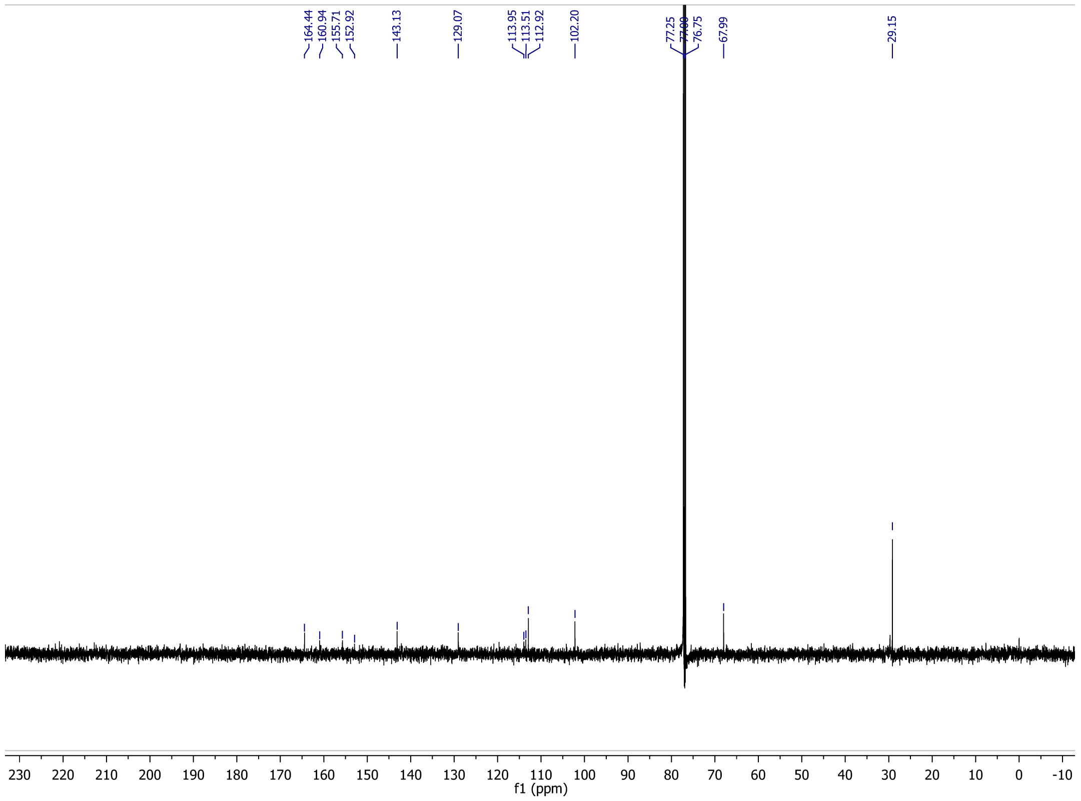

*3-(tert-butyl)-6-(doxorubicin)-1,2,4,5-tetrazine* ***(Tzmoc-Dox, 5)***

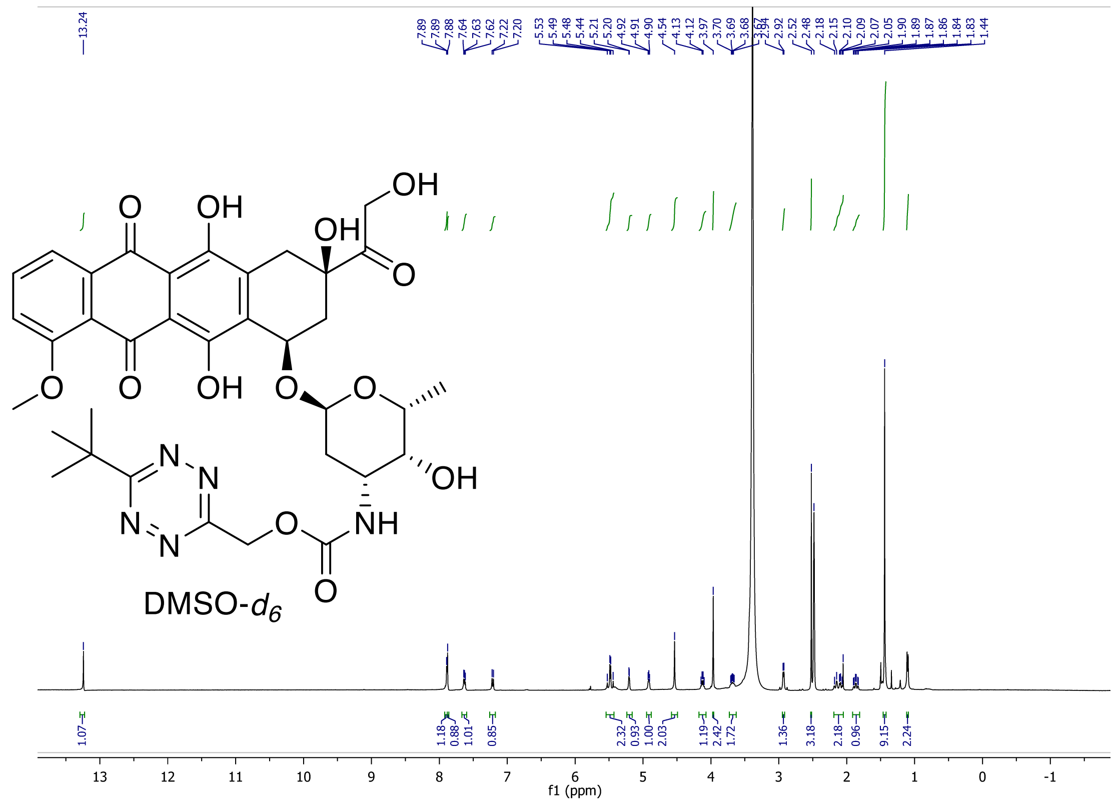

# Cartesian Coordinates

**H2O**

| | | | |
|---|---|---|---|
| O | 0.000000 | 0.000000 | 0.112145 |
| H | 0.000000 | 0.794627 | -0.448582 |
| H | 0.000000 | -0.794627 | -0.448582 |

**$OH^-$**

| | | | |
|---|---|---|---|
| O | 0.000000 | 0.000000 | 0.108656 |
| H | 0.000000 | 0.000000 | -0.869250 |

**$H_3O^+$**

| | | | |
|---|---|---|---|
| O | -0.000145 | -0.000110 | -0.042136 |
| H | 0.313593 | 0.908550 | 0.112234 |
| H | -0.944000 | -0.183008 | 0.112503 |
| H | 0.631565 | -0.724666 | 0.112353 |

**TMS-MeNC**

| | | | |
|---|---|---|---|
| C | 3.013679 | 0.234965 | -0.012910 |
| N | 2.060454 | -0.440951 | 0.037938 |
| C | 0.833397 | -1.170285 | 0.087929 |
| H | 0.799045 | -1.744453 | 1.013242 |
| H | 0.803859 | -1.869653 | -0.747299 |
| Si | -0.659886 | 0.048346 | -0.005108 |
| C | -0.447420 | 1.049827 | -1.590110 |
| H | -1.246871 | 1.784354 | -1.696152 |
| H | 0.503003 | 1.586628 | -1.575976 |
| H | -0.461451 | 0.403025 | -2.468231 |
| C | -0.559609 | 1.170240 | 1.510078 |
| H | -1.372659 | 1.897386 | 1.507695 |
| H | -0.624646 | 0.589453 | 2.430981 |
| H | 0.382804 | 1.720373 | 1.518823 |
| C | -2.226796 | -1.002391 | -0.015743 |
| H | -2.289122 | -1.613849 | 0.885358 |
| H | -3.115100 | -0.370883 | -0.057337 |
| H | -2.243150 | -1.666694 | -0.880629 |

**TMSOH**

| | | | |
|---|---|---|---|
| Si | 0.001664 | 0.000003 | -0.011068 |
| C | -0.922801 | 1.549296 | -0.533959 |
| C | 1.785582 | -0.000086 | -0.587287 |
| C | -0.922987 | -1.549161 | -0.534033 |
| H | 1.842155 | -0.000063 | -1.676314 |
| H | 2.307052 | 0.884030 | -0.219506 |
| H | 2.306953 | -0.884283 | -0.219556 |
| H | -0.988283 | 1.606716 | -1.621670 |
| H | -0.412429 | 2.444540 | -0.177765 |
| H | -1.938442 | 1.551085 | -0.136130 |
| H | -0.412702 | -2.444485 | -0.177918 |
| H | -1.938611 | -1.550850 | -0.136156 |
| H | -0.988535 | -1.606499 | -1.621743 |
| O | 0.148624 | -0.000066 | 1.721138 |
| H | -0.628209 | 0.000002 | 2.304271 |

**$TMSOH_2^+$**

| | | | |
|---|---|---|---|
| Si | -0.036753 | -0.000002 | -0.157235 |
| C | -0.998317 | 1.575031 | -0.409602 |
| C | 1.711405 | 0.000021 | -0.801485 |
| C | -0.998269 | -1.575068 | -0.409584 |
| H | 1.712587 | 0.000017 | -1.891916 |
| H | 2.248807 | 0.886323 | -0.465003 |
| H | 2.248834 | -0.886262 | -0.464997 |
| H | -1.266020 | 1.689224 | -1.460734 |
| H | -0.403129 | 2.439060 | -0.115671 |
| H | -1.918912 | 1.570451 | 0.173806 |
| H | -0.403060 | -2.439074 | -0.115630 |
| H | -1.918869 | -1.570502 | 0.173815 |
| H | -1.265957 | -1.689289 | -1.460716 |
| O | 0.298620 | 0.000016 | 1.647851 |
| H | -0.392590 | 0.000036 | 2.336315 |
| H | 1.194978 | 0.000003 | 2.033247 |

**A1**

| | | | |
|---|---|---|---|
| N | -3.385943 | 0.094732 | 0.528279 |
| N | -2.866954 | -1.304343 | 0.451381 |
| C | -1.685483 | -1.290290 | -0.087286 |
| C | -2.490614 | 0.883089 | 0.020868 |
| C | -1.306120 | 0.112879 | -0.423037 |
| C | -2.632988 | 2.352060 | -0.084866 |
| C | -0.911255 | -2.534783 | -0.320211 |
| N | -0.273622 | 0.661261 | -0.948504 |
| C | 0.933173 | -0.031561 | -1.299991 |
| H | -0.723117 | -2.678548 | -1.385141 |
| H | 0.052969 | -2.500920 | 0.186755 |
| H | -1.472241 | -3.388835 | 0.052102 |
| H | -2.585784 | 2.664848 | -1.128583 |

| | | | |
|---|---|---|---|
| H | -3.582036 | 2.671974 | 0.337288 |
| H | -1.821810 | 2.853655 | 0.443789 |
| H | 0.813358 | -1.089091 | -1.529260 |
| H | 1.397251 | 0.477854 | -2.142912 |
| Si | 2.222040 | 0.154146 | 0.155377 |
| C | 1.497072 | -0.623328 | 1.716475 |
| C | 3.767620 | -0.749084 | -0.437845 |
| C | 2.548733 | 1.995355 | 0.405231 |
| H | 3.559159 | -1.805785 | -0.609086 |
| H | 4.134485 | -0.316125 | -1.369114 |
| H | 4.561440 | -0.675622 | 0.307034 |
| H | 0.437621 | -0.384296 | 1.832227 |
| H | 1.605818 | -1.707805 | 1.703339 |
| H | 2.018970 | -0.243185 | 2.596235 |
| H | 2.835069 | 2.469579 | -0.534370 |
| H | 1.664922 | 2.504343 | 0.790454 |
| H | 3.360170 | 2.142355 | 1.119854 |

**A2**

| | | | |
|---|---|---|---|
| C | 0.031394 | 0.337778 | -0.008518 |
| C | 0.702823 | -0.910027 | 0.000901 |
| N | -0.206704 | -1.910799 | 0.010384 |
| N | -1.497328 | -1.331070 | 0.011815 |
| C | -1.340058 | 0.009618 | -0.002826 |
| N | 0.491513 | 1.664604 | -0.030888 |
| C | 1.732570 | 1.999226 | 0.026252 |
| H | 1.980799 | 3.052506 | -0.001976 |
| H | 2.556604 | 1.298073 | 0.109604 |
| C | -2.501666 | 0.944940 | -0.000026 |
| H | -2.534311 | 1.546409 | 0.910333 |
| H | -2.464621 | 1.636124 | -0.843224 |
| H | -3.430987 | 0.380135 | -0.063186 |
| C | 2.164526 | -1.211468 | -0.014569 |
| H | 2.661226 | -0.767884 | -0.880197 |
| H | 2.669069 | -0.834867 | 0.877694 |
| H | 2.312325 | -2.290040 | -0.055502 |

**A3**

| | | | |
|---|---|---|---|
| N | 1.504942 | -1.290898 | 0.088023 |
| N | 0.220325 | -1.801801 | 0.068883 |
| C | -0.732726 | -0.841147 | -0.012926 |
| C | 1.340301 | 0.037782 | -0.001529 |
| C | -0.038439 | 0.367570 | -0.056788 |
| C | 2.494865 | 0.974067 | -0.022033 |
| C | -2.181711 | -1.167926 | -0.105401 |
| N | -0.510902 | 1.690566 | -0.186494 |
| C | -1.694830 | 2.019262 | 0.182795 |
| H | -2.671575 | -0.512507 | -0.826471 |
| H | -2.318625 | -2.198648 | -0.429103 |
| H | -2.685697 | -1.042169 | 0.853264 |
| H | 3.427994 | 0.415782 | 0.018496 |
| H | 2.465777 | 1.658178 | 0.826170 |
| H | 2.492996 | 1.576823 | -0.930758 |
| H | -2.393870 | 1.336674 | 0.657660 |
| H | -2.016263 | 3.041718 | 0.036436 |
| H | 0.073955 | -2.798567 | 0.096711 |

**B1**

| | | | |
|---|---|---|---|
| N | -1.946311 | 1.302814 | -0.000007 |
| N | -2.464981 | -0.105208 | -0.000007 |
| C | -1.452519 | -0.909525 | -0.000058 |
| C | -0.651854 | 1.277378 | -0.000010 |
| C | -0.174968 | -0.144892 | -0.000223 |
| N | 0.978071 | -0.691947 | -0.000235 |
| C | 2.224424 | 0.099677 | -0.000182 |
| H | 2.234039 | 0.746996 | 0.878168 |
| H | 2.234411 | 0.746647 | -0.878787 |
| C | 0.167865 | 2.512187 | 0.000096 |
| H | 0.809954 | 2.553760 | 0.880465 |
| H | 0.809818 | 2.553997 | -0.880360 |
| H | -0.482986 | 3.383165 | 0.000257 |
| C | -1.562807 | -2.384545 | 0.000139 |
| H | -1.074619 | -2.805783 | -0.878984 |
| H | -1.075580 | -2.805399 | 0.879996 |
| H | -2.608109 | -2.682492 | -0.000332 |
| C | 3.439393 | -0.806941 | 0.000268 |
| H | 3.450348 | -1.442738 | -0.884311 |
| H | 4.348081 | -0.205444 | 0.000324 |
| H | 3.449977 | -1.442346 | 0.885133 |

**B2**

| | | | |
|---|---|---|---|
| C | 0.189066 | -0.122610 | -0.038826 |
| C | 0.635801 | 1.218560 | 0.001017 |
| N | 1.990620 | 1.241118 | 0.058940 |
| N | 2.449017 | -0.092117 | 0.071060 |
| C | 1.363182 | -0.897471 | -0.000990 |
| N | -1.103675 | -0.684523 | -0.139933 |
| C | -2.171463 | -0.029000 | 0.149376 |

| | | | |
|---|---|---|---|
| H | -2.130839 | 0.989833 | 0.531937 |
| C | 1.480416 | -2.384524 | -0.019385 |
| H | 1.024707 | -2.841037 | 0.861207 |
| H | 0.992414 | -2.815337 | -0.895485 |
| H | 2.531248 | -2.671763 | -0.040453 |
| C | -0.143887 | 2.489913 | -0.086216 |
| H | -0.896461 | 2.441007 | -0.876396 |
| H | -0.665702 | 2.727009 | 0.843276 |
| H | 0.531372 | 3.315587 | -0.309421 |
| C | -3.532386 | -0.623896 | 0.003463 |
| H | -4.061136 | -0.614463 | 0.959193 |
| H | -4.133707 | -0.039775 | -0.697113 |
| H | -3.468003 | -1.648244 | -0.357846 |

**B3**

| | | | |
|---|---|---|---|
| N | 1.918693 | 1.293145 | 0.075921 |
| N | 2.336390 | -0.022183 | 0.090037 |
| C | 1.308479 | -0.898754 | -0.005745 |
| C | 0.577904 | 1.229860 | -0.010949 |
| C | 0.152763 | -0.129529 | -0.062697 |
| N | -1.133790 | -0.698986 | -0.194964 |
| C | -2.188140 | -0.071142 | 0.186031 |
| H | -2.127829 | 0.903534 | 0.667553 |
| C | -0.230166 | 2.477506 | -0.108503 |
| H | -0.993560 | 2.386006 | -0.882454 |
| H | -0.737330 | 2.710444 | 0.828499 |
| H | 0.417510 | 3.315518 | -0.359603 |
| C | 1.493701 | -2.373431 | -0.027352 |
| H | 1.156694 | -2.827340 | 0.905002 |
| H | 0.919031 | -2.818422 | -0.838801 |
| H | 2.543092 | -2.626325 | -0.169262 |
| H | 3.318528 | -0.238440 | 0.152756 |
| C | -3.552047 | -0.645122 | 0.020840 |
| H | -4.051483 | -0.719872 | 0.988994 |
| H | -4.165840 | 0.006332 | -0.605021 |
| H | -3.502829 | -1.631599 | -0.434377 |

**[A1+H]$^+$**

| | | | |
|---|---|---|---|
| N | 2.812157 | 1.283570 | 0.452205 |
| N | 3.223845 | -0.084684 | 0.480674 |
| C | 2.356819 | -0.915922 | -0.032178 |
| C | 1.626251 | 1.289779 | -0.102227 |
| C | 1.233667 | -0.089289 | -0.460335 |
| C | 0.883271 | 2.553150 | -0.314457 |
| C | 2.509375 | -2.377667 | -0.120716 |
| N | 0.186355 | -0.611537 | -1.020001 |
| C | -0.979215 | 0.085822 | -1.345840 |
| H | 3.399811 | -2.632933 | -0.696032 |
| H | 2.622527 | -2.804355 | 0.876739 |
| H | 1.635958 | -2.810845 | -0.597695 |
| H | -0.078386 | 2.534384 | 0.199664 |
| H | 0.692174 | 2.710048 | -1.376882 |
| H | 1.465740 | 3.389240 | 0.063745 |
| H | -0.892460 | 1.158597 | -1.500837 |
| H | -1.513681 | -0.410700 | -2.152013 |
| Si | -2.232664 | -0.165521 | 0.201599 |
| C | -2.991578 | -1.872721 | -0.004611 |
| C | -1.209509 | -0.015144 | 1.771432 |
| C | -3.457678 | 1.243503 | -0.033212 |
| H | -0.630962 | 0.909573 | 1.791759 |
| H | -0.521040 | -0.854668 | 1.874324 |
| H | -1.867913 | -0.012767 | 2.641966 |
| H | -3.677782 | -2.086459 | 0.816175 |
| H | -2.216599 | -2.639604 | -0.007889 |
| H | -3.547829 | -1.938609 | -0.939634 |
| H | -2.964850 | 2.206926 | 0.099635 |
| H | -3.891073 | 1.211768 | -1.033082 |
| H | -4.267214 | 1.168093 | 0.694106 |
| H | 4.125964 | -0.300894 | 0.886281 |

**[B1+H]$^+$**

| | | | |
|---|---|---|---|
| N | 2.435676 | -0.221127 | -0.000162 |
| N | 1.926085 | 1.132094 | -0.000052 |
| C | 0.635482 | 1.242157 | 0.000049 |
| C | 1.383516 | -0.983592 | -0.000100 |
| C | 0.138689 | -0.169857 | 0.000000 |
| N | -1.037031 | -0.664449 | 0.000020 |
| C | -2.257360 | 0.155826 | 0.000137 |
| H | -2.241707 | 0.805262 | -0.878254 |
| H | -2.241701 | 0.805056 | 0.878681 |
| C | 1.447196 | -2.456533 | -0.000158 |
| H | 0.943008 | -2.855617 | 0.879885 |
| H | 0.942897 | -2.855546 | -0.880170 |
| H | 2.481319 | -2.788907 | -0.000236 |
| C | -0.092851 | 2.518838 | 0.000171 |
| H | -0.737689 | 2.577123 | -0.878916 |
| H | -0.737537 | 2.577047 | 0.879374 |
| H | 0.597406 | 3.358000 | 0.000150 |

| | | | |
|---|---|---|---|
| H | 2.604092 | 1.887451 | -0.000060 |
| C | -3.496204 | -0.715328 | 0.000042 |
| H | -4.385506 | -0.086384 | 0.000099 |
| H | -3.524237 | -1.349001 | -0.885208 |
| H | -3.524251 | -1.349180 | 0.885163 |

**TS$_{A1>A2}$**

| | | | |
|---|---|---|---|
| N | -3.577221 | 0.346246 | 0.780575 |
| N | -3.242136 | -1.070128 | 0.743202 |
| C | -2.129202 | -1.220146 | 0.049037 |
| C | -2.661379 | 0.995782 | 0.088696 |
| C | -1.673336 | 0.076494 | -0.426296 |
| C | -2.667995 | 2.470614 | -0.086122 |
| C | -1.536603 | -2.562564 | -0.193620 |
| N | -0.636300 | 0.458660 | -1.162616 |
| C | 0.394684 | -0.326750 | -1.533736 |
| H | -1.447496 | -2.762422 | -1.263299 |
| H | -0.537877 | -2.645676 | 0.239418 |
| H | -2.170638 | -3.328061 | 0.249361 |
| H | -2.700305 | 2.738878 | -1.142639 |
| H | -3.533785 | 2.903298 | 0.410221 |
| H | -1.766299 | 2.916157 | 0.336196 |
| H | 0.326510 | -1.408105 | -1.464127 |
| H | 0.975948 | 0.059424 | -2.363405 |
| Si | 2.039377 | 0.069261 | -0.041310 |
| C | 1.073736 | -0.440458 | 1.490953 |
| C | 3.153217 | -1.191361 | -0.900697 |
| C | 2.240092 | 1.892064 | -0.471378 |
| H | 3.242331 | -2.078581 | -0.272138 |
| H | 2.773791 | -1.494292 | -1.873927 |
| H | 4.149731 | -0.769191 | -1.033245 |
| H | 0.006138 | -0.545164 | 1.307898 |
| H | 1.451704 | -1.395442 | 1.859405 |
| H | 1.204014 | 0.306215 | 2.274687 |
| H | 1.796576 | 2.131431 | -1.435471 |
| H | 1.756573 | 2.503440 | 0.291966 |
| H | 3.297168 | 2.156036 | -0.495628 |
| O | 3.674148 | 0.372204 | 1.377298 |
| H | 4.580989 | 0.563796 | 1.079603 |
| H | 3.680786 | -0.104534 | 2.225927 |

**TS'$_{A1>A2}$**

| | | | |
|---|---|---|---|
| N | -3.610403 | 0.283878 | 0.762719 |
| N | -3.197616 | -1.141076 | 0.738161 |
| C | -2.078849 | -1.246219 | 0.076976 |
| C | -2.726887 | 0.970028 | 0.097217 |
| C | -1.659915 | 0.091344 | -0.408041 |
| C | -2.787631 | 2.438419 | -0.086376 |
| C | -1.418601 | -2.558687 | -0.140437 |
| N | -0.653933 | 0.521287 | -1.095417 |
| C | 0.456432 | -0.256626 | -1.477563 |
| H | -1.352035 | -2.785528 | -1.205857 |
| H | -0.405569 | -2.565514 | 0.263605 |
| H | -1.994401 | -3.342485 | 0.346624 |
| H | -2.803729 | 2.694530 | -1.145963 |
| H | -3.679226 | 2.840695 | 0.387924 |
| H | -1.909988 | 2.914799 | 0.352263 |
| H | 0.299417 | -1.331676 | -1.533101 |
| H | 0.887777 | 0.129752 | -2.399043 |
| Si | 1.970799 | 0.043054 | -0.165161 |
| C | 1.163092 | -0.396526 | 1.489322 |
| C | 3.195689 | -1.198072 | -0.904059 |
| C | 2.345052 | 1.879180 | -0.435611 |
| H | 3.313922 | -2.048016 | -0.231216 |
| H | 2.857119 | -1.568762 | -1.872179 |
| H | 4.170918 | -0.730482 | -1.029160 |
| H | 0.078814 | -0.488597 | 1.414405 |
| H | 1.562157 | -1.341102 | 1.859757 |
| H | 1.382044 | 0.370876 | 2.231014 |
| H | 1.787022 | 2.273989 | -1.284989 |
| H | 2.073996 | 2.457451 | 0.449159 |
| H | 3.408709 | 2.024191 | -0.616479 |
| O | 4.059150 | 0.356451 | 1.527599 |
| H | 3.562037 | 1.055852 | 1.997894 |

**TS$_{B1>B2}$**

| | | | |
|---|---|---|---|
| N | 2.192623 | 1.379940 | -0.038237 |
| N | 2.768080 | 0.073928 | 0.229659 |
| C | 1.796880 | -0.818849 | 0.152883 |
| C | 0.903820 | 1.225949 | -0.291107 |
| C | 0.552944 | -0.177412 | -0.192597 |
| N | -0.596137 | -0.836670 | -0.334610 |
| C | -1.794325 | -0.284777 | -0.620732 |
| H | -1.820791 | 0.701020 | -1.083430 |
| H | -2.145184 | 0.146872 | 0.673870 |
| C | 0.019144 | 2.387682 | -0.570883 |
| H | -0.403299 | 2.334167 | -1.576170 |
| H | -0.815248 | 2.425323 | 0.132837 |

| | | | |
|---|---|---|---|
| H | 0.587734 | 3.311585 | -0.485295 |
| C | 2.013551 | -2.271580 | 0.378896 |
| H | 1.731307 | -2.852953 | -0.499290 |
| H | 3.062061 | -2.463021 | 0.597102 |
| H | 1.414235 | -2.630658 | 1.216510 |
| O | -2.500651 | 0.543687 | 1.755251 |
| H | -2.592656 | 1.509570 | 1.849118 |
| H | -3.236163 | 0.049825 | 2.161065 |
| C | -2.932568 | -1.207049 | -0.986744 |
| H | -2.918282 | -2.105170 | -0.369577 |
| H | -3.889722 | -0.706210 | -0.840526 |
| H | -2.877426 | -1.514009 | -2.034203 |

**TS$_{[A1+H]+>A3}$**

| | | | |
|---|---|---|---|
| N | 3.454878 | -0.315556 | 0.815086 |
| N | 3.165501 | 1.075301 | 0.854565 |
| C | 2.089486 | 1.234458 | 0.115996 |
| C | 2.619881 | -1.013282 | 0.081983 |
| C | 1.663169 | -0.059632 | -0.429952 |
| C | 2.678708 | -2.471938 | -0.121317 |
| C | 1.499069 | 2.575604 | -0.101329 |
| N | 0.673122 | -0.436144 | -1.204080 |
| C | -0.367358 | 0.362011 | -1.606392 |
| H | 0.474192 | 2.617830 | 0.269804 |
| H | 2.090316 | 3.326503 | 0.416450 |
| H | 1.479202 | 2.815253 | -1.165598 |
| H | 3.519741 | -2.905843 | 0.412760 |
| H | 2.772487 | -2.695097 | -1.184068 |
| H | 1.754631 | -2.930879 | 0.231184 |
| H | -0.249905 | 1.441108 | -1.571415 |
| H | -0.851457 | -0.009431 | -2.505492 |
| Si | -1.894093 | -0.034555 | -0.251726 |
| C | -2.920068 | 1.502150 | -0.606199 |
| C | -2.566907 | -1.675044 | -0.870883 |
| C | -1.012432 | -0.080622 | 1.406644 |
| H | -3.215128 | -2.132178 | -0.125790 |
| H | -3.143440 | -1.524650 | -1.784055 |
| H | -1.750817 | -2.363237 | -1.090543 |
| H | -3.906789 | 1.423662 | -0.154280 |
| H | -3.041865 | 1.632879 | -1.681947 |
| H | -2.419202 | 2.385727 | -0.209356 |
| H | -0.409230 | -0.984610 | 1.500263 |
| H | -0.354275 | 0.782712 | 1.520581 |
| H | -1.731804 | -0.062821 | 2.223852 |

| | | | |
|---|---|---|---|
| O | -4.227194 | -0.434357 | 1.556235 |
| H | -3.878554 | -1.128535 | 2.143700 |
| H | 4.253040 | -0.657651 | 1.334482 |
| H | -4.212092 | 0.420439 | 2.023456 |

**TS$_{[B1+H]+>B3}$**

| | | | |
|---|---|---|---|
| N | -2.124000 | 1.426762 | -0.082638 |
| N | -2.656889 | 0.145405 | 0.195705 |
| C | -1.762957 | -0.821667 | 0.163503 |
| C | -0.842315 | 1.230902 | -0.319726 |
| C | -0.521897 | -0.188744 | -0.188031 |
| N | 0.599848 | -0.878109 | -0.303078 |
| C | 1.824842 | -0.346697 | -0.590149 |
| H | 2.166995 | 0.095294 | 0.564877 |
| H | 1.841074 | 0.595570 | -1.137392 |
| C | 0.067183 | 2.361514 | -0.617572 |
| H | 0.900495 | 2.379035 | 0.087144 |
| H | 0.484133 | 2.272654 | -1.622077 |
| H | -0.475345 | 3.300826 | -0.546917 |
| C | -2.049245 | -2.244948 | 0.431187 |
| H | -1.457034 | -2.589833 | 1.278927 |
| H | -3.103321 | -2.394847 | 0.650401 |
| H | -1.776468 | -2.851050 | -0.432259 |
| H | -3.643232 | 0.064367 | 0.403593 |
| O | 2.613266 | 0.617910 | 1.741459 |
| H | 2.435609 | 1.571717 | 1.837502 |
| H | 3.539195 | 0.403605 | 1.956874 |
| C | 2.922900 | -1.318265 | -0.959459 |
| H | 3.892699 | -0.829529 | -0.875440 |
| H | 2.915848 | -2.183885 | -0.298507 |
| H | 2.809444 | -1.668182 | -1.986832 |

**[TMS-MeNC-H]$^-$**

| | | | |
|---|---|---|---|
| C | 0.042333 | 1.414390 | 0.000000 |
| N | 0.000000 | 0.234780 | 0.000000 |
| C | -0.132946 | -1.155502 | 0.000000 |
| H | 0.271840 | -1.598394 | 0.907658 |
| H | 0.271840 | -1.598394 | -0.907658 |
